# Modeling the ribosome as a bipartite graph

**DOI:** 10.1371/journal.pone.0279455

**Published:** 2022-12-30

**Authors:** Laurie E. Calvet

**Affiliations:** CNRS, Centre de Nanosciences et Nanotechnologies, Université Paris-Saclay, Palaiseau, France; The University of Alabama in Huntsville, UNITED STATES

## Abstract

Developing mathematical representations of biological systems that can allow predictions is a challenging and important research goal. It is demonstrated here how the ribosome, the nano-machine responsible for synthesizing all proteins necessary for cellular life, can be represented as a bipartite network. Ten ribosomal structures from Bacteria and six from Eukarya are explored. Ribosomal networks are found to exhibit unique properties despite variations in the nodes and edges of the different graphs. The ribosome is shown to exhibit very large topological redundancies, demonstrating mathematical resiliency. These results can potentially explain how it can function consistently despite changes in composition and connectivity. Furthermore, this representation can be used to analyze ribosome function within the large machinery of network theory, where the degrees of freedom are the possible interactions, and can be used to provide new insights for translation regulation and therapeutics.

## 1 Introduction

The ribosome, responsible for the coded synthesis of all cellular proteins, is the ultimate nano-machine. It is comprised of two particles, referred to as the small (SSU) and large (LSU) subunits. Each subunit contains ribosomal ribonucleic acid rRNA and proteins. An active ribosome is formed when a messenger (m)RNA binds to the SSU and then with the LSU. The mRNA, which has been copied from a gene in the DNA, encodes a protein. This protein product is synthesized one residue at a time, corresponding to three codons on the mRNA, while the ribosome undergoes a cyclic series of transitions that are facilitated by proteins known as elongation factors. The ribosome is thus fundamentally dynamic, constantly changing and modifying its shape throughout the process of elongation [1].

About twenty years ago, the first near-atomic resolution crystal structures were published [2, 3], heralding in a new era for understanding the relationship between ribosome function and structure. However, there is not yet a synthetic mathematical representation that is able to succinctly describe ribosome structure. Such a framework would enhance the use of physical modeling, bringing a new understanding of translation and enabling new therapeutics.

In a previous study, a network model of the ribosome considered a representation as a unipartite graph [4], and explored changes in the ribosome at different stages in translation. The graphs were constructed by separating the ribosome into its molecular elements (proteins, rRNAs and rRNA domains for the largest molecules, mRNA, tRNAs) and determining the interactions by seeing whether a solvent molecule of 1.4 Å could be rolled between each two elements. However, a unipartite graph cannot account for the difference between elements interacting independently and those involved in the same interaction. This connectivity model is different from the much denser ribosomal networks considered previously, where the nodes consist of each residue/nucleotide. Contacts between nodes thus include neighboring residues on the molecule where they are found, as well as additional contacts formed by other residues [5–8]. The degrees of freedom of each node in these networks are subject to the collective constraints of the molecules. More traditional simulations of ribosome function initially used coarse grain models [9, 10] or very short molecular dynamics simulations [11, 12]. More powerful methods were also employed so as to overcome the difficulty of calculating such large atomic simulations by combining a minimal energy landscape with molecular dynamics simulations [13]. This class of models aims to understand the ribosome in its minute detail, including all possible physical phenomena (electrostatic, dynamic, chemical), but in relatively short time scales, and requires large computational capacity. The coarse grain models are computationally much less expensive but focus on a particular physical effect (elastic network).

**Fig 1:**
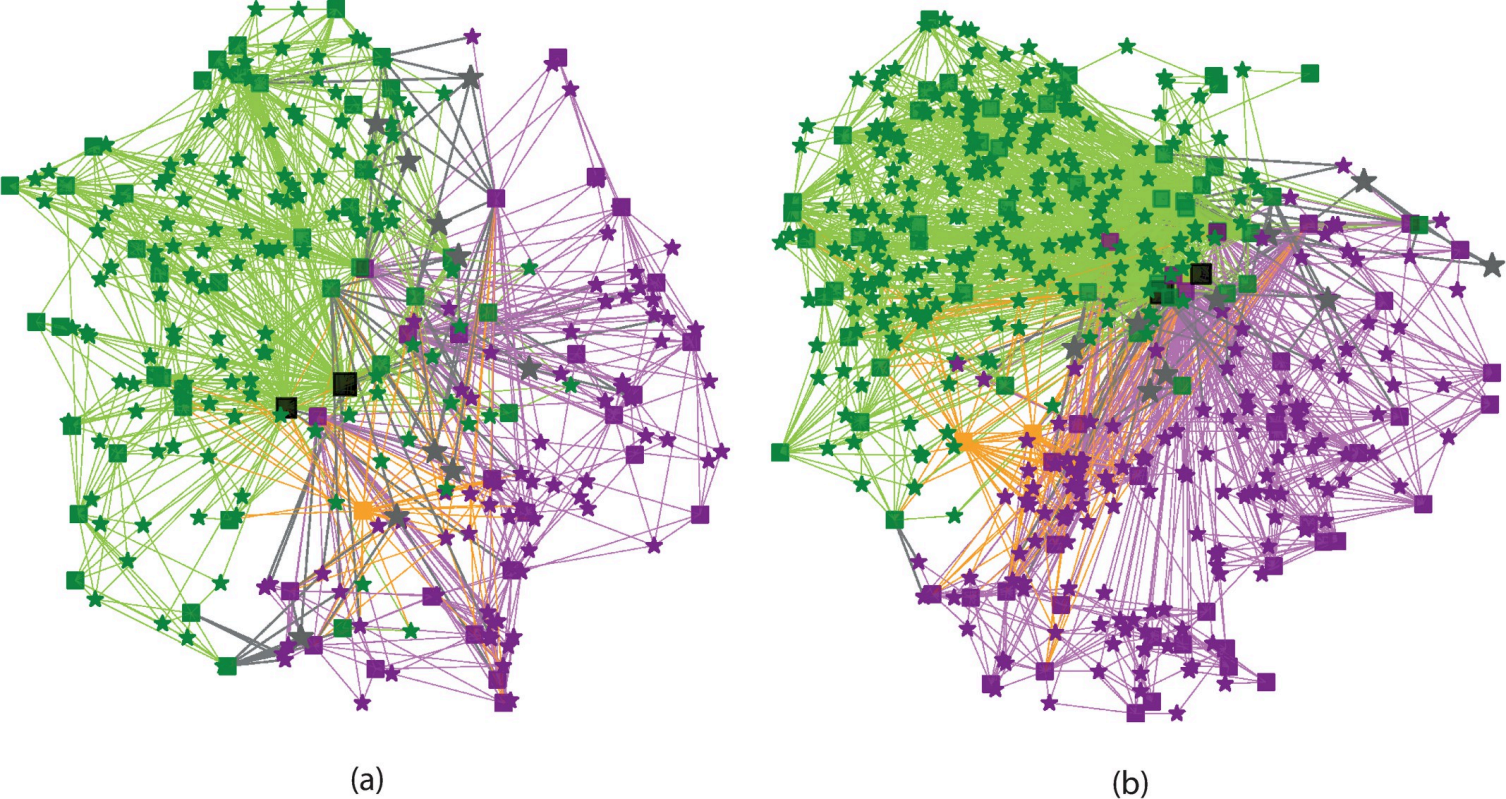
Visual representation of the bipartite network of (a) *T*. *thermophilus* from Bacteria [16] and (b) *S*. *cerevisiae* [17] from Eukarya. The squares represent ribosomal elements and stars the interactions. Green indicates elements and interactions in the LSU, where the peptide bond formation occurs. Magenta indicates elements and interactions in the SSU, where decoding takes place. Grey indicates inter-subunit bridges and orange indicates tRNAs. To emphasize domains 2 and 5 of 23(25)S rRNA, they are plotted respectively in black with the former twice as large as the other nodes to indicate its dominant centrality and the latter 50% larger. The positions of each node were obtained from the structural file by calculating the center of mass coordinates of the residues involved in the node. The three-dimensional coordinates were then projected into two-dimensions.

The premise here is that the ribosome can be mathematically modeled as a connectivity network. To accomplish this, the connectivity of the ribosome is represented as a bipartite graph, *B* = (*U*,*V*,*E*), which contains two disjoint sets of vertices *U* and *V* and the set of edges *E* connecting them [14, 15]. Fig 1 shows a graphical representation of the bipartite graphs of *Thermus thermophilus* and *Saccharomyces cerevisiae*, representative of Bacteria and Eukarya respective. Using this approach, the basic network characteristics of the ribosome are investigated and compared. To demonstrate its utility, we consider 10 high resolution structural files of Bacteria and 6 structural files of Eukarya. It is shown how the characteristics of this representation may provide new insights for ribosome evolution.

## 2 Methods and definitions

The use of bipartite graphs in biology and medicine are of increasing importance [14] and basic notions have been developed [15]. To construct the bipartite graph, the ribosome is first divided into individual molecular elements, which will constitute *U*, the first set of vertices. A natural division is to use the individual chains in the structural file, corresponding to individual proteins, rRNAs, mRNA and tRNAs. The ribosome consists of widely disparate RNA and protein elements. This creates a dilemma when creating a ribosomal graph as it is necessary to incorporate both the rRNAs and the ribosomal (r)proteins so that one can distinguish their different roles. Herein, the 16S (18S) and 23S (25S or 28S) rRNAs from Bacteria (Eukarya) are divided into homologous domains identified in the secondary structure [18] and denoted by D. All other elements are maintained as single nodes. Herein when ribosomal elements are mentioned, they refer to this first set of nodes in the bipartite graph that includes the individual elements or the individual domains of the rRNAs just mentioned. An example list of the ribosomal element nodes is provided in S1 Table. The residues involved in the domains can be found in the sample code of S1 File.

This representation could eventually be modified to include detailed secondary sub-structures of the rRNA such as helices and pseudo-knots, important functional centers such as pivot points [19], or divided into the evolutionary segment expansions [20, 21] to explore different aspects of the ribosome. Similarly, the rproteins could be divided into globular components and extensions [22]. Networks of specific motifs such as those with pi-conjugated bonds that might permit charge transfer [23, 24] could also be explored to consider its role in the functionality and communication in different parts of the ribosome.

**Fig 2:**
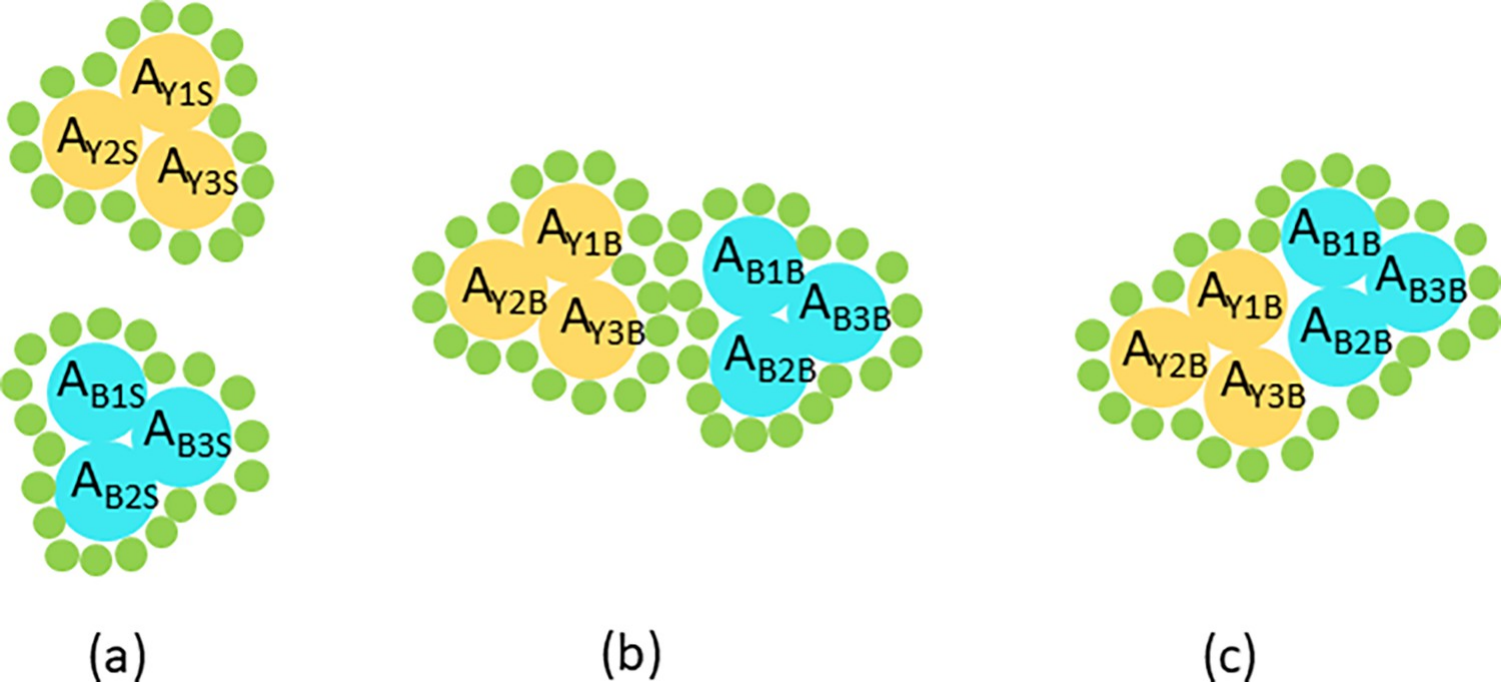
Schematic of the interaction calculation. Two elements are depicted here in yellow and blue with the individual circles representing the constituent residues. (a) The SASA of each residue is calculated for each element in isolation from the other elements in the ribosome. The three residues depicted here for each element are labeled by their color (B for Blue or Y for Yellow), an identifying number and an ‘S’ indicating that the SASA is calculated for a single element in isolation from all others in the ribosome. The additional radii due to a solvent molecule (green) of 1.4 Å located at its the surface is also indicated. (b) The SASA of two elements together and the solvent molecule is then calculated in the same way. Note that the final identifying label of the residues is ‘B’, indicating that the SASA is calculated for both elements together. The figure illustrates when an interaction is not present the difference in the SASA from the two individual elements is equal to that of the SASA when the two elements are both present. (c) Here an interaction is observed because although the area of residue *A*_*Y2S*_ *= A*_*Y2B*_, for the other residues: *A*_*Y1S*_ *> A*_*Y1B*_ and *A*_*Y3S*_ *> A*_*Y3B*_.

The second set of nodes in the bipartite graph is the interactions formed between ribosomal elements. First, all the dyadic interactions *v*_12_∈*V* formed between two elemental nodes *u*_1_, *u*_2_∈*U* are calculated. This calculation is derived from initial work by Sobolev *et al*. and reviewed in ref [25] and is illustrated in Fig 2. A dyadic interaction here means that a solvent molecule with a radius of 1.4 Å cannot fit at some point in space between two ribosomal elements. The advantage of this method is that it involves relatively low intensive calculations to determine interactions, as now described. First, the Solvent Accessible Surface Area [SASA] for each residue of each ribosomal element is calculated independently from the rest of the ribosomal structure. The SASA consists of the surface area of each residue plus that of the small solvent molecule. Then, the total SASA for each set of two ribosomal elements is determined. If a specific residue of the SASA determined from the two element set is smaller than that of an isolated element, then its SASA is partially obstructed by the other element. An interaction is therefore present. The calculation of the SASA was done using the method of Shrake and Rupley [26], in pymol [27]. Extensions of this calculation can be envisioned to explore the nature of the interactions, for instance by varying the radius of the solvent or accounting for the type of molecules that interact. A sample code for this calculation is provided in S1 File sample code and the dSASAs are also provided in S1 and S2 Texts using the standard json format, for two representative pdb files. It should be noted that the names of the elements in these files are those found on the protein databank [28] but that comparisons made in later in the paper use the new nomenclature [29].

**Fig 3:**
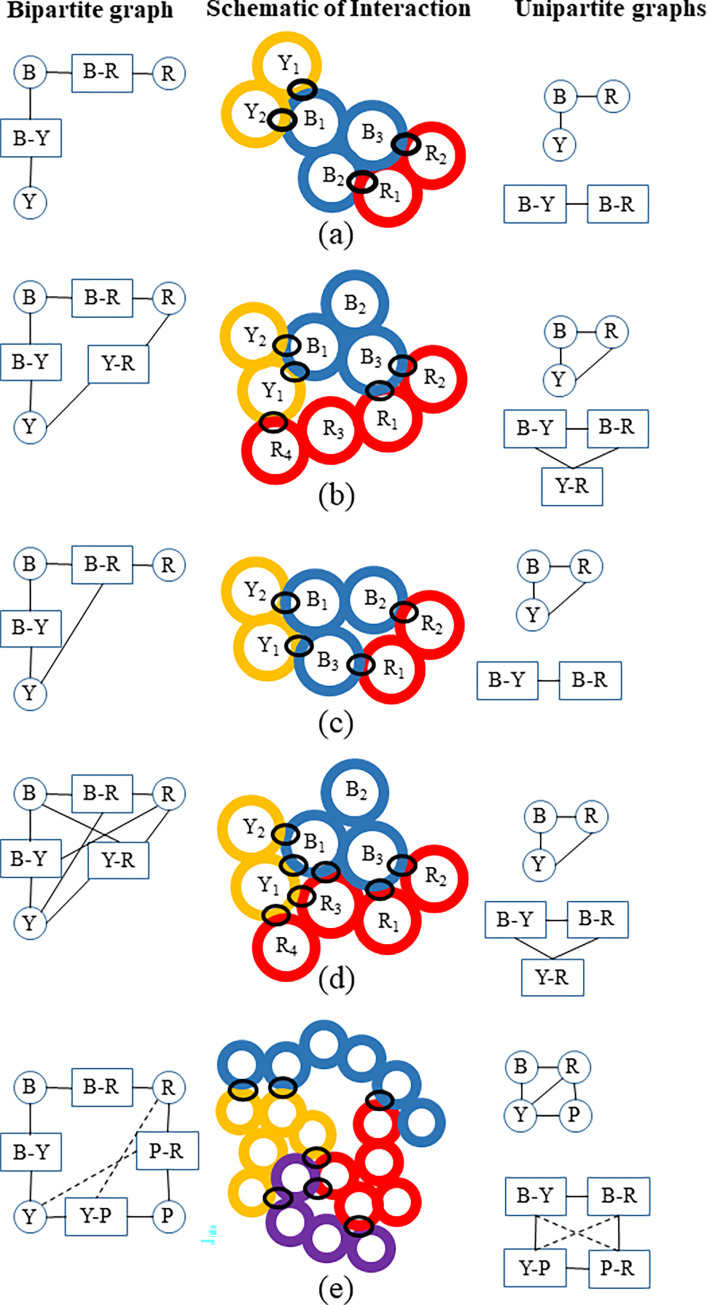
Accounting of interactions in the bipartite graph and their projections. a) and b) Several interactions where the projection from the bipartite graph onto the elements would result in the same interactions in a dyadic graph. Like in Fig 2, the ribosomal elements are depicted by colors (Blue, Yellow, Red and Purple) and the individual circles represent residues. The black ovals indicate interactions. c = Depiction of how the interaction of residue B_3_ with both Y and R leads to an additional interaction between Y and R in the unipartite projection onto the elements that would not be present in the dyadic graph. Additional interactions are captured in the bipartite graph, but may not appear in the unipartite projections, as depicted in (d). (e) Depiction of how triad interactions can lead to significantly more edges in the projection onto the unipartite interaction graph. There are 4 dyadic connections: Blue-Yellow, Blue-Red, Purple-Red and Purple-Yellow. The yellow-purple interaction is justified because there is a single residue that is not present in the purple-red interaction, but the red-yellow interaction is not present because it is part of the yellow-purple interaction. Note that the dashed lines in (e) are meant to indicate additional edges that would not be present if only binary interactions were allowed.

**Fig 4:**
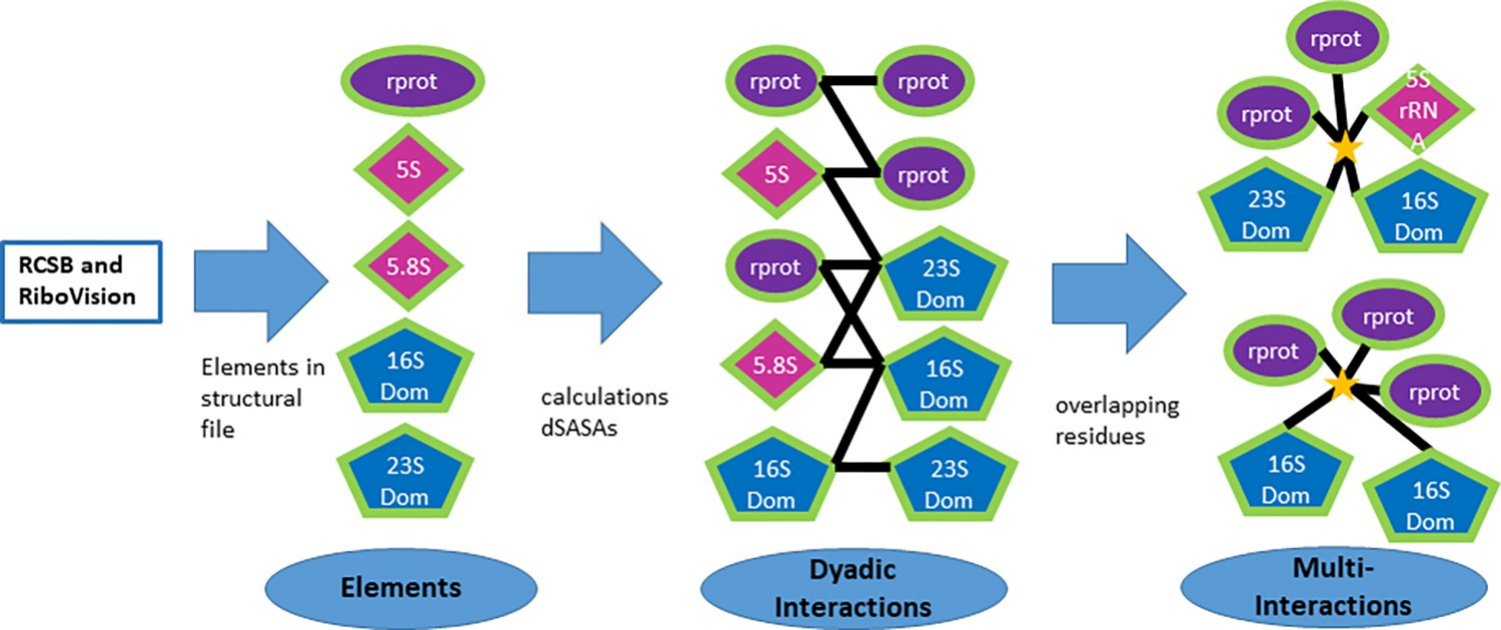
Schematic of the methodology used in this paper. From the information on the RCSB website concerning the pdb structural file, the elements are enumerated by their chains and the 16S and 23S are further divided into domains using the residue numbering found on the Ribovision website [18]. The dyadic interactions are then determined using pymol to calculate the difference between the solvent accessible area of each two elements separately and together. Next the residues in each dyadic interaction are compared with the residues of other dyadic interactions that contain the same elements. If any also include these residues a multiple interaction is said to exist.

Next, it is determined whether each interaction contains further connectivity with other elements. This is done by considering all other dyadic interactions *v*_1x_, *v*_2x_ that involve elements *u*_1_ or *u*_*2*_. It is then determined whether any of the residues from each of these interactions overlap with the *v*_*12*_ interaction. If they do, then a connection to the element *u*_*x*_ is added to the interaction *v*_*12*_. Note that because the interacting residues are identified and not the individual atoms, an interaction surface implicates just the residues involved, and does not guarantee that the same atoms in the residue are involved. Fig 3 depicts some of the possible interconnections and how they are accounted for in the bipartite graph. To reduce redundancy, a dyadic interaction *v*_*13*_ with residues in *u*_1_ that completely overlap those in another interaction *v*_*12*_ is removed from the set of interactions, while a connection, is drawn between *u*_*3*_ and *v*_12_. This is as illustrated in Fig 3E, where the interaction between the yellow and red elements is contained in the connection between the purple-red interaction. A yellow-red interaction is therefore not present, but an edge is drawn between the red protein and the yellow-purple interaction. This typically eliminates just a few interactions involving just one or two residues. An example of the complete bi-adjacency matrices for two files are provided in S2 and S3 Table. An overview of the methodology is summarized in Fig 4.

The overall function of the ribosome can then be analyzed in terms of its connectivity using network theory, where a renaissance of research in the past 20 years provides a large arsenal of techniques [30, 31]. I consider 16 ribosomal bipartite graphs constructed from two representative structural files in the protein databank [28]: 10 from the Bacteria (*T*. *thermophilus*) [16, 32–35] and 6 from Eukarya (3 from *S*. *cerevisiae*, known colloquially as baker’s yeast, and 3 from *H*. *sapiens*) [17, 36]. The ribosomes were chosen because they represent various states of elongation as well as with and without antibiotics and elongation factors, as indicated in S4 Table. I first focus on the basic graph properties that illustrate the similarities in the topological nature of the bacterial and eukaryotic ribosomes to identify the distinguishing features in ribosomal networks. A comparison of unipartite graphs obtained using dyadic connections and those resulting from projections of the bipartite graphs is also considered. Centrality and modularity are then explored, and the results are considered in the context of models of the evolution of the ribosome.

Calculations were carried out using python, Pymol [27], and NetworkX [37]. Table 1 summarizes the basic structural elements of the pdb files and the average basic properties of the resulting graphs. Detailed results for each file are given in S4 Table. The size of the bipartite graph *B* is the number of nodes *n*_*B*_. The number of nodes for each set: n_*u*_ and n_v_ are distinguished. The order *m* is the number of edges. The average degree of the graph, the degrees and the interactions are respectively: *k*_*B*_ = 2*m*/(*n*_*u*_+*n*_*v*_), *k*_*u*_ = *m*/*n*_*u*_, and *k*_*v*_ = *m*/*n*_*v*_. The *average shortest path* length *l* is the average number of nodes to connect two nodes on the graph:

**Table 1:** Average basic properties of the bipartite graphs.

	Bacteria	Eukaryote
**Element size**	66.1	90.5
**Interaction size**	220	381.333
**Order**	1088.7	2165
**Density**	0.075	0.063
**Ave element degree**	16.472	23.9235
**rRNA degree**	39.575	65.359
**rprotein degree**	10.543	16.716
**Average interaction degree**	4.946	5.6765
**Average degree**	7.607	9.1765
**Average path length**	3.479	3.505
**Diameter**	8	8
**Dot clustering**	0.193	0.1715
**Dot clustering element**	0.145	0.1205
**Dot clustering interactions**	0.208	0.184
**rRNA clustering**	0.128	0.1055
**rprotein clustering**	0.15	0.124
**Redundancy**	0.924	0.948
**Element redundancy**	0.828	0.8605
**rRNA redundancy**	0.583	0.6315
**rprotein redundancy**	0.898	0.9085
**Interaction redundancy**	0.952	0.969
**Degree centralization**	0.29	0.2865
**Max degree node**	23S-D2: 10	25/28S-D2: 6
**Betweenness centralization**	6.90E-06	2.80E-06
**Max betweenness node**	23S-D2: 10	25S/28S-D2: 6
**Closeness centralization**	0.384	0.4095
**Max closeness node**		uL2_25S/28S-D4: 5
	uL2_23S-D4: 10	uL2_eL37: 1
**NODF**	0.123	0.116
**Modularity**	0.505	0.527

The values in the table were taken from 10 Bacteria and 6 Eukarya pdb files whose individual values are given in S4A and S4B Table. Note that for centralization the number of times a node appeared for a given element is given.


$$l=\sum_{s,t\in V\;or\;U}d(s,t)/n(n-1)$$ (1)

where *d(s*, *t)* is the number of nodes between elements or interactions *s*, *t*. A common analysis in network theory involves dividing a graph into communities based on a particular characteristic [38]. While there are many ways to do so, here communities are formed using an algorithm that finds the nodes that are more tightly connected to those in their community than to others, known as degree modularity. It is determined using the Clauset-Newman-Moore greedy modularity optimization algorithm [39]. A modularity score for each graph is a measure of how well a graph can be divided into communities and is defined as [40]:

$$Q=\frac{1}{2m}\sum_{\begin{array}{c} ij\\ i\neq j \end{array}}B_{ij}-\frac{k_{i}k_{j}}{2m}$$ (2)

where *B*_*ij*_ is the bi-adjacency matrix of the graph.

A concept that is often explored in bipartite networks is the notion of nestedness [41]. It is particularly important for ecological networks because it distinguishes between ‘generalists’, which interact with many different elements, and ‘specialists’, which interact among a select few set of elements. The measure Nestedness Overlap and Decreasing Fill, NODF [42], is based on the idea that in a perfectly nested network, neighborhoods of nodes with lower degree are included in neighborhoods of larger degree. Consider a pair of nodes *i*, *j* in the same set such that *k*_*i*_ > *k*_*j*_, then in a perfectly nested neighborhood their common neighbors *N*_*ij*_ should be *k*_*j*_. The row and column NODF^R,C^ are respectively:

$${NODF}^{R,C}=\sum_{\begin{array}{c} (i,j)\in R,C\\ j>i,j\neq i \end{array}}\frac{N_{ij}}{k_{j}}$$ (3)

where the rows [elements] and columns [interactions] are from the bi-adjacency matrix. The NODF for the network is then defined as:

$$NODF=\frac{{NODF}^{R}+{NODF}^{c}}{\frac{n_{u}(n_{u}+1)}{2}+\frac{n_{v}(n_{v}+1)}{2}}$$ (4)

To explore the topology of a bipartite network, a plot of the bi-adjacency matrix is plotted such that the set of nodes in the rows and columns are ordered by degree. A perfectly nested network exhibits a clear upper triangular pattern. A modular network will indicate clear blocks of modular sub-networks. Finally, a bipartite network can also have elements of both modularity and nestedness where the different blocks are dominated by a triangular structure.

To measure the importance of different nodes in the network, graph theoretical analysis uses centrality measures [25]. Degree centrality, which measures the number of connections made by a node *u* is:

$$Degree\;centrality(u)=\frac{\#of\;connections\;to\;node\;u}{n-1}$$ (5)

Closeness centrality, which measures how many nodes are necessary to connect to another node in the network is:

$$Closeness\;centrality(u_{1})=\frac{n-1}{\sum_{v}^{n-1}L(u_{1},u_{2})}$$ (6)

where *L*(*u*_1_, *u*_2_) is the shortest distance in terms of number of nodes between node *u*_*1*_ and *u*_*2*_. Finally, betweenness centrality, which measures the positioning of a node on linking paths between other nodes in the network is:

$$Betweenness\;centrality(u)=\sum_{s,t\in U,V}\frac{\sigma_{st}(u)}{\sigma_{st}}$$ (7)

where *σ*_*st*_ is the number of paths linking nodes *s*, *t* that pass node *u*.

These notions can be extended to consider the importance of the single most important hub in the network using the notion of centralization [43]. For a centrality measure *C*, of graph *G* having nodes *u* it is defined as:

$$C(G)=\frac{\sum_{u\in G}\left(max[C\right]-c_{u})}{max(\sum_{i\in G}\left(max[C\right]-c_{u}))}$$ (8)

The centralization allows for a comparison of different graphs, for instance the bipartite graph with its projections [14].

In order to understand the differences between the bipartite and unipartite graphs, the properties of unipartite graphs with dyadic connections are considered. In addition, projection unipartite graphs are created by projecting the bipartite graphs onto the interactions and elements. The latter is achieved by removing one of the sets of nodes and connecting the nodes of the remaining set by merging the edges from the removed set.

Clustering coefficients are meant to capture correlations between neighbors of connected nodes and therefore are vital to understanding graph topology. The definitions introduced in Latapy [15] are used here. Given two nodes *w*_*1*_ and *w*_*2*_, where both nodes belong either to the elements *U* or the interactions *V*, the dot cluster coefficient is:

$${cc}_{•}(w_{1},w_{2})=\frac{\left|N(w_{1})\cap N(w_{2})\right|}{\left|N(w_{1})\cup N(w_{2})\right|}$$ (9)

where *N(w)* are the neighbors of node *w* and represent the correlation of the neighborhoods of the two nodes. The cluster coefficient for a node *w*_1_, which is the average of cc *cc*_•_(*w*_1_, *w*_*x*_) for all *x* in the second nearest neighborhood of *w*_1,_ N(N(*w*_1_)) is:

$${cc}_{•}(w_{1})=\frac{\sum_{w_{x}\in N(N(w_{1}))}{cc}_{•}(w_{1},w_{x})}{\left|N(N(w_{1}))\right|}$$ (10)

As with clustering coefficients in unipartite graphs, the dot cluster coefficient can then be calculated for all the elements and interactions, as well as for the bipartite graph:

$${cc}_{•}(B)=\frac{n_{u}{cc}_{•}(u)+n_{v}{cc}_{•}(V)}{n_{u}+n_{v}}$$ (11)

While the cluster coefficients explore neighborhoods of nodes, the importance of particular nodes can be ascertained using the notion of redundancy. The definition is strictly mathematical. It determines topologically the fraction of pairs of neighbors of a node *u*_1_ that are linked to other nodes:

$$rc(u_{1})=\frac{\left|\left\{\{u_{2},u_{3}\}\subseteq N(u_{1}),\exists u_{1}^{\prime }\neq u_{1},(u_{1}^{\prime },u_{2})\in Eand(u_{1}^{\prime },u_{3})\in E\right\}\right|}{\frac{\left|N(u_{1})|(|N(u_{1})|-1\right)}{2}}$$ (12)

Note that this calculation can only be done if the number of degrees is > 2. The redundancy coefficient of the elemental, interaction and entire graph can be obtained by again averaging over all constituents, including those that are pendant or isolated. This definition makes no assumptions about biophysical or evolutionary aspects of the connectivity.

The bipartite graphs in Fig 1 were generated from the positions found in the structural file. The placement of each element was calculated by averaging the position of the phosphate atoms in the RNA or the α-carbon atoms in the proteins. Each interaction position was found by calculating the average position of all the phosphates or α-carbons in the residues involved in the interaction. The coordinates where then projected onto a two-dimensional surface. Similarly, the same technique is used to draw the projections onto the elements and the interactions.

## 3 Results and discussion

### 3.1 Graph topology

**Fig 5:**
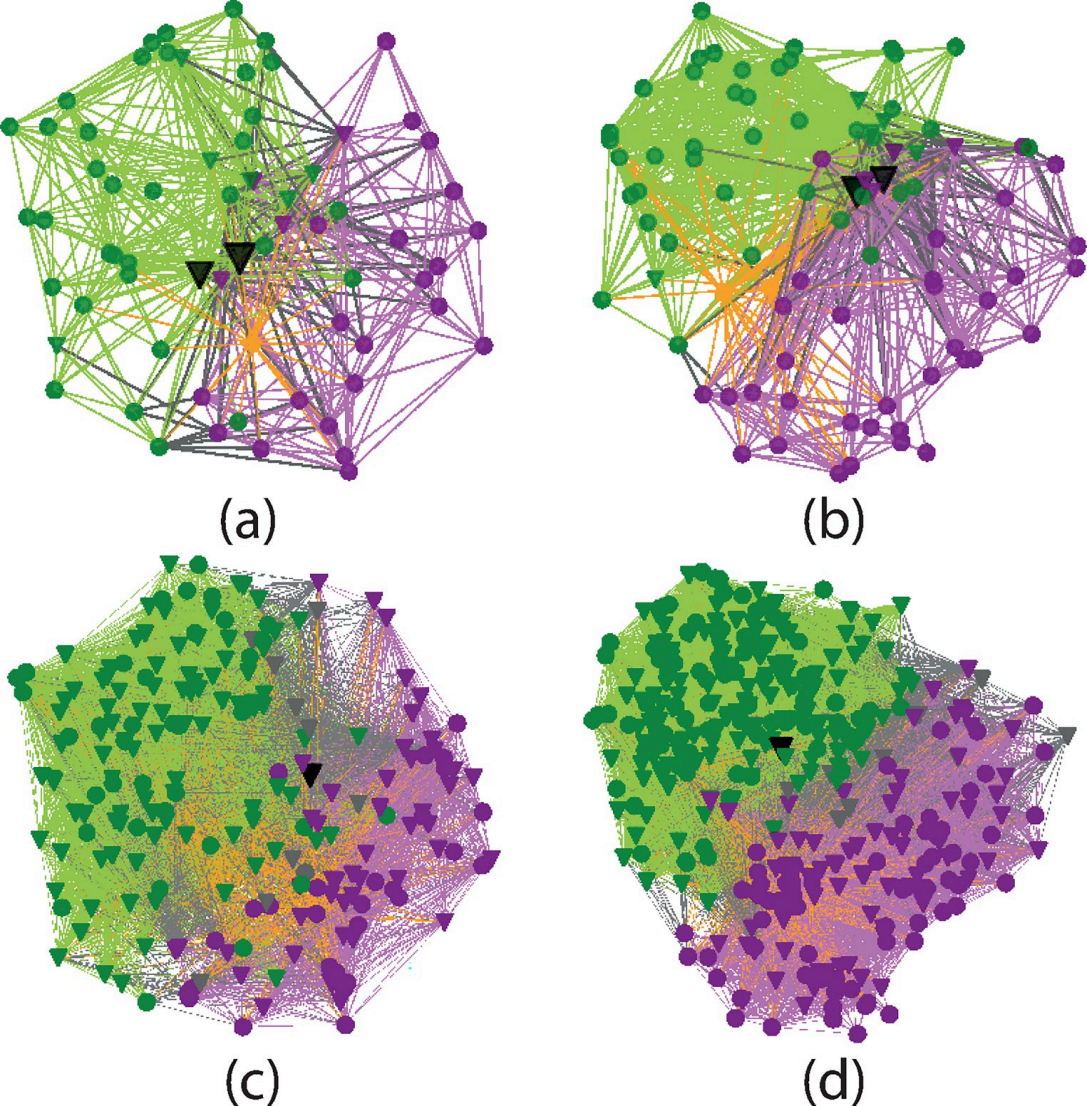
The unipartite projections of the bipartite graphs in Fig 3. (a), (b) are projections of the elements and (c), (d) are projections interactions of respectively *T*. *thermophilus* and *S*. *cerevisiae*. The filled triangles indicate elements and interactions involving rRNAs and the filled circles are all others. The nodes with the largest degrees are indicated in black.

The visual representations of the two networks in Fig 1 show a high density of elemental nodes near the core overlapping region of the subunits and near the perimeter of the ribosome. The interactions are more evenly distributed throughout the structure. Fig 5 shows the projections of each bipartite graph onto the unipartite element and interaction graphs. It is observed that the elements near the subunit interface are predominantly rRNAs (triangles) and those along the edges are rproteins (circles). There is a direct correspondence between this representation and the three-dimensional atomic descriptions and analyses of ribosome structures [44].

The highly conserved nature of the different ribosomal elements across all domains of life has been used to explore the nature of the origins of life [45]. While there is not yet a consensus concerning rooting of the tree of life and its form [46], what seems clear is that Bacteria and Eukarya inherited the ribosome from a common origin [47]. Each then evolved with the eukaryotic ribosome becoming more complex. Understanding the differences in the two networks shown in Fig 1 may thus shed light on the different evolutionary paths of Bacteria and Eukarya and also the ribosome from which they originated.

Table 1 shows how *Eukarya* has 30% more elements, 70% more interactions, and about double the number of interconnections. In addition, there are significantly more rproteins, which comprise approximately 50% by mass and many more rprotein-rprotein interactions [44]. These large compositional changes seem to imply that the network structure should exhibit significant differences in its characteristics. Nevertheless, it was found that many parameters, including the average path length, diameter, redundancy, degree centralization and modularity are remarkably similar. *A priori*, this suggests that the growth mechanisms from the common ancestral origin to the modern bacterial or eukaryotic ribosome were similar. While the methodology here is much too coarse to make any assumptions about what such mechanisms are, one can nevertheless point out some common characteristics.

A distinguishing feature of the ribosome is its large modularity (~ 0.5) and small nesting (NODF ~ 0.12). While the modular groups are discussed in more detail in the next section, it is noted here that they are divided along the lines of the subunits. This modular grouping is a consequence of the rarity of intersubunit bridges. It supports the hypothesis of different evolutions of the subunits, as has been discussed in research focusing on the evolution of the ribosome [48]. As a result, graph properties that depend on calculations involving the distance between different nodes are very similar in the two ribosomes. Specifically, the average path length and the diameter are similar even though the eukaryotic ribosome is more complex.

Another important similarity in the two graphs is the high centralization of the ribosome, which is dominated by the 23S /25S D2. To emphasize this node, it is displayed as the largest marker in black in Figs 1 and 5(A) and 5(B). Models of network growth tend towards the idea that the oldest elements of the network will have the most connections [40]. This is the central idea behind the notion of preferential attachment: the oldest elements make the most connections. It is used to explain the occurrence of scale-free networks [49].

The most ancient part of the ribosome is still a subject of debate. Many researchers believe that the origin of the ribosome is the peptidyl transferase center (PTC), found in 23/25S D5 [50], and shown to exhibit the largest connectivity within the 23S rRNA [51]. The hypothesis that the PTC is the oldest component of the ribosome seems to solve the chicken and egg problem: the possibility to synthesize large proteins must exist to synthesize the ribosome. Other researchers used phylogenetic methods to find that the oldest region was the decoding center (helix 44 in 16S/18S rRNA, corresponding to D3’m) and that the GTPase center in D2 was the oldest region in the 23S rRNA [52]. Recent work has shown that rRNAs might have initially encoded tRNAs and the key protein molecules involved in the ribosome [53], which opens up new possibilities for ribosome evolution. To explore the question of the oldest component of the 23S using graph theory, determination of the interactions in the secondary structure are necessary.

**Fig 6:**
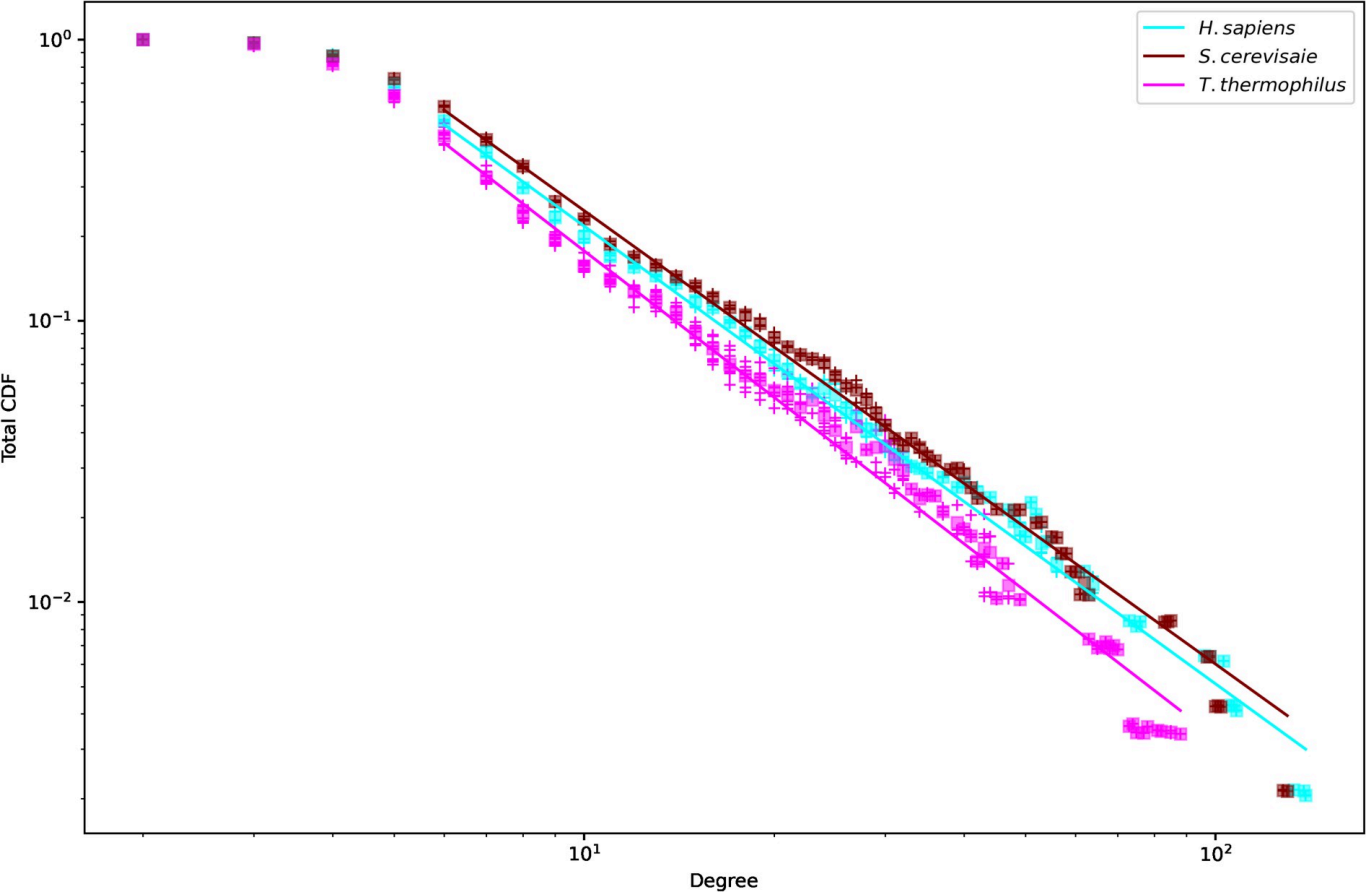
Cumulative density function (CDF) of degrees. The CDF of each individual file is plotted using colored ‘+’ markers. The final aggregate CDF for the three species was obtained by averaging the individual CDF at each degree and is plotted in square markers. The straight line is the fit to a power law of the average CDF. The resulting exponents are given in Table 2.

To explore connectivity in the networks further, Fig 6 plots the node distribution. As seen in Table 1, the largest degree nodes degree in both networks change depending on the state of the ribosome. Fig 6 shows the networks exhibit scale free behavior with exponents between 1.44 and 1.57 (Table 2). Note that the CDF deviates from scale free behavior for very large degrees, which may have to do with the physical limitations of forming further connections. From this analysis it seems that preferential attachment likely played a role in network growth. Further discussion of centrality is found in section 3.3.

**Table 2:** Exponents to the log-log plots in Fig 6.

*T*. *thermophilus*	*S*. *cerevisiae*	*H*. *sapiens*
1.73 ± 0.013	1.61 ± 0.007	1.63 ± 0.007

An important property of the networks is that the ribosomal elements can accommodate many more connections compared to the interactions. This is seen visually in the representations in Fig 5 where there is a large difference in sparsity between the elemental and interaction graphs. In Table 1, there is just a 10% increase in the average interaction degree when comparing Bacteria and Eukarya. This contrasts the 50% increase in the average element degree. To grow in complexity, the ribosome is more efficient in adding to its machinery and functionality by incorporating either new elements or new parts of elements (segment expansions for RNAs and new extensions for rproteins) as opposed to increasing the size of interactions. This mathematical characteristic is also perfectly consistent with models of ribosome evolution [54–56]. Smaller ancient RNAs and proteins are hypothesized to have gradually added substructures to form modern rRNAs and proteins. The different role played by the rRNAs and rproteins is an important distinguishing characteristic. The data in Table 1 shows that while the rRNAs make nearly four times more interactions compared to rproteins, their clustering coefficients are 15% smaller. The larger cluster coefficients in rproteins means that they have greater correlation with their neighbors compared to the rRNA domains. The clustering coefficient is smaller in Eukarya compared to Bacteria, suggesting again that making many connections is difficult when the number of elements is increased, consistent with recent research on phylogenetic accretion [56].

Another striking result is that the topological redundancy of rproteins is greater than 90% in both domains of life. Unlike the cluster coefficient, the redundancy increases with more elements. The higher cluster coefficient coupled with the higher redundancy of rproteins suggests that nature’s solution to the interaction bottleneck may have been to use rproteins to provide ‘short cuts’ (perhaps via allosteric pathways) through the ribosome. To further explore such possibilities, though, investigations as to the biochemical pathways enabled by the interactions need to be determined. To do so, it is necessary to explore the nature of the residues involved in the interactions. For instance, one could consider hydrophobic interactions and consider whether they can act as flexible connectors between elements with the possibility of allowing a cascade of dynamic motion, a method that has proven quite effective in understanding the allostery in protein kinases [57]. One might also explore the possible role of charge transfer by considering regions of connectivity and elements where this could occur [23].

This remarkably large mathematical redundancy is intriguing. First, it should be noted that this result does not imply there is a biophysical redundancy. What appears to be an equivalent connected path mathematically is likely to contain very different biochemical properties. They may also have evolved in different manners. Nevertheless, a topological redundancy suggests that alternative paths may enable the ribosome to be multi-functional. It could also permit enhanced resiliency, in the mathematical sense [58] where the absence or presence of different ribosomal elements does not impede ribosome function significantly.

Table 3 compares the average basic properties of the three types of unipartite graphs for the different structural files and the data for each the individual structural files are given in S5A and S5B Table. The unipartite projection onto the elements has approximately double the number of connections in Bacteria and nearly three times as many in Eukarya compared to their dyadic counterparts. Consequently, the dyadic graphs appear highly dis-assortative, while the elemental and interaction graphs are not. In other words, in a unipartite representation, the dis-assortative nature may be due rRNA domains forming many more connections than the rproteins, but from the vantage of a bipartite network, we see that the formation of larger interaction surfaces involving both rRNA domains and rproteins makes the interconnectivity between the elements significantly more uniform. For the elemental graphs, this is a result of the three times larger increase in the degrees of rproteins compared to just two times in rRNAs. For the interaction graphs, it is once again the fact that the number of interactions is limited, causing a very large number of connections in the projection. By not considering multiple interactions, the rproteins appear to make many fewer connections and to play a much less important role in the connectivity.

**Table 3:** Average basic graph properties of the three types of unipartite graphs for Bacteria and Eukarya.

	Bacteria			Eukarya		
	dyadic	element	interaction	dyadic	element	interaction
**Size**	66.3	66.3	220	90.5	90.5	381
**Order**	223.5	501.5	9035	388.5	1016	26295
**Average degree**	3.371	7.565	41.004	4.295	11.23	68.9
**Average rRNA degree**	13.35	26.683	0	18.949	39.859	0
**Average rprotein degree**	4.967	11.843	0	6.733	19.167	0
**Max degree**	25.4	42.5	160.9	35.167	63	285.8
**Diameter**	5.1	4	3.2	5.8	3.1665	4
**Average path length**	2.52	1.937	1.683	2.58	1.91	1.71
**Cluster coefficient**	0.455	0.694	0.757	0.487	0.705	0.781
**rRNA cluster coefficient**	0.201	0.454	0	0.224	0.483	0
**rprotein cluster coefficient**	0.519	0.758	0	0.533	0.75	0
**Density**	0.103	0.232	0.374	0.096	0.251	0.363
**Assortativity**	-0.276	-0.116	0.28	-0.237	-0.036	0.313
**Degree centralization**	0.294	0.432	0.364	0.304	0.464	0.392
**Max degree node**		23S-D2: 9				uL2_28S-D4: 5
	23S-D2	23S-D5: 1	uL2_23S-D4	28S-D2	28S-D2	uL2_eL43: 1
**Between centralization**	1.25E-04	1.25E-04	1.25E-04	6.13E-05	6.13E-05	6.13E-05
**Max between node**			uS13_uS19: 7			uL2_28S-D4: 1
	23S-D2	23S-D2	uL2_23S-D4: 3	28S-D2	28S-D2	uS19_uS13: 5
**Closeness Centralization**	0.341	0.441	0.377	0.338	0.482	0.4115
**Max closeness node**		23S-D2: 9				uL2_28S-D4: 5
	23S-D2	23S-D5: 1	uL2_23S-D4	28S-D2	28S-D2	uS19_uS13: 1
**Modularity**	0.422	0.354	0.288	0.432	0.355	0.324

Note that the numbers reported are averages of the graphs in S5A and S5B Table. For the centralization, all of the nodes that appear are reported.

The cluster coefficients are also much larger in the projected graphs. However, the rRNA is much larger in the elemental graphs compared to the dyadic graphs, suggesting that dyadic graphs will short change the role of the rRNAs in the connectivity: the bipartite network analysis confirms their important role in the connectivity of the network as we know must be the case from explorations of evolution [48, 50, 59]. A final observation is that the cluster coefficient of the rproteins is approximately constant in Bacteria and Eukarya. Although numerous researchers have remarked that the eukaryotic ribosome exhibits increased connectivity of rproteins [44, 60], in the context of network theory, the change in connectivity is a natural consequence of a greater number of nodes and similar underlying growth mechanisms. Overall, the bipartite representation thus reveals a much larger connectivity than the unipartite networks. Small changes that occur spontaneously on the ribosome without changes in elements thus can be much more efficiently detected and explored.

In this sub-section some of the basic features of the ribosomal bipartite graphs were investigated. It was found that the highly modular nature of the ribosome has a strong impact on network measures. Another distinguishing characteristic is that the interactions are constrained in size, confirming the importance of accretion for increased functionality. Despite the importance of constraints, preferential attachment seems to be important for explaining the network connectivity. One remarkable feature is highly topological redundancy, which when considered with biophysical or evolutionary studies, may help explain ribosome resiliency and multi-functionality. Another important observation here is just how similar the graph properties of Bacteria and Eukarya are, despite the significant increase in elements in the latter. Finally, a comparison with the unipartite graphs shows the utility of the bipartite representation.

### 3.2 Modularity

**Fig 7:**
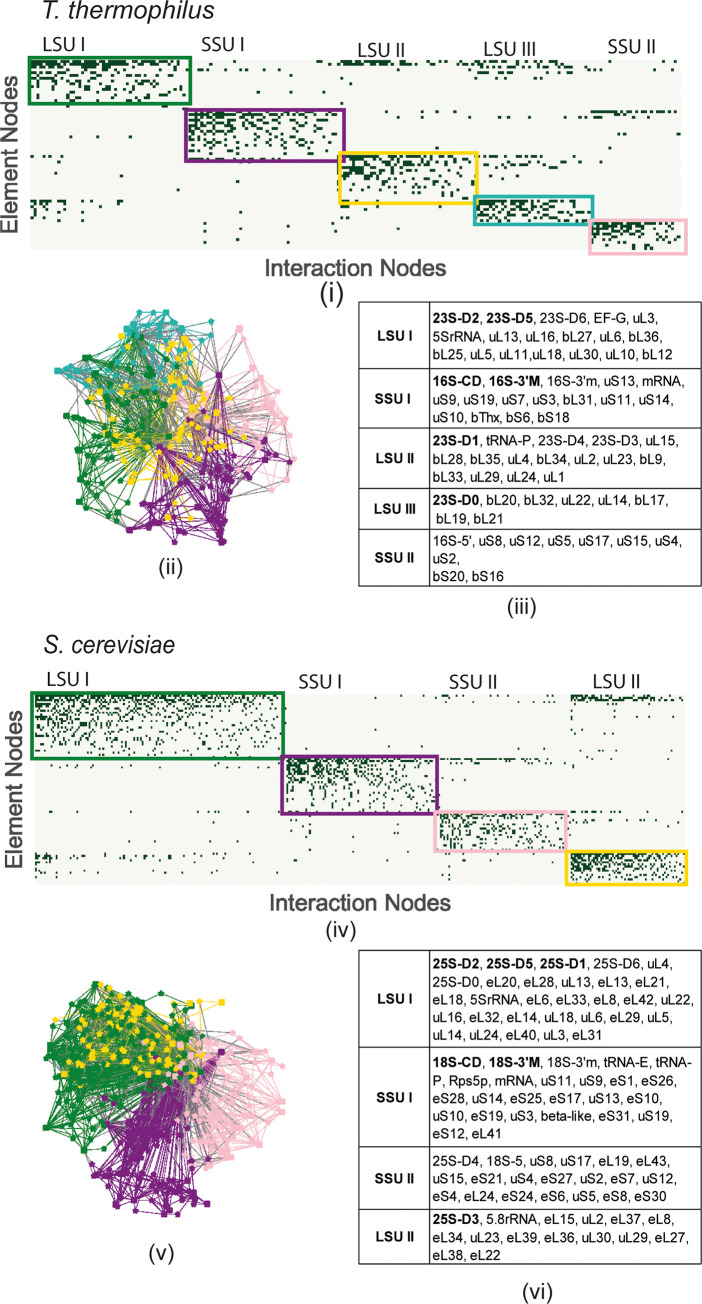
Modularity of (a) *T*. *thermophilus* and (b) *S*. *cerevisiae* (i) The bi-adjacency matrix, the rectangular boxes correspond to the modular groups in the graph in (ii) and the element nodes are listed in (iii).

To explore the modularity of the networks and the relation with centrality, we restrict the discussion to just a single file from *T*. *thermophilus* and *S*. *cerevisiae*. Fig 7 illustrates the important modularity for both the bacterial (a) and eukaryotic (b) ribosomes, determined using equation [2]. Fig 7I, plots the connectivity between elemental and interaction nodes, the bi-adjacency matrix, and colored rectangles delimit the modular groups. The first important observation is that the ribosome structure is not nested, as the upper triangular pattern is not observed. This result is true for all the pdb structures investigated. The modular groups connect with each other but there is not a hierarchical order. To show that the modules divide along the lines of the two subunits, the elements and connections in each colored box in (i) are drawn in this color in the 2D graphs in (ii). The elements of each modular subgroup are listed in (iii) in order of the number of connections (highest to lowest). Note that the elements in bold correspond to the large degree centrality, discussed in greater detail in the next section. In this particular example, the largest module in each subunit contains the elements thought to be most ancient universal components [59]: the LSU I (D5 and D2 of 23S/25S rRNA) or in SSU I (D3’m and CD of 16S/18S). Nevertheless, this observation would need to be confirmed by considering the connectivity in the specific helices in these regions. These results support the idea of separate evolutions of the subunits, which could explain the large modular connectivity.

As we have observed in previous research, [4] the modular groups change as a function of the different states. Typically, 23S-D2 and D5 are found together in the same module but 23S-D0 is found in a second LSU domain. Here we observe that the largest modular groups of *T*. *thermophilus* and *S*. *cerevisiae* in each subunit are similar, most likely because the two structural files are in the post-elongation state. The elements in the other modules can vary in significant ways, hinting at the important functional differences known to exist [44]. Differences in the LSU modules between the two species may be due to increased functionality necessary for different types of proteins that traverse the peptide exit tunnel. Some elements change modules, for instance uL4 appears to play different roles in the two domains of life. In Bacteria it is found in the LSU II and has fewer connections compared to Eukarya, where it is found in the LSUI. 23/25S D4 is present in the LSU II group in Bacteria but in the SSUII group in Eukarya. In fact, the SSU II modular group in *S*. *cerevisiae* has a significant number of elements from the LSU, demonstrating mathematically the increased importance of intersubunit bridges in Eukarya, as was observed in [60]. Another observation is that there are fewer modules in Eukarya, and they are necessarily larger. It would be interesting to compare the modularity at different stages of elongation in the two ribosomes to see whether this is a particularity of the post-peptide bond state or a generic feature of the two ribosomes.

The modular decomposition shows that the ribosome is predominantly divided along the two subunits. The largest modules in each subunit reflects what others have found to be the most highly conserved portions of the ribosome [61] and the most ancient parts, regardless of the model used. Further work would be needed to explore whether these conserved portions are involved in the interactions or not however, to gauge their importance for the graphical analysis. The modules are relatively independent, meaning that they do not exhibit a hierarchical structure relative to each other (small NODF). Further work could explore how the modularity changes with ribosomal state to gain insights into the multi-functionality of the different elements in a module. In addition, it would be interesting to investigate whether there is co-evolution of elements within a module.

### 3.3 Centrality

Centrality measures explore the role of important ribosomal elements in the network. In S6A and S6B Table the top 10 centrality nodes for each of the structural files is reported. To place these results in context, Table 4 reports the average centrality of the top 5 centrality elements found in each of the files. Some elements such as tRNAs and the Elongation factors are only found in some of the files. Nevertheless, reporting the average value provides an idea of the overall importance.

**Table 4:** Ave top centrality values.

Bacteria					
Degree		Betweenness		Closeness	
Node	Value	Node	Value	Node	Value
23S-D2	0.28	23S-D2	0.297	23S-D2	0.412
23S-D5	0.234	23S-D5	0.172	uL2_23S-D4	0.38
23S-D1	0.155	16S-CD	0.139	23S-D5	0.379
23S-D0	0.151	16S-3’M	0.097	uS13_16S-3’M	0.366
16S-CD	0.147	EF-G	0.082	uL3_23S-D5	0.365
16S-3’M	0.135	23S-D4	0.076	uL6_23S-D6	0.359
		tRNA-P	0.075	EF-G_uL6	0.358
		tRNA-pe*/E	0.069	uS13_16S-3’M	0.356
		tRNA-E	0.068	16S-CD	0.355
				uL15_23S-D5	0.353
				23S-D4	0.352
				bL35_23S-D5	0.352
				bL27_23S-D5	0.35
				u13_23S-D2	0.347
***S*. *cerevisiae***					
**Degree**		**Betweenness**		**Closeness**	
**Node**	**Value**	**Node**	**Value**	**Node**	**Value**
25S-D2	0.275	25S-D2	0.299	25S-D2	0.42
25S-D5	0.216	18S-CD	0.165	uL2_25S-D4	0.384
25S-D1	0.208	25S-D5	0.125	uL2_L43-A	0.382
18S-CD	0.179	18S-3’M	0.12	eL19_25S-D4	0.375
25S-D3	0.132	25S-D4	0.099	25S-D5	0.367
18S-3’M	0.129			uS17_18S-5’	0.366
***H*. *sapiens***					
**Degree**		**Betweenness**		**Closeness**	
**Node**	**Value**	**Node**	**Value**	**Node**	**Value**
28S-D2	0.289	28S-D2	0.313	28S-D2	0.412
28S-D1	0.226	18S-CD	0.167	uL2_28S-D4	0.385
28S-D5	0.209	28S-D5	0.13	uL2_L37a	0.384
18S-CD	0.158	18S-3’M	0.119	eL19_28S-D4	0.381
28S-D3	0.135	28S-D1	0.098	uS17_18S-5’	0.369
18S-3’M	0.128	28S-D4	0.094	28S-D5	0.364
				uL2_28S-D5	0.361

Note that interactions are reported using the notation elem1_elem2.

The degree centrality may be the most interesting measure to explore insights into the evolution from a universal ribosome. The scaling law observed in Fig 6 and the notion that older nodes should have greater connectivity [48], is directly relevant for the nodes exhibiting the largest degree centrality. Nevertheless, there are many reasons why the oldest nodes might not be the most connected. First, it depends on the mechanism of ribosome growth. If growth is random, then each of the nodes should have on average the same number of connections. If growth started from a central core and then evolved to optimize different objectives, the starting central core would appear more connected relative to other nodes not in the central core if growth is not explosive.

Concerning the degree centrality in the LSU, we see that 23S-D2, which is involved in intersubunit bridges and the dynamic motion of the ribosome and contains the A, P and E sites, is by far the most important node, with an average score exceeding that of the next most important node (23S-D5) by ~ 40%. In comparison, we see that while the homologous 25/28S D5 is the second most important node in Bacteria and *S*. *cerevisiae*, its advantage over the third most important node has significantly narrowed and indeed in *H*. *sapiens* it is the third most important node. Strikingly, the decrease in importance of 25/28S D5 correlates with an increase in importance of 25/28S D1. The latter plays an important role in the nascent protein exit tunnel and its increasing importance reflects its more important role in Eukarya, which is capable of synthesizing much more complex proteins. Similarly, we see that D0, found to be one of the 4^th^ most important nodes in Bacteria, is replaced by D3 (in position 5), which also plays an important function in the exit tunnel. These results suggest that D2 and D0 played an important role in the common core of a universal ribosome and that as more connections and elements are added their role relative the total functionality of the ribosome diminished.

An earlier paper explored connectivity within the 23S and found the number of connections (in parenthesis) from highest to lowest was found to be: D5 (36), D4 (32), D2 (31), D1 (17), D6 (14), D3 (8) [51]. The results presented here do not consider intraconnectivity, which may account for the discrepancy, or our method allows for many more connections to be observed. Investigations of ribosome evolution using A-minor interactions posit that the oldest parts of the ribosome are D5, followed by D0 [20]. Research exploring the number of conserved residues in the different domains of 23S also found that D5 has the most conserved residues, followed by D4 and then D2 [61]. To explore this further, investigations of the network need to consider the connectivity of the individual helices to explore the degree centrality of their interactions.

The domain in the SSU with the largest degree, the central domain (CD), has an even larger value in Eukarya than in Bacteria. The CD contains the central pseudo knot, which provides and important structural core for the small subunit. The other domain in the top 5 is the 3’M domain which contains P-site, both of which realize essential connections for ribosome function. Its score is just slightly smaller in Eukarya. The h1, h2 in the 5’ domain, h28 in the 3’M domain, and part of h44 and h45 in the 3’m domain are thought to be the oldest parts in the accretion model [59] and in the phylogenic model h44, h11 (5’ domain) and h34 (3’M domain).

No rproteins appear in the top 5-degree centrality hubs. Those that are the most fundamental during assembly are hypothesized to be the most ancient, namely, uL2, uL3 and uL4 [48]. Nevertheless, other analyses of the evolution of the ribosome rRNA have used A-minor interactions [46, 50] and phylogenic analysis [52]. A recent extension of the accretion model of ribosome evolution proposed that the correlation between the interactions between rproteins and rRNAs could be used to determine the age of the rproteins. The oldest rproteins where thus hypothesized to be: uL2, uL3, uL15, uL4, uL13, uL14, uL16 [62]. The rprotein with the highest degree centrality in Bacteria is uL3, which has an average score of 0.081, followed by uL15, with an average score of 0.072, with 24 connections. For both yeast and human, the rprotein with the highest degree is eL15 [score of 0.089 and 0.09 respectively], followed by uL4 for yeast (score 0.087) and uL2 for human (score 0.071). It should be noted that in yeast the uL2 rprotein has a similar score as in human (0.073). It is thus quite striking that the most dominant rproteins are not the same in the different domains and even the different species. This result is very consistent with the hypothesis that rproteins were an addition to an already relatively developed rRNA dominated ribosome.

Degree connectivity is now considered in the context of the modularity from Sec. 3.2. Fig 7(iii) lists the elements of the modules from highest to lowest degrees. One can observe that 23S D2 and 23S D5, as well as uL3 are all found in the LSUI module in Bacteria. Likewise, it is observed that 16S (18S) -CD is in the SSUI module containing the decoding center (16/18S 3’m) and 16(18)S 3’M. In both the small and large subunit, the largest connected nodes are thus in the largest module which are relatively similar in the two domains. It could be that the ribosome evolved additional regions of high connectivity because the functional regions were saturated and could not develop additional connections. This could explain why the intra-domain connectivity of 23/25/28S D5 is larger than 23/25/28S D2 [51].

Betweenness centrality measures how much a particular node is on the link between paths connecting two other nodes. 23/25S/28S D2 of is also the dominant node here. While 23S-D5 is the second most important in Bacteria, like in the degree centrality, its score significantly reduced in Eukarya, but in favor of a different element, 18S-CD. In Bacteria we observe that EF-G appears in the betweenness centrality, emphasizing its importance. We also find tRNA-P, the hybrid state and tRNA-E. The first rproteins to appear in the betweenness centrality are involved in the first interaction to appear: uS13_uS19 (score 0.048), followed by uL2_23S-D4 (score 0.038).

Closeness centrality is a measure of how close a node is to connecting to all other nodes in the network. Strikingly, this is the only centrality measure where interactions appear in the top five and where rproteins appear. This result is consistent with the much higher redundancy of rproteins that was observed in Section 3.1 and supports for the idea that rproteins may allow mathematical short cuts through the ribosome. The dominant interaction in both ribosomes is the same: interaction between 23 (25)S D4 and the rprotein uL2, neither of which appear alone in the top centrality hubs. Both uL2 and 23/25/28S D4 play an important role in connecting the two subunits via their inter-subunit bridges. Not surprisingly, this interaction appears as the dominant one for degree centralization in both graphs in the projection onto interactions (Table 3). It is striking nevertheless that uL2, which is one of the assembly proteins in Bacteria and thought to be one of the oldest, appears in the most important interaction. Another important observation is again the reduced importance of 23/25/28S-D5 in Eukarya as compared to Bacteria. This observation is consistent with structural analysis of the Eukarya [44, 60]. Nevertheless, the significant differences in the number of connections of the rproteins is likely to be strongly influenced by the very different assembly in Eukarya compared to Bacteria [63].

The analysis has considered generalized regions, not the specific structures, that are hypothesized to be the oldest parts of the ribosome. A more careful analysis might explore the specific connectivity of the different hypothesized oldest regions and whether they include conserved residues. Nevertheless, the analysis, along with that of previous work [4] does allow some relatively broad observations. First, the evolution from the universal ribosome appears to be constrained in the development of new interactions. It is also observed that the more highly connected Eukarya network exhibited a decrease in the centrality scores for 23/25/28S D5. This is likely due to specialization: with increasing complexity of the ribosome new regions develop more functionality, reducing the relative importance of older ribosomal components relative to the entire ribosome. It is therefore not inconsistent with this mathematical analysis that 23/25/28S D5 could be the oldest component of the ribosome.

## 4 Conclusion

This research introduced a mathematical representation for modeling ribosome structure that can succinctly capture many characteristics. The similarity of the graph properties indicates that the growth mechanisms of Eukarya and Bacteria starting from a common ribosome were similar. The scale free behavior of the CDF of nodes suggests that preferential attachment is at least in part played a role in network growth. It was found that the properties of the ribosome were strongly influenced by two topological properties: 1) a limitation of the size of the interaction surfaces available to extend the connectivity of the network, 2) the important functional division between communities in the LSU and SSU. The importance of 23/25/28S D5, which decreases in more complex networks, suggests that this domain may be one of the oldest. Further, the importance of different rproteins in the two different domains and two species supports the hypothesis that they were added after an rRNA core already existed.

The limitations on the size of the interaction surfaces, and the physical constraints of the ribosomal environment suggest that connectivity may be the route by which the ribosome is able to increase functionality. One of the most striking features of the analysis is the very large redundancies that are present in the networks, which may provide a key to understanding how the ribosome can function despite changes in composition and connectivity. The presence of this large mathematical redundancy should also inspire new types of architectures for realization of more robust man-made networks, for instance in technological and electronic architectures, that could significantly reduce network failure.

Using interactivity as a means for mathematically modeling complex biomolecules is a promising technique for further explorations. Such explorations could consider modifying the definitions of the elements to provide finer details, such as considering the secondary structure of the rRNAs or the rproteins. More importantly, though, the mathematical representation used in the modeling here does not take into account the biochemical underpinnings of the interactions. Combining the interactivity with the biochemical nature of the interactions is likely the most important extension of this work and would allow a deeper understanding of both the allostery present during ribosome function and the ribosome evolution.

## Supporting information

S1 File Sample code for calculating interactions using pymol. (TXT)

S1 Text dSASA of the 5OT7 (*T*. *thermophilus*) file in json format. The information for each interaction is provided for each interacting residue in the following format (partner_name, residue_number, dSASA).
(JSON)

S2 Text dSASA of the 6t7i (*S*. *cerevisiae*) file in json format. The information for each interaction is provided for each interacting residue in the following format (partner_name, residue_number, dSASA)
(JSON)

S1 Table List of elements in the two pdb files considered in this paper. 5OT7 corresponds to *T*. *thermophilus* and 6t7i corresponds to *S*. *cerevisiae*.
(PDF)

S2 Table Bi-adjacency matrix for pdb file 5ot7 (*T*. *thermophilus*). (PDF)

S3 Table Bi-adjacency matrix for pdb file 6t7i (*S*. *cerevisiae*). (PDF)

S4 Table Individual basic graph properties for (a) *T*. *thermophilus* pdb files and (b) *S*. *cerevisiae* and *H*. *sapiens* pdb files.
(PDF)

S5 Table Individual basic graph properties of unipartite graphs for (a) *T*. *thermophilus* pdb files and (b) *S*. *cerevisiae* and *H*. *sapiens* pdb files.
(PDF)

S6 Table Individual top 10 centrality scores for (a)*T*. *thermophilus* pdb files and (b) *S*. *cerevisiae* and *H*. *sapiens* pdb files.
(PDF)

## References

[1] Rodnina MV. Translation in Prokaryotes. Cold Spring Harb Perspect Biol. 9 janv 2018;10(9):a032664. doi: 10.1101/cshperspect.a032664 29661790 PMC6120702

[2] Ban N, Nissen P, Hansen J, Moore PB, Steitz TA. The Complete Atomic Structure of the Large Ribosomal Subunit at 2.4 Å Resolution. Science. 11 août 2000;289(5481):905–20. 10937989 10.1126/science.289.5481.905

[3] Wimberly BT, Brodersen DE, Clemons WM, Morgan-Warren RJ, Carter AP, Vonrhein C, et al. Structure of the 30S ribosomal subunit. Nature. sept 2000;407(6802):327–39. doi: 10.1038/35030006 11014182

[4] Calvet LE, Matviienko S, Ducluzaux P. Network theory of the bacterial ribosome. PLOS ONE. 5 oct 2020;15(10):e0239700. doi: 10.1371/journal.pone.0239700 33017414 PMC7535068

[5] David-Eden H, Mandel-Gutfreund Y. Revealing unique properties of the ribosome using a network based analysis. Nucleic Acids Res. août 2008;36(14):4641–52. doi: 10.1093/nar/gkn433 18625614 PMC2504294

[6] Kürkçüoğlu Ö. Exploring allosteric communication in multiple states of the bacterial ribosome using residue network analysis. Turk J Biol. 25 oct 2018;42(5):392–404. doi: 10.3906/biy-1802-77 30930623 PMC6438126

[7] Kurkcuoglu O, Doruker P, Sen TZ, Kloczkowski A, Jernigan RL. The ribosome structure controls and directs mRNA entry, translocation and exit dynamics. Phys Biol. 24 nov 2008;5(4):046005. doi: 10.1088/1478-3975/5/4/046005 19029596 PMC2907240

[8] Zimmermann MT, Jia K, Jernigan RL. Ribosome Mechanics Informs about Mechanism. J Mol Biol. 27 févr 2016;428(5, Part A):802–10. doi: 10.1016/j.jmb.2015.12.003 26687034 PMC4789072

[9] Tama F, Valle M, Frank J, Brooks CL. Dynamic reorganization of the functionally active ribosome explored by normal mode analysis and cryo-electron microscopy. Proc Natl Acad Sci. 5 août 2003;100(16):9319–23. doi: 10.1073/pnas.1632476100 12878726 PMC170916

[10] Wang Y, Rader AJ, Bahar I, Jernigan RL. Global ribosome motions revealed with elastic network model. J Struct Biol. 1 sept 2004;147(3):302–14. doi: 10.1016/j.jsb.2004.01.005 15450299

[11] Sanbonmatsu KY, Joseph S. Understanding discrimination by the ribosome: stability testing and groove measurement of codon-anticodon pairs. J Mol Biol. 18 avr 2003;328(1):33–47. doi: 10.1016/s0022-2836(03)00236-5 12683995

[12] Sanbonmatsu KY, Joseph S, Tung CS. Simulating movement of tRNA into the ribosome during decoding. Proc Natl Acad Sci U S A. 1 nov 2005;102(44):15854–9. doi: 10.1073/pnas.0503456102 16249344 PMC1266076

[13] Whitford PC, Blanchard SC, Cate JHD, Sanbonmatsu KY. Connecting the Kinetics and Energy Landscape of tRNA Translocation on the Ribosome. Chen SJ, éditeur. PLoS Comput Biol. 21 mars 2013;9(3):e1003003. doi: 10.1371/journal.pcbi.1003003 23555233 PMC3605090

[14] Pavlopoulos GA, Kontou PI, Pavlopoulou A, Bouyioukos C, Markou E, Bagos PG. Bipartite graphs in systems biology and medicine: a survey of methods and applications. GigaScience (Internet). 1 avr 2018 (cité 22 oct 2020);7(4). Disponible sur: https://academic.oup.com/gigascience/article/7/4/giy014/4875933 doi: 10.1093/gigascience/giy014 29648623 PMC6333914

[15] Latapy M, Magnien C, Vecchio ND. Basic notions for the analysis of large two-mode networks. Soc Netw. 1 janv 2008;30(1):31–48.

[16] Macé K, Giudice E, Chat S, Gillet R. The structure of an elongation factor G-ribosome complex captured in the absence of inhibitors. Nucleic Acids Res. 06 2018;46(6):3211–7. doi: 10.1093/nar/gky081 29408956 PMC5887593

[17] Tesina P, Lessen LN, Buschauer R, Cheng J, Wu CCC, Berninghausen O, et al. Molecular mechanism of translational stalling by inhibitory codon combinations and poly(A) tracts. EMBO J. 03 2020;39(3):e103365. doi: 10.15252/embj.2019103365 31858614 PMC6996574

[18] Bernier CR, Petrov AS, Waterbury CC, Jett J, Li F, Freil LE, et al. RiboVision suite for visualization and analysis of ribosomes. Faraday Discuss. 23 oct 2014;169(0):195–207. doi: 10.1039/c3fd00126a 25340471

[19] Gan HH, Pasquali S, Schlick T. Exploring the repertoire of RNA secondary motifs using graph theory; implications for RNA design. Nucleic Acids Res. 1 juin 2003;31(11):2926–43. doi: 10.1093/nar/gkg365 12771219 PMC156709

[20] Petrov AS, Bernier CR, Hsiao C, Norris AM, Kovacs NA, Waterbury CC, et al. Evolution of the ribosome at atomic resolution. Proc Natl Acad Sci. 15 juill 2014;111(28):10251–6. doi: 10.1073/pnas.1407205111 24982194 PMC4104869

[21] Petrov AS, Gulen B, Norris AM, Kovacs NA, Bernier CR, Lanier KA, et al. History of the ribosome and the origin of translation. Proc Natl Acad Sci. 15 déc 2015;112(50):15396–401. doi: 10.1073/pnas.1509761112 26621738 PMC4687566

[22] Timsit Y, Sergeant-Perthuis G, Bennequin D. Evolution of ribosomal protein network architectures. Sci Rep. 12 janv 2021;11(1):625. doi: 10.1038/s41598-020-80194-4 33436806 PMC7804294

[23] Sosorev A, Kharlanov O. Organic nanoelectronics inside us: charge transport and localization in RNA could orchestrate ribosome operation. Phys Chem Chem Phys (Internet). 2021 (cité 20 janv 2021); Disponible sur: https://pubs.rsc.org/en/content/articlelanding/2021/cp/d0cp04970k doi: 10.1039/d0cp04970k 33448272

[24] Paci M, Fox GE. Major centers of motion in the large ribosomal RNAs. Nucleic Acids Res. 19 mai 2015;43(9):4640–9. doi: 10.1093/nar/gkv289 25870411 PMC4482067

[25] Sobolev V, Sorokine A, Prilusky J, Abola EE, Edelman M. Automated analysis of interatomic contacts in proteins. Bioinforma Oxf Engl. avr 1999;15(4):327–32. doi: 10.1093/bioinformatics/15.4.327 10320401

[26] Shrake A, Rupley JA. Environment and exposure to solvent of protein atoms. Lysozyme and insulin. J Mol Biol. 15 sept 1973;79(2):351–71. doi: 10.1016/0022-2836(73)90011-9 4760134

[27] The PyMOL Molecular Graphics System, Version 2.0 Schrödinger, LLC.

[28] Berman HM, Westbrook J, Feng Z, Gilliland G, Bhat TN, Weissig H, et al. The Protein Data Bank. Nucleic Acids Res. 1 janv 2000;28(1):235–42. doi: 10.1093/nar/28.1.235 10592235 PMC102472

[29] Ban N, Beckmann R, Cate JHD, Dinman JD, Dragon F, Ellis SR, et al. A new system for naming ribosomal proteins. Curr Opin Struct Biol. févr 2014;24:165–9. doi: 10.1016/j.sbi.2014.01.002 24524803 PMC4358319

[30] Albert R, Barabási AL. Statistical mechanics of complex networks. Rev Mod Phys. 30 janv 2002;74(1):47–97.

[31] Cimini G, Squartini T, Saracco F, Garlaschelli D, Gabrielli A, Caldarelli G. The statistical physics of real-world networks. Nat Rev Phys. janv 2019;1(1):58–71.

[32] Gao YG, Selmer M, Dunham CM, Weixlbaumer A, Kelley AC, Ramakrishnan V. The Structure of the Ribosome with Elongation Factor G Trapped in the Posttranslocational State. Science. 30 oct 2009;326(5953):694–9. doi: 10.1126/science.1179709 19833919 PMC3763468

[33] Tourigny DS, Fernández IS, Kelley AC, Ramakrishnan V. Elongation Factor G Bound to the Ribosome in an Intermediate State of Translocation. Science (Internet). juin 2013 (cité 20 juin 2022); Disponible sur: https://www.science.org/doi/10.1126/science.1235490 10.1126/science.1235490 PMC3836249 23812720

[34] Zhou J, Lancaster L, Donohue JP, Noller HF. Crystal Structures of EF-G–Ribosome Complexes Trapped in Intermediate States of Translocation. Science (Internet). juin 2013 (cité 20 juin 2022); Disponible sur: https://www.science.org/doi/10.1126/science.1236086 23812722 10.1126/science.1236086 PMC3979973

[35] Polikanov YS, Steitz TA, Innis CA. A proton wire to couple aminoacyl-tRNA accommodation and peptide-bond formation on the ribosome. Nat Struct Mol Biol. sept 2014;21(9):787–93. doi: 10.1038/nsmb.2871 25132179 PMC4156881

[36] Bhaskar V, Graff-Meyer A, Schenk AD, Cavadini S, von Loeffelholz O, Natchiar SK, et al. Dynamics of uS19 C-Terminal Tail during the Translation Elongation Cycle in Human Ribosomes. Cell Rep. 7 avr 2020;31(1):107473. doi: 10.1016/j.celrep.2020.03.037 32268098

[37] Hagberg AA, Schult DA, Swart PJ. Exploring Network Structure, Dynamics, and Function using NetworkX. In: Proceedings of the Python in Science Conference (SciPy) (Internet). 2008 (cité 14 sept 2018). Disponible sur: http://conference.scipy.org/proceedings/SciPy2008/paper_2/

[38] Fortunato S. Community detection in graphs. Phys Rep. 1 févr 2010;486(3):75–174.

[39] Clauset A, Newman MEJ, Moore C. Finding community structure in very large networks. Phys Rev E. 6 déc 2004;70(6):066111. doi: 10.1103/PhysRevE.70.066111 15697438

[40] Newman M. Networks. 2 edition. New York, NY: Oxford University Press; 2018. 800 p.

[41] Mariani MS, Ren ZM, Bascompte J, Tessone CJ. Nestedness in complex networks: Observation, emergence, and implications. Phys Rep. 15 juin 2019;813:1–90.

[42] Almeida‐Neto M, Guimarães P, Guimarães PR, Loyola RD, Ulrich W. A consistent metric for nestedness analysis in ecological systems: reconciling concept and measurement. Oikos. 2008;117(8):1227–39.

[43] Freeman LC. Centrality in social networks conceptual clarification. Soc Netw. 1 janv 1978;1(3):215–39.

[44] Melnikov S, Ben-Shem A, Garreau de Loubresse N, Jenner L, Yusupova G, Yusupov M. One core, two shells: bacterial and eukaryotic ribosomes. Nat Struct Mol Biol. 5 juin 2012;19(6):560–7. doi: 10.1038/nsmb.2313 22664983

[45] Camprubí E, de Leeuw JW, House CH, Raulin F, Russell MJ, Spang A, et al. The Emergence of Life. Space Sci Rev. 12 déc 2019;215(8):56.

[46] Gouy R, Baurain D, Philippe H. Rooting the tree of life: the phylogenetic jury is still out. Philos Trans R Soc B Biol Sci. 26 sept 2015;370(1678):20140329. doi: 10.1098/rstb.2014.0329 26323760 PMC4571568

[47] de Farias ST, Jose MV, Prosdocimi F. Is it possible that cells have had more than one origin? Biosystems. 1 avr 2021;202:104371. 33524470 10.1016/j.biosystems.2021.104371

[48] Fox GE. Origin and Evolution of the Ribosome. Cold Spring Harb Perspect Biol (Internet). sept 2010 (cité 20 août 2018);2(9). Disponible sur: https://www.ncbi.nlm.nih.gov/pmc/articles/PMC2926754/ doi: 10.1101/cshperspect.a003483 20534711 PMC2926754

[49] Barabási AL, Albert R. Emergence of Scaling in Random Networks. Science. 15 oct 1999;286(5439):509–12. doi: 10.1126/science.286.5439.509 10521342

[50] Tirumalai MR, Rivas M, Tran Q, Fox GE. The Peptidyl Transferase Center: a Window to the Past. Microbiol Mol Biol Rev (Internet). 10 nov 2021 (cité 23 déc 2021); Disponible sur: https://journals.asm.org/doi/abs/10.1128/MMBR.00104-21 10.1128/MMBR.00104-21 PMC8579967 34756086

[51] Hury J, Nagaswamy U, Larios-Sanz M, Fox GE. Ribosome origins: The relative age of 23S rRNA Domains. Orig Life Evol Biospheres. 14 sept 2006;36(4):421. doi: 10.1007/s11084-006-9011-z 16972151

[52] Harish A, Caetano-Anollés G. Ribosomal History Reveals Origins of Modern Protein Synthesis. PLOS ONE. 12 mars 2012;7(3):e32776. doi: 10.1371/journal.pone.0032776 22427882 PMC3299690

[53] Root-Bernstein M, Root-Bernstein R. The ribosome as a missing link in the evolution of life. J Theor Biol. 21 févr 2015;367:130–58. doi: 10.1016/j.jtbi.2014.11.025 25500179

[54] Bokov K, Steinberg SV. A hierarchical model for evolution of 23S ribosomal RNA. Nature. févr 2009;457(7232):977–80. doi: 10.1038/nature07749 19225518

[55] Hsiao C, Mohan S, Kalahar BK, Williams LD. Peeling the Onion: Ribosomes Are Ancient Molecular Fossils. Mol Biol Evol. 1 nov 2009;26(11):2415–25. doi: 10.1093/molbev/msp163 19628620

[56] Sun F, Caetano-Anollés G. Menzerath–Altmann’s Law of Syntax in RNA Accretion History. Life. 27 mai 2021;11(6):489. doi: 10.3390/life11060489 34071925 PMC8228408

[57] Kornev AP, Taylor SS. Dynamics-Driven Allostery in Protein Kinases. Trends Biochem Sci. 1 nov 2015;40(11):628–47. doi: 10.1016/j.tibs.2015.09.002 26481499 PMC4630092

[58] Albert R, Jeong H, Barabási AL. Error and attack tolerance of complex networks. Nature. juill 2000;406(6794):378–82. doi: 10.1038/35019019 10935628

[59] Bowman JC, Petrov AS, Frenkel-Pinter M, Penev PI, Williams LD. Root of the Tree: The Significance, Evolution, and Origins of the Ribosome. Chem Rev. 10 juin 2020;120(11):4848–78. doi: 10.1021/acs.chemrev.9b00742 32374986

[60] Ben-Shem A, Garreau de Loubresse N, Melnikov S, Jenner L, Yusupova G, Yusupov M. The structure of the eukaryotic ribosome at 3.0 Å resolution. Science. 16 déc 2011;334(6062):1524–9. 22096102 10.1126/science.1212642

[61] Mears JA, Cannone JJ, Stagg SM, Gutell RR, Agrawal RK, Harvey SC. Modeling a minimal ribosome based on comparative sequence analysis. J Mol Biol. 9 août 2002;321(2):215–34. doi: 10.1016/s0022-2836(02)00568-5 12144780

[62] Kovacs NA, Petrov AS, Lanier KA, Williams LD. Frozen in Time: The History of Proteins. Mol Biol Evol. 1 mai 2017;34(5):1252–60. doi: 10.1093/molbev/msx086 28201543 PMC5400399

[63] Peña C, Hurt E, Panse VG. Eukaryotic ribosome assembly, transport and quality control. Nat Struct Mol Biol. sept 2017;24(9):689–99. doi: 10.1038/nsmb.3454 28880863

