# Supplementary material for: Modeling the ribosome as a bipartite graph
Source: PLoS One. 2022 Dec 30;17(12):e0279455. doi: 10.1371/journal.pone.0279455 (PMC9803165; doi:10.1371/journal.pone.0279455)
Supplement: S1 Table — 5OT7 corresponds to T. thermophilus and 6t7i corresponds to S. cerevisiae. (PDF) [file pone.0279455.s004.pdf]

Table SI1

5ot7	6t7i
5SrRNA	5.8rRNA
16S-3'M	5SrRNA
16S-3'm	18S-3'M
16S-5'	18S-3'm
16S-CD	18S-5'
23S-D0	18S-CD
23S-D1	25S-D0
23S-D2	25S-D1
23S-D3	25S-D2
23S-D4	25S-D3
23S-D5	25S-D4
23S-D6	25S-D5
EF-G	25S-D6
L1	L2-A
L2	L3
L3	L4-A
L4	L5
L5	L6-B
L6	L7-A
L9	L8-A
L13	L9-A
L14	L10
L15	L11-A
L16	L13-A
L17	L14-A
L18	L15-A
L19	L16-A
L20	L17-A
L21	L18-A
L22	L19-A
L23	L20-A
L24	L21-A
L25	L22-A
L27	L23-A
L28	L24-B
L29	L25
L30	L26-A
L31	L27-A
L32	L28
L33	L29
L34	L30
L35	L31-A
L36	L32
S2	L33-A
S3	L34-A
S4	L35-A
S5	L36-A

5ot7	6t7i
S6	L37-A
S7	L38
S8	L39
S9	L40
S10	L41-B
S11	L42-A
S12	L43-A
S13	Rps5p
S14	S0-A
S15	S1-A
S16	S2
S17	S3
S18	S4-A
S19	S6-A
S20	S7-A
Thx	S8-A
bL12	S9-A
mRNA	S10-A
tRNA-P	S11-A
uL10	S12
uL11	S13
	S14-A
	S15
	S16-A
	S17-B
	S18-A
	S19-A
	S20
	S21-A
	S22-A
	S23-A
	S24-A
	S25-A
	S26-A
	S27-A
	S28-B
	S29-A
	S30-A
	S31
	beta-like
	mRNA
	tRNA-E
	tRNA-P
