# Supplementary material for: Modeling the ribosome as a bipartite graph
Source: PLoS One. 2022 Dec 30;17(12):e0279455. doi: 10.1371/journal.pone.0279455 (PMC9803165; doi:10.1371/journal.pone.0279455)
Supplement: S2 Table — (PDF) [file pone.0279455.s005.pdf]

Table 2 Biadjacency matrix for pdb file 5ot7 (*T. thermophilus*)

	L13_23S-D2	L3_23S-D0	L16_23S-D2	L13_23S-D1	L13_23S-D6	L3_23S-D5	L36_23S-D2	L27_23S-D5	L14_23S-D0	L3_23S-D2	L3_L13	23S-D5_5SrRNA	uL11_23S-D2	L16_23S-D5	L32_23S-D2
**23S-D2**	1	1	1	1	1	1	1	1	0	1	1	1	1	1	1
**23S-D5**	1	1	1	0	1	1	1	1	1	1	1	1	0	1	1
**23S-D6**	1	1	0	1	1	1	1	0	1	0	1	0	0	0	0
**EF-G**	0	0	0	0	0	0	0	0	0	0	0	0	1	0	0
**L3**	1	1	0	0	1	1	0	0	1	1	1	0	0	0	1
**5SrRNA**	0	0	1	0	0	0	0	1	0	0	0	1	0	0	0
**L13**	1	1	0	1	1	1	1	0	0	1	1	0	0	0	0
**L16**	0	0	1	0	0	0	1	1	0	0	0	0	1	1	0
**L27**	0	0	1	0	0	0	0	1	0	0	0	1	0	1	0
**L36**	1	0	1	0	0	0	1	0	0	0	0	0	0	1	0
**L6**	0	0	0	0	0	0	1	0	0	0	0	0	0	0	0
**uL11**	0	0	1	0	0	0	0	0	0	0	0	0	1	0	0
**L25**	0	0	1	0	0	0	0	0	0	0	0	0	1	1	0
**L5**	0	0	0	0	0	0	0	0	0	0	0	1	0	0	0
**L18**	0	0	0	0	0	0	0	1	0	0	0	1	0	0	0
**L30**	0	0	0	0	0	0	0	0	0	0	0	0	0	0	0
**uL10**	0	0	0	0	0	0	0	0	0	0	0	0	1	0	0
**bL12**	0	0	0	0	0	0	0	0	0	0	0	0	0	0	0
**16S-CD**	0	0	0	0	0	0	0	0	0	0	0	0	0	0	0
**16S-3'M**	0	0	0	0	0	0	0	0	0	0	0	0	0	0	0
**16S-3'm**	0	0	0	0	0	0	0	0	0	0	0	0	0	0	0
**S13**	0	0	0	0	0	0	0	0	0	0	0	0	0	0	0
**mRNA**	0	0	0	0	0	0	0	0	0	0	0	0	0	0	0
**S9**	0	0	0	0	0	0	0	0	0	0	0	0	0	0	0
**S19**	0	0	0	0	0	0	0	0	0	0	0	0	0	0	0
**S7**	0	0	0	0	0	0	0	0	0	0	0	0	0	0	0
**S3**	0	0	0	0	0	0	0	0	0	0	0	0	0	0	0
**L31**	0	0	0	0	0	0	0	0	0	0	0	0	0	0	0
**S11**	0	0	0	0	0	0	0	0	0	0	0	0	0	0	0
**S14**	0	0	0	0	0	0	0	0	0	0	0	0	0	0	0
**S10**	0	0	0	0	0	0	0	0	0	0	0	0	0	0	0
**S6**	0	0	0	0	0	0	0	0	0	0	0	0	0	0	0
**Thx**	0	0	0	0	0	0	0	0	0	0	0	0	0	0	0
**S18**	0	0	0	0	0	0	0	0	0	0	0	0	0	0	0
**23S-D1**	1	0	0	1	1	0	0	0	0	0	0	0	0	0	0
**tRNA-P**	0	0	0	0	0	0	0	0	0	0	0	0	0	0	0
**23S-D4**	0	0	0	0	0	0	0	0	1	0	0	0	0	0	0
**23S-D3**	0	0	0	0	0	0	0	0	0	0	0	0	0	0	0
**L15**	0	0	0	0	0	0	0	0	0	0	0	0	0	0	0
**L28**	0	0	0	0	0	0	0	0	0	0	0	0	0	0	0
**L35**	0	0	0	0	0	0	0	1	0	0	0	0	0	0	0
**L4**	0	0	0	0	0	0	0	0	0	0	0	0	0	0	0

	L13_23S-D2	L3_23S-D0	L16_23S-D2	L13_23S-D1	L13_23S-D6	L3_23S-D5	L36_23S-D2	L27_23S-D5	L14_23S-D0	L3_23S-D2	L3_L13	23S-D5_5SrRNA	uL11_23S-D2	L16_23S-D5	L32_23S-D2
**L34**	0	0	0	0	0	0	0	0	0	0	0	0	0	0	0
**L2**	0	0	0	0	0	0	0	0	0	0	0	0	0	0	0
**L9**	0	0	0	0	0	0	0	0	0	0	0	0	0	0	0
**L23**	0	0	0	0	0	0	0	0	0	0	0	0	0	0	0
**L33**	0	0	0	0	0	0	0	0	0	0	0	0	0	0	0
**L29**	0	0	0	0	0	0	0	0	0	0	0	0	0	0	0
**L1**	0	0	0	0	0	0	0	0	0	0	0	0	0	0	0
**L24**	0	0	0	0	0	0	0	0	0	0	0	0	0	0	0
**23S-D0**	1	1	0	1	1	1	0	0	1	1	1	0	0	0	1
**L32**	0	1	0	0	0	1	0	0	0	1	0	0	0	0	1
**L20**	1	0	0	1	0	0	0	0	0	0	0	0	0	0	0
**L22**	0	1	0	0	0	0	0	0	0	1	0	0	0	0	1
**L14**	0	1	0	0	0	0	0	0	1	0	0	0	0	0	0
**L17**	0	1	0	0	0	0	0	0	0	0	0	0	0	0	0
**L19**	0	0	0	0	0	0	0	0	1	0	0	0	0	0	0
**L21**	1	0	0	1	0	0	0	0	0	0	0	0	0	0	0
**16S-5'**	0	0	0	0	0	0	0	0	0	0	0	0	0	0	0
**S8**	0	0	0	0	0	0	0	0	0	0	0	0	0	0	0
**S12**	0	0	0	0	0	0	0	0	0	0	0	0	0	0	0
**S5**	0	0	0	0	0	0	0	0	0	0	0	0	0	0	0
**S17**	0	0	0	0	0	0	0	0	0	0	0	0	0	0	0
**S4**	0	0	0	0	0	0	0	0	0	0	0	0	0	0	0
**S15**	0	0	0	0	0	0	0	0	0	0	0	0	0	0	0
**S2**	0	0	0	0	0	0	0	0	0	0	0	0	0	0	0
**S20**	0	0	0	0	0	0	0	0	0	0	0	0	0	0	0
**S16**	0	0	0	0	0	0	0	0	0	0	0	0	0	0	0

	23S-D2_5SrRNA	L13_23S-D0	L18_5SrRNA	L14_23S-D6	L6_23S-D6	L36_23S-D5	EF-G_23S-D2	L32_23S-D5	L5_5SrRNA	L13_23S-D5	L25_L16	EF-G_L6	L30_23S-D2	L36_23S-D6	L6_23S-D5	L27_L16
**23S-D2**	1	1	0	0	1	1	1	1	0	1	1	1	1	1	0	1
**23S-D5**	1	0	1	1	1	1	1	1	1	1	1	1	0	1	1	1
**23S-D6**	0	1	0	1	1	1	0	0	0	1	0	1	0	1	1	0
**EF-G**	0	0	0	0	1	0	1	0	0	0	0	1	0	0	1	0
**L3**	0	1	0	1	0	0	0	1	0	1	0	0	0	0	0	0
**5SrRNA**	1	0	1	0	0	0	0	0	1	0	1	0	1	0	0	0
**L13**	0	1	0	0	0	0	0	0	0	1	0	0	0	0	0	0
**L16**	1	0	0	0	0	1	0	0	0	0	1	0	0	0	0	1
**L27**	0	0	1	0	0	0	0	0	0	0	0	0	1	0	0	1
**L36**	0	0	0	0	1	1	0	0	0	0	0	0	0	1	1	0
**L6**	0	0	0	0	1	1	1	0	0	0	0	1	0	1	1	0
**uL11**	0	0	0	0	0	0	1	0	0	0	0	0	0	0	0	0
**L25**	1	0	0	0	0	0	0	0	0	0	1	0	0	0	0	0
**L5**	0	0	1	0	0	0	0	0	1	0	0	0	0	0	0	0
**L18**	0	0	1	0	0	0	0	0	1	0	0	0	0	0	0	0
**L30**	1	0	0	0	0	0	0	0	0	0	0	0	1	0	0	0
**uL10**	0	0	0	0	0	0	0	0	0	0	0	0	0	0	0	0
**bL12**	0	0	0	0	0	0	0	0	0	0	0	0	0	0	0	0
**16S-CD**	0	0	0	0	0	0	0	0	0	0	0	0	0	0	0	0
**16S-3'M**	0	0	0	0	0	0	0	0	0	0	0	0	0	0	0	0
**16S-3'm**	0	0	0	0	0	0	0	0	0	0	0	0	0	0	0	0
**S13**	0	0	0	0	0	0	0	0	0	0	0	0	0	0	0	0
**mRNA**	0	0	0	0	0	0	0	0	0	0	0	0	0	0	0	0
**S9**	0	0	0	0	0	0	0	0	0	0	0	0	0	0	0	0
**S19**	0	0	0	0	0	0	0	0	0	0	0	0	0	0	0	0
**S7**	0	0	0	0	0	0	0	0	0	0	0	0	0	0	0	0
**S3**	0	0	0	0	0	0	0	0	0	0	0	0	0	0	0	0
**L31**	0	0	1	0	0	0	0	0	1	0	0	0	0	0	0	0
**S11**	0	0	0	0	0	0	0	0	0	0	0	0	0	0	0	0
**S14**	0	0	0	0	0	0	0	0	0	0	0	0	0	0	0	0
**S10**	0	0	0	0	0	0	0	0	0	0	0	0	0	0	0	0
**S6**	0	0	0	0	0	0	0	0	0	0	0	0	0	0	0	0
**Thx**	0	0	0	0	0	0	0	0	0	0	0	0	0	0	0	0
**S18**	0	0	0	0	0	0	0	0	0	0	0	0	0	0	0	0
**23S-D1**	0	1	0	0	0	0	0	0	0	0	0	0	0	0	0	0
**tRNA-P**	0	0	0	0	0	0	0	0	0	0	0	0	0	0	0	0
**23S-D4**	0	0	0	0	0	0	0	0	0	0	0	0	0	0	0	0
**23S-D3**	0	0	0	0	0	0	0	0	0	0	0	0	0	0	0	0
**L15**	0	0	0	0	0	0	0	0	0	0	0	0	0	0	0	0
**L28**	0	0	0	0	0	0	0	0	0	0	0	0	0	0	0	0
**L35**	0	0	0	0	0	0	0	0	0	0	0	0	0	0	0	0
**L4**	0	0	0	0	0	0	0	0	0	0	0	0	0	0	0	0

	23S-D2_5SrRNA	L13_23S-D0	L18_5SrRNA	L14_23S-D6	L6_23S-D6	L36_23S-D5	EF-G_23S-D2	L32_23S-D5	L5_5SrRNA	L13_23S-D5	L25_L16	EF-G_L6	L30_23S-D2	L36_23S-D6	L6_23S-D5	L27_L16
**L34**	0	0	0	0	0	0	0	0	0	0	0	0	0	0	0	0
**L2**	0	0	0	0	0	0	0	0	0	0	0	0	0	0	0	0
**L9**	0	0	0	0	0	0	0	0	0	0	0	0	0	0	0	0
**L23**	0	0	0	0	0	0	0	0	0	0	0	0	0	0	0	0
**L33**	0	0	0	0	0	0	0	0	0	0	0	0	0	0	0	0
**L29**	0	0	0	0	0	0	0	0	0	0	0	0	0	0	0	0
**L1**	0	0	0	0	0	0	0	0	0	0	0	0	0	0	0	0
**L24**	0	0	0	0	0	0	0	0	0	0	0	0	0	0	0	0
**23S-D0**	0	1	0	1	0	0	0	1	0	0	0	0	0	0	0	0
**L32**	0	0	0	0	0	0	0	1	0	0	0	0	0	0	0	0
**L20**	0	0	0	0	0	0	0	0	0	0	0	0	1	0	0	0
**L22**	0	0	0	0	0	0	0	0	0	0	0	0	0	0	0	0
**L14**	0	0	0	1	0	0	0	0	0	0	0	0	0	0	0	0
**L17**	0	0	0	0	0	0	0	0	0	0	0	0	0	0	0	0
**L19**	0	0	0	1	0	0	0	0	0	0	0	0	0	0	0	0
**L21**	0	0	0	0	0	0	0	0	0	0	0	0	0	0	0	0
**16S-5'**	0	0	0	0	0	0	0	0	0	0	0	0	0	0	0	0
**S8**	0	0	0	0	0	0	0	0	0	0	0	0	0	0	0	0
**S12**	0	0	0	0	0	0	0	0	0	0	0	0	0	0	0	0
**S5**	0	0	0	0	0	0	0	0	0	0	0	0	0	0	0	0
**S17**	0	0	0	0	0	0	0	0	0	0	0	0	0	0	0	0
**S4**	0	0	0	0	0	0	0	0	0	0	0	0	0	0	0	0
**S15**	0	0	0	0	0	0	0	0	0	0	0	0	0	0	0	0
**S2**	0	0	0	0	0	0	0	0	0	0	0	0	0	0	0	0
**S20**	0	0	0	0	0	0	0	0	0	0	0	0	0	0	0	0
**S16**	0	0	0	0	0	0	0	0	0	0	0	0	0	0	0	0

	L36_L16	L4_23S-D5	L18_23S-D5	L27_23S-D2	L31_5SrRNA	L27_L18	L25_23S-D2	L25_5SrRNA	EF-G_23S-D5	L6_23S-D2	L16_5SrRNA	L5_23S-D5	L33_23S-D2	uL10_uL11	EF-G_uL11	EF-G_23S-D6
**23S-D2**	1	1	0	1	0	0	1	1	1	1	1	0	1	1	1	0
**23S-D5**	1	1	1	1	0	1	0	0	1	0	0	1	1	0	0	0
**23S-D6**	0	0	0	0	0	0	0	0	0	1	0	0	0	0	0	1
**EF-G**	0	0	0	0	0	0	0	0	1	0	0	0	0	0	1	1
**L3**	0	0	0	0	0	0	0	0	0	0	0	0	0	0	0	0
**5SrRNA**	0	0	1	0	1	1	0	1	0	0	1	1	0	0	0	0
**L13**	0	0	0	0	0	0	0	0	0	0	0	0	0	0	0	0
**L16**	1	0	0	0	0	0	1	1	0	0	1	0	0	0	0	0
**L27**	0	0	1	1	0	1	0	0	0	0	0	0	0	0	0	0
**L36**	1	0	0	0	0	0	0	0	0	1	0	0	0	0	0	0
**L6**	0	0	0	0	0	0	0	0	1	1	0	0	0	0	0	1
**uL11**	0	0	0	0	0	0	1	0	0	0	0	0	0	1	1	0
**L25**	0	0	0	0	0	0	1	1	0	0	1	0	0	0	0	0
**L5**	0	0	0	0	1	0	0	0	0	0	0	1	0	0	0	0
**L18**	0	0	1	0	1	1	0	0	0	0	0	0	0	0	0	0
**L30**	0	0	0	1	0	0	0	0	0	0	0	0	0	0	0	0
**uL10**	0	0	0	0	0	0	0	0	0	0	0	0	0	1	0	0
**bL12**	0	0	0	0	0	0	0	0	0	0	0	0	0	0	0	0
**16S-CD**	0	0	0	0	0	0	0	0	0	0	0	0	0	0	0	0
**16S-3'M**	0	0	0	0	0	0	0	0	0	0	0	0	0	0	0	0
**16S-3'm**	0	0	0	0	0	0	0	0	0	0	0	0	0	0	0	0
**S13**	0	0	0	0	0	0	0	0	0	0	0	0	0	0	0	0
**mRNA**	0	0	0	0	0	0	0	0	0	0	0	0	0	0	0	0
**S9**	0	0	0	0	0	0	0	0	0	0	0	0	0	0	0	0
**S19**	0	0	0	0	0	0	0	0	0	0	0	0	0	0	0	0
**S7**	0	0	0	0	0	0	0	0	0	0	0	0	0	0	0	0
**S3**	0	0	0	0	0	0	0	0	0	0	0	0	0	0	0	0
**L31**	0	0	0	0	1	0	0	0	0	0	0	0	0	0	0	0
**S11**	0	0	0	0	0	0	0	0	0	0	0	0	0	0	0	0
**S14**	0	0	0	0	0	0	0	0	0	0	0	0	0	0	0	0
**S10**	0	0	0	0	0	0	0	0	0	0	0	0	0	0	0	0
**S6**	0	0	0	0	0	0	0	0	0	0	0	0	0	0	0	0
**Thx**	0	0	0	0	0	0	0	0	0	0	0	0	0	0	0	0
**S18**	0	0	0	0	0	0	0	0	0	0	0	0	0	0	0	0
**23S-D1**	0	0	0	0	0	0	0	0	0	0	0	0	0	0	0	0
**tRNA-P**	0	0	0	0	0	0	0	0	0	0	0	0	0	0	0	0
**23S-D4**	0	0	0	0	0	0	0	0	0	0	0	0	0	0	0	0
**23S-D3**	0	0	0	0	0	0	0	0	0	0	0	0	0	0	0	0
**L15**	0	0	0	0	0	0	0	0	0	0	0	0	0	0	0	0
**L28**	0	0	0	0	0	0	0	0	0	0	0	0	0	0	0	0
**L35**	0	0	0	0	0	0	0	0	0	0	0	0	0	0	0	0
**L4**	0	1	0	0	0	0	0	0	0	0	0	0	0	0	0	0

	L36_L16	L4_23S-D5	L18_23S-D5	L27_23S-D2	L31_5SrRNA	L27_L18	L25_23S-D2	L25_5SrRNA	EF-G_23S-D5	L6_23S-D2	L16_5SrRNA	L5_23S-D5	L33_23S-D2	uL10_uL11	EF-G_uL11	EF-G_23S-D6
**L34**	0	0	0	0	0	0	0	0	0	0	0	0	0	0	0	0
**L2**	0	0	0	0	0	0	0	0	0	0	0	0	0	0	0	0
**L9**	0	0	0	0	0	0	0	0	0	0	0	0	0	0	0	0
**L23**	0	0	0	0	0	0	0	0	0	0	0	0	0	0	0	0
**L33**	0	0	0	0	0	0	0	0	0	0	0	0	1	0	0	0
**L29**	0	0	0	0	0	0	0	0	0	0	0	0	0	0	0	0
**L1**	0	0	0	0	0	0	0	0	0	0	0	0	0	0	0	0
**L24**	0	0	0	0	0	0	0	0	0	0	0	0	0	0	0	0
**23S-D0**	0	1	0	0	0	0	0	0	0	0	0	0	0	0	0	0
**L32**	0	0	0	0	0	0	0	0	0	0	0	0	0	0	0	0
**L20**	0	0	0	0	0	0	0	0	0	0	0	0	0	0	0	0
**L22**	0	0	0	0	0	0	0	0	0	0	0	0	0	0	0	0
**L14**	0	0	0	0	0	0	0	0	0	0	0	0	0	0	0	0
**L17**	0	0	0	0	0	0	0	0	0	0	0	0	0	0	0	0
**L19**	0	0	0	0	0	0	0	0	0	0	0	0	0	0	0	0
**L21**	0	0	0	0	0	0	0	0	0	0	0	0	0	0	0	0
**16S-5'**	0	0	0	0	0	0	0	0	0	0	0	0	0	0	0	0
**S8**	0	0	0	0	0	0	0	0	0	0	0	0	0	0	0	0
**S12**	0	0	0	0	0	0	0	0	0	0	0	0	0	0	0	0
**S5**	0	0	0	0	0	0	0	0	0	0	0	0	0	0	0	0
**S17**	0	0	0	0	0	0	0	0	0	0	0	0	0	0	0	0
**S4**	0	0	0	0	0	0	0	0	0	0	0	0	0	0	0	0
**S15**	0	0	0	0	0	0	0	0	0	0	0	0	0	0	0	0
**S2**	0	0	0	0	0	0	0	0	0	0	0	0	0	0	0	0
**S20**	0	0	0	0	0	0	0	0	0	0	0	0	0	0	0	0
**S16**	0	0	0	0	0	0	0	0	0	0	0	0	0	0	0	0

	uL10_23S-D2	L30_5SrRNA	L27_5SrRNA	uL11_bL12	EF-G_bL12	L36_L6	S13_16S-3'M	S9_16S-3'M	S3_16S-3'M	16S-3'm_mRNA	16S-3'M_tRNA-P	S19_16S-3'M	S11_16S-CD	tRNA-P_mRNA
**23S-D2**	1	1	0	0	0	0	0	0	0	0	0	0	0	0
**23S-D5**	0	0	1	0	0	0	0	0	0	0	0	0	1	0
**23S-D6**	0	0	0	0	0	0	0	0	0	0	0	0	0	0
**EF-G**	0	0	0	0	1	0	1	0	0	1	0	1	0	1
**L3**	0	0	0	0	0	0	0	0	0	0	0	0	0	0
**5SrRNA**	0	1	1	0	0	0	0	0	0	0	0	0	0	0
**L13**	0	0	0	0	0	0	0	0	0	0	0	0	0	0
**L16**	0	0	0	0	0	0	0	0	0	0	0	0	0	0
**L27**	0	0	1	0	0	0	0	0	0	0	0	0	0	0
**L36**	0	0	0	0	0	1	0	0	0	0	0	0	0	0
**L6**	0	0	0	0	0	1	0	0	0	0	0	0	0	0
**uL11**	1	0	0	1	0	0	0	0	0	0	0	0	0	0
**L25**	0	0	0	0	0	0	0	0	0	0	0	0	0	0
**L5**	0	0	0	0	0	0	0	0	0	0	0	0	0	0
**L18**	0	0	0	0	0	0	0	0	0	0	0	0	0	0
**L30**	0	1	0	0	0	0	0	0	0	0	0	0	0	0
**uL10**	1	0	0	0	0	0	0	0	0	0	0	0	0	0
**bL12**	0	0	0	1	1	0	0	0	0	0	0	0	0	0
**16S-CD**	0	0	0	0	0	0	0	0	0	1	1	0	1	1
**16S-3'M**	0	0	0	0	0	0	1	1	1	1	1	1	0	1
**16S-3'm**	0	0	0	0	0	0	0	0	0	1	1	0	1	1
**S13**	0	0	0	0	0	0	1	1	0	0	1	1	0	0
**mRNA**	0	0	0	0	0	0	0	0	0	1	1	0	1	1
**S9**	0	0	0	0	0	0	1	1	1	0	1	0	0	1
**S19**	0	0	0	0	0	0	1	0	0	0	1	1	0	0
**S7**	0	0	0	0	0	0	1	1	0	1	0	0	1	1
**S3**	0	0	0	0	0	0	0	1	1	0	0	0	0	0
**L31**	0	0	0	0	0	0	1	0	0	0	0	1	0	0
**S11**	0	0	0	0	0	0	0	0	0	1	0	0	1	0
**S14**	0	0	0	0	0	0	1	1	1	0	0	1	0	0
**S10**	0	0	0	0	0	0	0	1	1	0	0	0	0	0
**S6**	0	0	0	0	0	0	0	0	0	0	0	0	1	0
**Thx**	0	0	0	0	0	0	1	1	0	0	0	1	0	0
**S18**	0	0	0	0	0	0	0	0	0	0	0	0	1	0
**23S-D1**	0	0	0	0	0	0	0	0	0	0	0	0	0	0
**tRNA-P**	0	0	0	0	0	0	1	1	0	1	1	1	0	1
**23S-D4**	0	0	0	0	0	0	0	0	0	0	0	0	0	0
**23S-D3**	0	0	0	0	0	0	0	0	0	0	0	0	0	0
**L15**	0	0	0	0	0	0	0	0	0	0	0	0	0	0
**L28**	0	0	0	0	0	0	0	0	0	0	0	0	0	0
**L35**	0	0	0	0	0	0	0	0	0	0	0	0	0	0
**L4**	0	0	0	0	0	0	0	0	0	0	0	0	0	0

	uL10_23S-D2	L30_5SrRNA	L27_5SrRNA	uL11_bL12	EF-G_bL12	L36_L6	S13_16S-3'M	S9_16S-3'M	S3_16S-3'M	16S-3'm_mRNA	16S-3'M_tRNA-P	S19_16S-3'M	S11_16S-CD	tRNA-P_mRNA
**L34**	0	0	0	0	0	0	0	0	0	0	0	0	0	0
**L2**	0	0	0	0	0	0	0	0	0	0	0	0	0	0
**L9**	0	0	0	0	0	0	0	0	0	0	0	0	0	0
**L23**	0	0	0	0	0	0	0	0	0	0	0	0	0	0
**L33**	0	0	0	0	0	0	0	0	0	0	0	0	0	0
**L29**	0	0	0	0	0	0	0	0	0	0	0	0	0	0
**L1**	0	0	0	0	0	0	0	0	0	0	0	0	0	0
**L24**	0	0	0	0	0	0	0	0	0	0	0	0	0	0
**23S-D0**	0	0	0	0	0	0	0	0	0	0	0	0	0	0
**L32**	0	0	0	0	0	0	0	0	0	0	0	0	0	0
**L20**	0	0	0	0	0	0	0	0	0	0	0	0	0	0
**L22**	0	0	0	0	0	0	0	0	0	0	0	0	0	0
**L14**	0	0	0	0	0	0	0	0	0	0	0	0	0	0
**L17**	0	0	0	0	0	0	0	0	0	0	0	0	0	0
**L19**	0	0	0	0	0	0	0	0	0	0	0	0	0	0
**L21**	0	0	0	0	0	0	0	0	0	0	0	0	0	0
**16S-5'**	0	0	0	0	0	0	0	0	1	0	0	0	0	0
**S8**	0	0	0	0	0	0	0	0	0	0	0	0	0	0
**S12**	0	0	0	0	0	0	0	0	0	0	0	0	0	0
**S5**	0	0	0	0	0	0	0	0	1	0	0	0	0	0
**S17**	0	0	0	0	0	0	0	0	0	0	0	0	0	0
**S4**	0	0	0	0	0	0	0	0	0	0	0	0	0	0
**S15**	0	0	0	0	0	0	0	0	0	0	0	0	0	0
**S2**	0	0	0	0	0	0	0	0	1	0	0	0	0	0
**S20**	0	0	0	0	0	0	0	0	0	0	0	0	0	0
**S16**	0	0	0	0	0	0	0	0	0	0	0	0	0	0

	16S-CD_mRNA	S14_16S-3'M	S7_mRNA	Thx_16S-3'M	S13_S19	S10_16S-3'M	S6_16S-CD	16S-3'M_mRNA	S7_16S-3'M	16S-3'm_23S-D4	16S-CD_tRNA-P	EF-G_16S-3'M	S10_S14	S9_tRNA-P
**23S-D2**	0	0	0	0	1	0	0	0	0	0	0	0	0	0
**23S-D5**	0	0	0	0	0	0	0	0	0	0	0	0	0	0
**23S-D6**	0	0	0	0	0	0	0	0	0	0	0	0	0	0
**EF-G**	0	0	0	0	0	1	0	0	0	1	0	1	0	0
**L3**	0	0	0	0	0	0	0	0	0	0	0	0	0	0
**5SrRNA**	0	0	0	0	0	0	0	0	0	0	0	0	0	0
**L13**	0	0	0	0	0	0	0	0	0	0	0	0	0	0
**L16**	0	0	0	0	0	0	0	0	0	0	0	0	0	0
**L27**	0	0	0	0	0	0	0	0	0	0	0	0	0	0
**L36**	0	0	0	0	0	0	0	0	0	0	0	0	0	0
**L6**	0	0	0	0	0	0	0	0	0	0	0	0	0	0
**uL11**	0	0	0	0	0	0	0	0	0	0	0	0	0	0
**L25**	0	0	0	0	0	0	0	0	0	0	0	0	0	0
**L5**	0	0	0	0	0	0	0	0	0	0	0	0	0	0
**L18**	0	0	0	0	0	0	0	0	0	0	0	0	0	0
**L30**	0	0	0	0	0	0	0	0	0	0	0	0	0	0
**uL10**	0	0	0	0	0	0	0	0	0	0	0	0	0	0
**bL12**	0	0	0	0	0	0	0	0	0	0	0	0	0	0
**16S-CD**	1	0	1	0	0	0	1	1	0	1	1	0	0	0
**16S-3'M**	1	1	1	1	1	1	0	1	1	0	1	1	1	1
**16S-3'm**	1	0	1	0	0	0	0	1	0	1	0	0	0	1
**S13**	0	1	0	1	1	0	0	0	1	0	0	1	0	0
**mRNA**	1	0	1	0	0	0	0	1	1	0	1	0	0	1
**S9**	0	1	0	1	0	1	0	0	1	0	0	0	1	1
**S19**	0	1	0	1	1	0	0	0	0	0	0	1	0	0
**S7**	1	0	1	1	0	0	0	1	1	0	0	0	0	0
**S3**	0	1	0	0	0	1	0	0	0	0	0	0	1	0
**L31**	0	0	0	0	1	0	0	0	0	0	0	0	0	0
**S11**	1	0	1	0	0	0	1	0	0	0	0	0	0	0
**S14**	0	1	0	0	0	1	0	0	0	0	0	0	1	0
**S10**	0	1	0	0	0	1	0	0	0	0	0	1	1	0
**S6**	0	0	0	0	0	0	1	0	0	0	0	0	0	0
**Thx**	0	0	0	1	0	0	0	0	1	0	0	0	0	0
**S18**	0	0	0	0	0	0	1	0	0	0	0	0	0	0
**23S-D1**	0	0	0	0	0	0	0	0	0	0	0	0	0	0
**tRNA-P**	1	0	1	0	1	0	0	1	0	0	1	0	0	1
**23S-D4**	0	0	0	0	0	0	0	0	0	1	1	0	0	0
**23S-D3**	0	0	0	0	0	0	0	0	0	0	0	0	0	0
**L15**	0	0	0	0	0	0	0	0	0	0	0	0	0	0
**L28**	0	0	0	0	0	0	0	0	0	0	0	0	0	0
**L35**	0	0	0	0	0	0	0	0	0	0	0	0	0	0
**L4**	0	0	0	0	0	0	0	0	0	0	0	0	0	0

	16S-CD_mRNA	S14_16S-3'M	S7_mRNA	Thx_16S-3'M	S13_S19	S10_16S-3'M	S6_16S-CD	16S-3'M_mRNA	S7_16S-3'M	16S-3'm_23S-D4	16S-CD_tRNA-P	EF-G_16S-3'M	S10_S14	S9_tRNA-P
**L34**	0	0	0	0	0	0	0	0	0	0	0	0	0	0
**L2**	0	0	0	0	0	0	1	0	0	0	0	0	0	0
**L9**	0	0	0	0	0	0	0	0	0	0	0	0	0	0
**L23**	0	0	0	0	0	0	0	0	0	0	0	0	0	0
**L33**	0	0	0	0	0	0	0	0	0	0	0	0	0	0
**L29**	0	0	0	0	0	0	0	0	0	0	0	0	0	0
**L1**	0	0	0	0	0	0	0	0	0	0	0	0	0	0
**L24**	0	0	0	0	0	0	0	0	0	0	0	0	0	0
**23S-D0**	0	0	0	0	0	0	0	0	0	0	0	0	0	0
**L32**	0	0	0	0	0	0	0	0	0	0	0	0	0	0
**L20**	0	0	0	0	0	0	0	0	0	0	0	0	0	0
**L22**	0	0	0	0	0	0	0	0	0	0	0	0	0	0
**L14**	0	0	0	0	0	0	0	0	0	1	0	0	0	0
**L17**	0	0	0	0	0	0	0	0	0	0	0	0	0	0
**L19**	0	0	0	0	0	0	0	0	0	0	0	0	0	0
**L21**	0	0	0	0	0	0	0	0	0	0	0	0	0	0
**16S-5'**	0	0	0	0	0	0	0	0	0	0	0	0	0	0
**S8**	0	0	0	0	0	0	0	0	0	0	0	0	0	0
**S12**	0	0	0	0	0	0	0	0	0	1	0	0	0	0
**S5**	0	0	0	0	0	0	0	0	0	0	0	0	0	0
**S17**	0	0	0	0	0	0	0	0	0	0	0	0	0	0
**S4**	0	0	0	0	0	0	0	0	0	0	0	0	0	0
**S15**	0	0	0	0	0	0	1	0	0	0	0	0	0	0
**S2**	0	0	0	0	0	0	0	0	0	0	0	0	0	0
**S20**	0	0	0	0	0	0	0	0	0	0	0	0	0	0
**S16**	0	0	0	0	0	0	0	0	0	0	0	0	0	0

	16S-3'm_tRNA-P	S13_L31	S11_16S-3'm	S13_tRNA-P	S19_L31	L31_16S-3'M	S2_S8	S3_S14	S19_tRNA-P	S7_S11	S7_16S-CD	S9_S14	S9_S10	S3_S10	L31_L5	S18_16S-CD	EF-G_mRNA	EF-G_tRNA-P
**23S-D2**	0	0	0	0	0	0	0	0	0	0	0	0	0	0	0	0	0	0
**23S-D5**	0	0	0	0	0	0	0	0	0	0	0	0	0	0	0	0	0	0
**23S-D6**	0	0	0	0	0	0	0	0	0	0	0	0	0	0	0	0	0	0
**EF-G**	0	0	0	0	0	0	0	0	0	0	0	0	0	0	0	0	1	1
**L3**	0	0	0	0	0	0	0	0	0	0	0	0	0	0	0	0	0	0
**5SrRNA**	0	0	0	0	0	0	0	0	0	0	0	0	0	0	1	0	0	0
**L13**	0	0	0	0	0	0	0	0	0	0	0	0	0	0	0	0	0	0
**L16**	0	0	0	0	0	0	0	0	0	0	0	0	0	0	0	0	0	0
**L27**	0	0	0	0	0	0	0	0	0	0	0	0	0	0	0	0	0	0
**L36**	0	0	0	0	0	0	0	0	0	0	0	0	0	0	0	0	0	0
**L6**	0	0	0	0	0	0	0	0	0	0	0	0	0	0	0	0	0	0
**uL11**	0	0	0	0	0	0	0	0	0	0	0	0	0	0	0	0	0	0
**L25**	0	0	0	0	0	0	0	0	0	0	0	0	0	0	0	0	0	0
**L5**	0	1	0	0	0	0	0	0	0	0	0	0	0	0	1	0	0	0
**L18**	0	0	0	0	0	0	0	0	0	0	0	0	0	0	0	0	0	0
**L30**	0	0	0	0	0	0	0	0	0	0	0	0	0	0	0	0	0	0
**uL10**	0	0	0	0	0	0	0	0	0	0	0	0	0	0	0	0	0	0
**bL12**	0	0	0	0	0	0	0	0	0	0	0	0	0	0	0	0	0	0
**16S-CD**	0	0	1	0	0	0	1	0	0	1	1	0	0	0	0	1	0	0
**16S-3'M**	1	1	0	1	1	1	1	1	1	0	0	1	1	1	0	0	0	0
**16S-3'm**	1	0	1	0	0	0	0	0	0	0	0	0	0	0	0	0	1	1
**S13**	0	1	0	1	1	1	0	0	1	0	0	0	0	0	1	0	0	0
**mRNA**	1	0	1	0	0	0	0	0	0	1	1	0	0	0	0	0	1	1
**S9**	1	0	0	0	0	0	0	0	0	0	0	1	1	0	0	0	0	0
**S19**	0	1	0	1	1	1	0	0	1	0	0	0	0	0	0	0	0	0
**S7**	0	0	0	0	0	0	0	0	0	1	1	0	0	0	0	0	0	0
**S3**	0	0	0	0	0	0	0	1	0	0	0	0	0	1	0	0	0	0
**L31**	0	1	0	0	1	1	0	0	0	0	0	0	0	0	1	0	0	0
**S11**	0	0	1	0	0	0	0	0	0	1	1	0	0	0	0	1	0	0
**S14**	0	0	0	0	0	0	0	1	0	0	0	1	1	1	0	0	0	0
**S10**	0	0	0	0	0	0	0	1	0	0	0	1	1	1	0	0	0	0
**S6**	0	0	0	0	0	0	0	0	0	0	0	0	0	0	0	1	0	0
**Thx**	0	0	0	0	0	0	0	0	0	0	0	0	0	0	0	0	0	0
**S18**	0	0	0	0	0	0	0	0	0	0	0	0	0	0	0	1	0	0
**23S-D1**	0	0	0	0	0	0	0	0	0	0	0	0	0	0	0	0	0	0
**tRNA-P**	1	0	0	1	0	0	0	0	1	0	0	0	0	0	0	0	1	1
**23S-D4**	0	0	0	0	0	0	0	0	0	0	0	0	0	0	0	0	0	0
**23S-D3**	0	0	0	0	0	0	0	0	0	0	0	0	0	0	0	0	0	0
**L15**	0	0	0	0	0	0	0	0	0	0	0	0	0	0	0	0	0	0
**L28**	0	0	0	0	0	0	0	0	0	0	0	0	0	0	0	0	0	0
**L35**	0	0	0	0	0	0	0	0	0	0	0	0	0	0	0	0	0	0
**L4**	0	0	0	0	0	0	0	0	0	0	0	0	0	0	0	0	0	0

	16S-3'm_tRNA-P	S13_L31	S11_16S-3'm	S13_tRNA-P	S19_L31	L31_16S-3'M	S2_S8	S3_S14	S19_tRNA-P	S7_S11	S7_16S-CD	S9_S14	S9_S10	S3_S10	L31_L5	S18_16S-CD	EF-G_mRNA	EF-G_tRNA-P
**L34**	0	0	0	0	0	0	0	0	0	0	0	0	0	0	0	0	0	0
**L2**	0	0	0	0	0	0	0	0	0	0	0	0	0	0	0	0	0	0
**L9**	0	0	0	0	0	0	0	0	0	0	0	0	0	0	0	0	0	0
**L23**	0	0	0	0	0	0	0	0	0	0	0	0	0	0	0	0	0	0
**L33**	0	0	0	0	0	0	0	0	0	0	0	0	0	0	0	0	0	0
**L29**	0	0	0	0	0	0	0	0	0	0	0	0	0	0	0	0	0	0
**L1**	0	0	0	0	0	0	0	0	0	0	0	0	0	0	0	0	0	0
**L24**	0	0	0	0	0	0	0	0	0	0	0	0	0	0	0	0	0	0
**23S-D0**	0	0	0	0	0	0	0	0	0	0	0	0	0	0	0	0	0	0
**L32**	0	0	0	0	0	0	0	0	0	0	0	0	0	0	0	0	0	0
**L20**	0	0	0	0	0	0	0	0	0	0	0	0	0	0	0	0	0	0
**L22**	0	0	0	0	0	0	0	0	0	0	0	0	0	0	0	0	0	0
**L14**	0	0	0	0	0	0	0	0	0	0	0	0	0	0	0	0	0	0
**L17**	0	0	0	0	0	0	0	0	0	0	0	0	0	0	0	0	0	0
**L19**	0	0	0	0	0	0	0	0	0	0	0	0	0	0	0	0	0	0
**L21**	0	0	0	0	0	0	0	0	0	0	0	0	0	0	0	0	0	0
**16S-5'**	0	0	0	0	0	0	0	0	0	0	0	0	0	0	0	0	0	0
**S8**	0	0	0	0	0	0	1	0	0	0	0	0	0	0	0	0	0	0
**S12**	0	0	0	0	0	0	0	0	0	0	0	0	0	0	0	0	0	0
**S5**	0	0	0	0	0	0	0	0	0	0	0	0	0	0	0	0	0	0
**S17**	0	0	0	0	0	0	0	0	0	0	0	0	0	0	0	0	0	0
**S4**	0	0	0	0	0	0	0	0	0	0	0	0	0	0	0	0	0	0
**S15**	0	0	0	0	0	0	0	0	0	0	0	0	0	0	0	0	0	0
**S2**	0	0	0	0	0	0	1	0	0	0	0	0	0	0	0	0	0	0
**S20**	0	0	0	0	0	0	0	0	0	0	0	0	0	0	0	0	0	0
**S16**	0	0	0	0	0	0	0	0	0	0	0	0	0	0	0	0	0	0

	S11_23S-D5	S11_S18	S3_16S-5'	S13_23S-D2	S3_S5	S13_L5	S6_S18	S13_Thx	S19_23S-D2	S7_S9	L28_23S-D5	L4_23S-D2	L15_23S-D2	L20_23S-D2	L35_23S-D5	L15_23S-D5	L28_23S-D1	L34_23S-D2
**23S-D2**	0	0	0	1	0	0	0	0	1	0	0	1	1	1	1	1	0	1
**23S-D5**	1	0	0	0	0	0	0	0	0	0	1	1	1	0	1	1	1	0
**23S-D6**	0	0	0	0	0	0	0	0	0	0	0	0	0	0	0	0	0	0
**EF-G**	0	0	0	0	0	0	0	0	0	0	0	0	0	0	0	0	0	0
**L3**	0	0	0	0	0	0	0	0	0	0	0	0	0	0	0	0	0	0
**5SrRNA**	0	0	0	0	0	0	0	0	0	0	0	0	0	0	0	0	0	0
**L13**	0	0	0	0	0	0	0	0	0	0	0	0	0	1	0	0	0	0
**L16**	0	0	0	0	0	0	0	0	0	0	0	0	0	0	0	0	0	0
**L27**	0	0	0	0	0	0	0	0	0	0	0	0	0	0	1	0	0	0
**L36**	0	0	0	0	0	0	0	0	0	0	0	0	0	0	0	0	0	0
**L6**	0	0	0	0	0	0	0	0	0	0	0	0	0	0	0	0	0	0
**uL11**	0	0	0	0	0	0	0	0	0	0	0	0	0	0	0	0	0	0
**L25**	0	0	0	0	0	0	0	0	0	0	0	0	0	0	0	0	0	0
**L5**	0	0	0	0	0	1	0	0	0	0	0	0	0	0	0	0	0	0
**L18**	0	0	0	0	0	0	0	0	0	0	0	0	0	0	0	0	0	0
**L30**	0	0	0	0	0	0	0	0	0	0	0	0	0	1	0	0	0	0
**uL10**	0	0	0	0	0	0	0	0	0	0	0	0	0	0	0	0	0	0
**bL12**	0	0	0	0	0	0	0	0	0	0	0	0	0	0	0	0	0	0
**16S-CD**	1	1	0	0	0	0	1	0	0	0	0	0	0	0	0	0	0	0
**16S-3'M**	0	0	1	0	1	0	0	1	0	1	0	0	0	0	0	0	0	0
**16S-3'm**	0	0	0	0	0	0	0	0	0	0	0	0	0	0	0	0	0	0
**S13**	0	0	0	1	0	1	0	1	1	0	0	0	0	0	0	0	0	0
**mRNA**	0	0	0	0	0	0	0	0	0	0	0	0	0	0	0	0	0	0
**S9**	0	0	0	0	0	0	0	0	0	1	0	0	0	0	0	0	0	0
**S19**	0	0	0	1	0	0	0	0	1	0	0	0	0	0	0	0	0	0
**S7**	0	0	0	0	0	0	0	0	0	1	0	0	0	0	0	0	0	0
**S3**	0	0	1	0	1	0	0	0	0	0	0	0	0	0	0	0	0	0
**L31**	0	0	0	0	0	1	0	0	0	0	0	0	0	0	0	0	0	0
**S11**	1	1	0	0	0	0	0	0	0	0	0	0	0	0	0	0	0	0
**S14**	0	0	0	0	0	0	0	0	0	0	0	0	0	0	0	0	0	0
**S10**	0	0	0	0	0	0	0	0	0	0	0	0	0	0	0	0	0	0
**S6**	0	0	0	0	0	0	1	0	0	0	0	0	0	0	0	0	0	0
**Thx**	0	0	0	0	0	0	0	1	0	0	0	0	0	0	0	0	0	0
**S18**	0	1	0	0	0	0	1	0	0	0	0	0	0	0	0	0	0	0
**23S-D1**	0	0	0	0	0	0	0	0	0	0	1	1	1	1	1	1	1	1
**tRNA-P**	0	0	0	0	0	0	0	0	0	0	1	0	0	0	1	1	1	0
**23S-D4**	0	0	0	0	0	0	0	0	0	0	1	0	0	0	0	0	1	1
**23S-D3**	0	0	0	0	0	0	0	0	0	0	1	0	0	0	0	0	1	1
**L15**	0	0	0	0	0	0	0	0	0	0	1	1	1	1	1	1	1	0
**L28**	0	0	0	0	0	0	0	0	0	0	1	0	0	0	1	1	1	0
**L35**	0	0	0	0	0	0	0	0	0	0	1	1	1	0	1	1	0	0
**L4**	0	0	0	0	0	0	0	0	0	0	0	1	1	1	0	0	0	1

	S11_23S-D5	S11_S18	S3_16S-5'	S13_23S-D2	S3_S5	S13_L5	S6_S18	S13_Thx	S19_23S-D2	S7_S9	L28_23S-D5	L4_23S-D2	L15_23S-D2	L20_23S-D2	L35_23S-D5	L15_23S-D5	L28_23S-D1	L34_23S-D2
**L34**	0	0	0	0	0	0	0	0	0	0	0	1	0	0	0	0	0	1
**L2**	0	0	0	0	0	0	0	0	0	0	1	0	0	0	0	0	0	0
**L9**	0	0	0	0	0	0	0	0	0	0	1	0	0	0	0	0	1	0
**L23**	0	0	0	0	0	0	0	0	0	0	0	0	0	0	0	0	0	0
**L33**	0	0	0	0	0	0	0	0	0	0	1	0	0	0	1	0	0	0
**L29**	0	0	0	0	0	0	0	0	0	0	0	0	0	0	0	0	0	0
**L1**	0	0	0	0	0	0	0	0	0	0	0	0	0	0	0	0	0	0
**L24**	0	0	0	0	0	0	0	0	0	0	0	0	0	0	0	0	0	0
**23S-D0**	0	0	0	0	0	0	0	0	0	0	0	1	1	1	0	1	0	0
**L32**	0	0	0	0	0	0	0	0	0	0	0	0	0	0	0	0	0	0
**L20**	0	0	0	0	0	0	0	0	0	0	0	1	1	1	0	0	0	0
**L22**	0	0	0	0	0	0	0	0	0	0	0	0	0	0	0	0	0	1
**L14**	0	0	0	0	0	0	0	0	0	0	0	0	0	0	0	0	0	0
**L17**	0	0	0	0	0	0	0	0	0	0	0	0	0	0	0	0	0	0
**L19**	0	0	0	0	0	0	0	0	0	0	0	0	0	0	0	0	0	0
**L21**	0	0	0	0	0	0	0	0	0	0	0	0	1	1	0	0	0	0
**16S-5'**	0	0	1	0	0	0	0	0	0	0	0	0	0	0	0	0	0	0
**S8**	0	0	0	0	0	0	0	0	0	0	0	0	0	0	0	0	0	0
**S12**	0	0	0	0	0	0	0	0	0	0	0	0	0	0	0	0	0	0
**S5**	0	0	0	0	1	0	0	0	0	0	0	0	0	0	0	0	0	0
**S17**	0	0	0	0	0	0	0	0	0	0	0	0	0	0	0	0	0	0
**S4**	0	0	0	0	0	0	0	0	0	0	0	0	0	0	0	0	0	0
**S15**	0	0	0	0	0	0	0	0	0	0	0	0	0	0	0	0	0	0
**S2**	0	0	0	0	0	0	0	0	0	0	0	0	0	0	0	0	0	0
**S20**	0	0	0	0	0	0	0	0	0	0	0	0	0	0	0	0	0	0
**S16**	0	0	0	0	0	0	0	0	0	0	0	0	0	0	0	0	0	0

	tRNA-P_23S-D5	L15_23S-D1	L2_23S-D5	L33_23S-D5	L2_23S-D4	L35_23S-D2	L34_23S-D3	L4_23S-D1	tRNA-P_23S-D1	L35_23S-D1	L34_23S-D1	EF-G_16S-3'm	L2_23S-D2	L23_23S-D1	L35_L15
**23S-D2**	0	1	1	1	1	1	1	1	0	1	1	0	1	0	1
**23S-D5**	1	1	1	1	1	1	0	0	1	1	0	0	1	0	1
**23S-D6**	0	0	0	0	0	0	0	0	0	0	0	0	0	0	0
**EF-G**	0	0	0	0	0	0	0	0	0	0	0	1	0	0	0
**L3**	0	0	0	0	0	0	0	0	0	0	0	0	0	0	0
**5SrRNA**	0	0	0	0	0	0	0	0	0	0	0	0	0	0	0
**L13**	0	0	0	0	0	0	0	0	0	0	0	0	0	0	0
**L16**	0	0	0	0	0	0	0	0	0	0	0	0	0	0	0
**L27**	0	0	0	0	0	0	0	0	0	0	0	0	0	0	0
**L36**	0	0	0	0	0	0	0	0	0	0	0	0	0	0	0
**L6**	0	0	0	0	0	0	0	0	0	0	0	0	0	0	0
**uL11**	0	0	0	0	0	0	0	0	0	0	0	0	0	0	0
**L25**	0	0	0	0	0	0	0	0	0	0	0	0	0	0	0
**L5**	0	0	0	0	0	0	0	0	0	0	0	0	0	0	0
**L18**	0	0	0	0	0	0	0	0	0	0	0	0	0	0	0
**L30**	0	0	0	0	0	0	0	0	0	0	0	0	0	0	0
**uL10**	0	0	0	0	0	0	0	0	0	0	0	0	0	0	0
**bL12**	0	0	0	0	0	0	0	0	0	0	0	0	0	0	0
**16S-CD**	0	0	0	0	1	0	0	0	0	0	0	0	0	0	0
**16S-3'M**	0	0	0	0	0	0	0	0	0	0	0	0	0	0	0
**16S-3'm**	0	0	0	0	0	0	0	0	0	0	0	1	0	0	0
**S13**	0	0	0	0	0	0	0	0	0	0	0	0	0	0	0
**mRNA**	0	0	0	0	0	0	0	0	0	0	0	0	0	0	0
**S9**	0	0	0	0	0	0	0	0	0	0	0	0	0	0	0
**S19**	0	0	0	0	0	0	0	0	0	0	0	0	0	0	0
**S7**	0	0	0	0	0	0	0	0	0	0	0	0	0	0	0
**S3**	0	0	0	0	0	0	0	0	0	0	0	0	0	0	0
**L31**	0	0	0	0	0	0	0	0	0	0	0	0	0	0	0
**S11**	0	0	0	0	0	0	0	0	0	0	0	0	0	0	0
**S14**	0	0	0	0	0	0	0	0	0	0	0	0	0	0	0
**S10**	0	0	0	0	0	0	0	0	0	0	0	0	0	0	0
**S6**	0	0	0	0	0	0	0	0	0	0	0	0	0	0	0
**Thx**	0	0	0	0	0	0	0	0	0	0	0	0	0	0	0
**S18**	0	0	0	0	0	0	0	0	0	0	0	0	0	0	0
**23S-D1**	1	1	0	0	0	1	1	1	1	1	1	0	0	1	1
**tRNA-P**	1	1	0	1	0	0	0	0	1	1	0	1	0	0	0
**23S-D4**	0	0	1	0	1	0	1	0	0	0	0	1	1	0	0
**23S-D3**	0	0	0	0	1	0	1	0	0	0	1	0	1	1	0
**L15**	1	1	0	0	0	1	0	0	1	1	0	0	0	0	1
**L28**	1	1	1	1	0	0	0	0	1	0	0	0	0	0	0
**L35**	1	1	0	1	0	1	0	0	1	1	0	0	0	0	1
**L4**	0	0	0	0	0	1	0	1	0	0	1	0	0	0	0

	tRNA-P_23S-D5	L15_23S-D1	L2_23S-D5	L33_23S-D5	L2_23S-D4	L35_23S-D2	L34_23S-D3	L4_23S-D1	tRNA-P_23S-D1	L35_23S-D1	L34_23S-D1	EF-G_16S-3'm	L2_23S-D2	L23_23S-D1	L35_L15
**L34**	0	0	0	0	0	0	1	1	0	0	1	0	0	1	0
**L2**	0	0	1	0	1	0	0	0	0	0	0	0	1	0	0
**L9**	0	0	1	0	0	0	0	0	0	0	0	0	0	0	0
**L23**	0	0	0	0	0	0	1	0	0	0	1	0	0	1	0
**L33**	1	0	0	1	0	0	0	0	0	0	0	0	0	0	0
**L29**	0	0	0	0	0	0	0	0	0	0	0	0	0	1	0
**L1**	0	0	0	0	0	0	0	0	0	0	0	0	0	0	0
**L24**	0	0	0	0	0	0	0	0	0	0	0	0	0	0	0
**23S-D0**	0	0	0	0	0	0	0	1	0	0	0	0	0	0	0
**L32**	0	0	0	0	0	0	0	0	0	0	0	0	0	0	0
**L20**	0	0	0	0	0	0	0	1	0	0	0	0	0	0	0
**L22**	0	0	0	0	0	0	0	0	0	0	0	0	0	0	0
**L14**	0	0	0	0	0	0	0	0	0	0	0	0	0	0	0
**L17**	0	0	0	0	0	0	0	0	0	0	0	0	0	0	0
**L19**	0	0	0	0	0	0	0	0	0	0	0	0	0	0	0
**L21**	0	0	0	0	0	0	0	0	0	0	0	0	0	0	0
**16S-5'**	0	0	0	0	0	0	0	0	0	0	0	0	0	0	0
**S8**	0	0	0	0	0	0	0	0	0	0	0	0	0	0	0
**S12**	0	0	0	0	0	0	0	0	0	0	0	1	0	0	0
**S5**	0	0	0	0	0	0	0	0	0	0	0	0	0	0	0
**S17**	0	0	0	0	0	0	0	0	0	0	0	0	0	0	0
**S4**	0	0	0	0	0	0	0	0	0	0	0	0	0	0	0
**S15**	0	0	0	0	0	0	0	0	0	0	0	0	0	0	0
**S2**	0	0	0	0	0	0	0	0	0	0	0	0	0	0	0
**S20**	0	0	0	0	0	0	0	0	0	0	0	0	0	0	0
**S16**	0	0	0	0	0	0	0	0	0	0	0	0	0	0	0

	L28_23S-D3	L28_23S-D4	L23_23S-D3	L34_L23	16S-CD_23S-D4	L29_L23	L28_tRNA-P	L34_23S-D4	L9_23S-D5	L2_16S-CD	L29_23S-D1	L2_23S-D3	L9_L28	L4_L20	EF-G_23S-D4	L9_L2	L4_L15	L9_23S-D1
**23S-D2**	0	0	0	0	0	0	0	1	0	0	0	1	0	1	0	0	1	0
**23S-D5**	1	1	0	0	0	0	1	0	1	0	0	0	1	0	0	1	0	0
**23S-D6**	0	0	0	0	0	0	0	0	0	0	0	0	0	0	0	0	0	0
**EF-G**	0	0	0	0	0	0	0	0	0	0	0	0	0	0	1	0	0	0
**L3**	0	0	0	0	0	0	0	0	0	0	0	0	0	0	0	0	0	0
**5SrRNA**	0	0	0	0	0	0	0	0	0	0	0	0	0	0	0	0	0	0
**L13**	0	0	0	0	0	0	0	0	0	0	0	0	0	0	0	0	0	0
**L16**	0	0	0	0	0	0	0	0	0	0	0	0	0	0	0	0	0	0
**L27**	0	0	0	0	0	0	0	0	0	0	0	0	0	0	0	0	0	0
**L36**	0	0	0	0	0	0	0	0	0	0	0	0	0	0	0	0	0	0
**L6**	0	0	0	0	0	0	0	0	0	0	0	0	0	0	0	0	0	0
**uL11**	0	0	0	0	0	0	0	0	0	0	0	0	0	0	0	0	0	0
**L25**	0	0	0	0	0	0	0	0	0	0	0	0	0	0	0	0	0	0
**L5**	0	0	0	0	0	0	0	0	0	0	0	0	0	0	0	0	0	0
**L18**	0	0	0	0	0	0	0	0	0	0	0	0	0	0	0	0	0	0
**L30**	0	0	0	0	0	0	0	0	0	0	0	0	0	0	0	0	0	0
**uL10**	0	0	0	0	0	0	0	0	0	0	0	0	0	0	0	0	0	0
**bL12**	0	0	0	0	0	0	0	0	0	0	0	0	0	0	0	0	0	0
**16S-CD**	0	0	0	0	1	0	0	0	0	1	0	0	0	0	0	0	0	0
**16S-3'M**	0	0	0	0	0	0	0	0	0	0	0	0	0	0	0	0	0	0
**16S-3'm**	0	0	0	0	1	0	0	0	0	0	0	0	0	0	1	0	0	0
**S13**	0	0	0	0	0	0	0	0	0	0	0	0	0	0	0	0	0	0
**mRNA**	0	0	0	0	0	0	0	0	0	0	0	0	0	0	0	0	0	0
**S9**	0	0	0	0	0	0	0	0	0	0	0	0	0	0	0	0	0	0
**S19**	0	0	0	0	0	0	0	0	0	0	0	0	0	0	0	0	0	0
**S7**	0	0	0	0	0	0	0	0	0	0	0	0	0	0	0	0	0	0
**S3**	0	0	0	0	0	0	0	0	0	0	0	0	0	0	0	0	0	0
**L31**	0	0	0	0	0	0	0	0	0	0	0	0	0	0	0	0	0	0
**S11**	0	0	0	0	0	0	0	0	0	0	0	0	0	0	0	0	0	0
**S14**	0	0	0	0	0	0	0	0	0	0	0	0	0	0	0	0	0	0
**S10**	0	0	0	0	0	0	0	0	0	0	0	0	0	0	0	0	0	0
**S6**	0	0	0	0	0	0	0	0	0	1	0	0	0	0	0	0	0	0
**Thx**	0	0	0	0	0	0	0	0	0	0	0	0	0	0	0	0	0	0
**S18**	0	0	0	0	0	0	0	0	0	0	0	0	0	0	0	0	0	0
**23S-D1**	1	1	1	1	0	1	1	0	0	0	1	0	1	1	0	0	0	1
**tRNA-P**	0	0	0	0	1	0	1	0	0	0	0	0	0	0	0	0	0	0
**23S-D4**	1	1	0	0	1	0	0	1	0	1	0	1	0	0	1	0	0	0
**23S-D3**	1	1	1	1	0	1	0	1	0	0	0	1	0	0	0	0	0	0
**L15**	0	0	0	0	0	0	0	0	0	0	0	0	0	0	0	0	1	0
**L28**	1	1	0	0	0	0	1	0	1	0	0	0	1	0	0	0	0	1
**L35**	0	0	0	0	0	0	0	0	0	0	0	0	0	0	0	0	0	0
**L4**	0	0	0	0	0	0	0	0	0	0	0	0	0	1	0	0	1	0

	L28_23S-D3	L28_23S-D4	L23_23S-D3	L34_L23	16S-CD_23S-D4	L29_L23	L28_tRNA-P	L34_23S-D4	L9_23S-D5	L2_16S-CD	L29_23S-D1	L2_23S-D3	L9_L28	L4_L20	EF-G_23S-D4	L9_L2	L4_L15	L9_23S-D1
**L34**	0	0	1	1	0	0	0	1	0	0	0	0	0	0	0	0	0	0
**L2**	0	0	0	0	0	0	0	0	1	1	0	1	0	0	0	1	0	0
**L9**	0	0	0	0	0	0	0	0	1	0	0	0	1	0	0	1	0	1
**L23**	0	0	1	1	0	1	0	0	0	0	1	0	0	0	0	0	0	0
**L33**	0	0	0	0	0	0	0	0	0	0	0	0	0	0	0	0	0	0
**L29**	0	0	1	0	0	1	0	0	0	0	1	0	0	0	0	0	0	0
**L1**	0	0	0	0	0	0	0	0	0	0	0	0	0	0	0	0	0	0
**L24**	0	0	0	0	0	0	0	0	0	0	1	0	0	0	0	0	0	0
**23S-D0**	0	0	0	0	0	0	0	0	0	0	0	0	0	0	0	0	0	0
**L32**	0	0	0	0	0	0	0	0	0	0	0	0	0	0	0	0	0	0
**L20**	0	0	0	0	0	0	0	0	0	0	0	0	0	1	0	0	0	0
**L22**	0	0	0	0	0	0	0	0	0	0	0	0	0	0	0	0	0	0
**L14**	0	0	0	0	0	0	0	0	0	0	0	0	0	0	0	0	0	0
**L17**	0	0	0	0	0	0	0	0	0	0	0	0	0	0	0	0	0	0
**L19**	0	0	0	0	0	0	0	0	0	0	0	0	0	0	0	0	0	0
**L21**	0	0	0	0	0	0	0	0	0	0	0	0	0	0	0	0	0	0
**16S-5'**	0	0	0	0	0	0	0	0	0	0	0	0	0	0	0	0	0	0
**S8**	0	0	0	0	0	0	0	0	0	0	0	0	0	0	0	0	0	0
**S12**	0	0	0	0	0	0	0	0	0	0	0	0	0	0	0	0	0	0
**S5**	0	0	0	0	0	0	0	0	0	0	0	0	0	0	0	0	0	0
**S17**	0	0	0	0	0	0	0	0	0	0	0	0	0	0	0	0	0	0
**S4**	0	0	0	0	0	0	0	0	0	0	0	0	0	0	0	0	0	0
**S15**	0	0	0	0	0	0	0	0	0	0	0	0	0	0	0	0	0	0
**S2**	0	0	0	0	0	0	0	0	0	0	0	0	0	0	0	0	0	0
**S20**	0	0	0	0	0	0	0	0	0	0	0	0	0	0	0	0	0	0
**S16**	0	0	0	0	0	0	0	0	0	0	0	0	0	0	0	0	0	0

	L24_23S-D1	L1_23S-D5	L33_L35	L1_tRNA-P	tRNA-P_23S-D4	L32_23S-D0	L20_23S-D1	L22_23S-D0	L3_23S-D6	L17_23S-D6	L21_23S-D2	L19_23S-D6	L22_23S-D2	L20_L21	L32_L22	L14_L19
23S-D2	0	0	0	0	0	1	1	1	0	0	1	0	1	1	1	0
23S-D5	0	1	1	1	0	1	0	0	1	0	0	0	0	0	0	0
23S-D6	0	0	0	0	0	0	0	0	1	1	0	1	0	0	1	1
EF-G	0	0	0	0	0	0	0	0	0	0	0	0	0	0	0	0
L3	0	0	0	0	0	1	0	1	1	1	0	1	1	0	0	1
5SrRNA	0	0	0	0	0	0	0	0	0	0	0	0	0	0	0	0
L13	0	0	0	0	0	0	1	0	0	0	1	0	0	1	0	0
L16	0	0	0	0	0	0	0	0	0	0	0	0	0	0	0	0
L27	0	0	0	0	0	0	0	0	0	0	0	0	0	0	0	0
L36	0	0	0	0	0	0	0	0	0	0	0	0	0	0	0	0
L6	0	0	0	0	0	0	0	0	0	0	0	0	0	0	0	0
uL11	0	0	0	0	0	0	0	0	0	0	0	0	0	0	0	0
L25	0	0	0	0	0	0	0	0	0	0	0	0	0	0	0	0
L5	0	0	0	0	0	0	0	0	0	0	0	0	0	0	0	0
L18	0	0	0	0	0	0	0	0	0	0	0	0	0	0	0	0
L30	0	0	0	0	0	0	0	0	0	0	0	0	0	0	0	0
uL10	0	0	0	0	0	0	0	0	0	0	0	0	0	0	0	0
bL12	0	0	0	0	0	0	0	0	0	0	0	0	0	0	0	0
16S-CD	0	0	0	0	1	0	0	0	0	0	0	0	0	0	0	0
16S-3'M	0	0	0	0	0	0	0	0	0	0	0	0	0	0	0	0
16S-3'm	0	0	0	0	0	0	0	0	0	0	0	1	0	0	0	1
S13	0	0	0	0	0	0	0	0	0	0	0	0	0	0	0	0
mRNA	0	0	0	0	0	0	0	0	0	0	0	0	0	0	0	0
S9	0	0	0	0	0	0	0	0	0	0	0	0	0	0	0	0
S19	0	0	0	0	0	0	0	0	0	0	0	0	0	0	0	0
S7	0	0	0	0	0	0	0	0	0	0	0	0	0	0	0	0
S3	0	0	0	0	0	0	0	0	0	0	0	0	0	0	0	0
L31	0	0	0	0	0	0	0	0	0	0	0	0	0	0	0	0
S11	0	0	0	0	0	0	0	0	0	0	0	0	0	0	0	0
S14	0	0	0	0	0	0	0	0	0	0	0	0	0	0	0	0
S10	0	0	0	0	0	0	0	0	0	0	0	0	0	0	0	0
S6	0	0	0	0	0	0	0	0	0	0	0	0	0	0	0	0
Thx	0	0	0	0	0	0	0	0	0	0	0	0	0	0	0	0
S18	0	0	0	0	0	0	0	0	0	0	0	0	0	0	0	0
23S-D1	1	0	0	0	0	1	1	1	0	0	1	0	0	1	1	0
tRNA-P	0	1	0	1	1	0	0	0	0	0	0	0	0	0	0	0
23S-D4	0	0	0	0	1	0	0	0	0	0	0	1	0	0	0	0
23S-D3	0	0	0	0	0	0	0	1	0	1	0	0	1	0	0	0
L15	0	0	0	0	0	0	0	0	0	0	1	0	0	1	0	0
L28	0	0	0	0	0	0	0	0	0	0	0	0	0	0	0	0
L35	0	0	1	0	0	0	0	0	0	0	0	0	0	0	0	0
L4	0	0	0	0	0	0	1	0	0	0	0	0	0	0	0	0

	L24_23S-D1	L1_23S-D5	L33_L35	L1_tRNA-P	tRNA-P_23S-D4	L32_23S-D0	L20_23S-D1	L22_23S-D0	L3_23S-D6	L17_23S-D6	L21_23S-D2	L19_23S-D6	L22_23S-D2	L20_L21	L32_L22	L14_L19
**L34**	0	0	0	0	0	0	0	0	0	0	0	0	1	0	0	0
**L2**	0	0	0	0	0	0	0	0	0	0	0	0	0	0	0	0
**L9**	0	0	0	0	0	0	0	0	0	0	0	0	0	0	0	0
**L23**	0	0	0	0	0	0	0	0	0	0	0	0	0	0	0	0
**L33**	0	0	1	0	0	0	0	0	0	0	0	0	0	0	0	0
**L29**	1	0	0	0	0	0	0	0	0	0	0	0	0	0	0	0
**L1**	0	1	0	1	0	0	0	0	0	0	0	0	0	0	0	0
**L24**	1	0	0	0	0	0	0	0	0	0	0	0	0	0	0	0
**23S-D0**	0	0	0	0	0	1	1	1	1	1	1	0	1	1	1	1
**L32**	0	0	0	0	0	1	1	1	1	1	0	0	1	0	1	0
**L20**	0	0	0	0	0	1	1	0	0	0	1	0	0	1	0	0
**L22**	0	0	0	0	0	1	1	1	0	1	0	0	1	0	1	0
**L14**	0	0	0	0	0	0	0	0	1	0	0	1	0	0	0	1
**L17**	0	0	0	0	0	1	0	1	1	1	0	1	0	0	1	0
**L19**	0	0	0	0	0	0	0	0	1	1	0	1	0	0	0	1
**L21**	0	0	0	0	0	0	1	0	0	0	1	0	0	1	0	0
**16S-5'**	0	0	0	0	0	0	0	0	0	0	0	0	0	0	0	1
**S8**	0	0	0	0	0	0	0	0	0	0	0	0	0	0	0	0
**S12**	0	0	0	0	0	0	0	0	0	0	0	0	0	0	0	0
**S5**	0	0	0	0	0	0	0	0	0	0	0	0	0	0	0	0
**S17**	0	0	0	0	0	0	0	0	0	0	0	0	0	0	0	0
**S4**	0	0	0	0	0	0	0	0	0	0	0	0	0	0	0	0
**S15**	0	0	0	0	0	0	0	0	0	0	0	0	0	0	0	0
**S2**	0	0	0	0	0	0	0	0	0	0	0	0	0	0	0	0
**S20**	0	0	0	0	0	0	0	0	0	0	0	0	0	0	0	0
**S16**	0	0	0	0	0	0	0	0	0	0	0	0	0	0	0	0

	L20_23S-D0	L17_23S-D0	L4_23S-D0	L17_L22	L17_23S-D3	L22_23S-D3	L22_23S-D1	L15_23S-D0	L21_23S-D0	L32_23S-D6	L15_L21	L19_16S-3'm	L13_L20	L13_L21	L14_23S-D5	L32_23S-D1	L32_L17
**23S-D2**	1	0	1	0	0	1	0	1	1	0	1	0	1	1	0	0	0
**23S-D5**	0	0	1	0	0	0	0	1	0	0	0	0	0	0	1	0	0
**23S-D6**	0	1	0	1	1	0	0	0	0	1	0	1	0	0	1	0	1
**EF-G**	0	0	0	0	0	0	0	0	0	0	0	0	0	0	0	0	0
**L3**	0	1	0	0	0	0	0	0	0	1	0	0	0	0	0	0	0
**5SrRNA**	0	0	0	0	0	0	0	0	0	0	0	0	0	0	0	0	0
**L13**	0	0	0	0	0	0	0	0	0	0	0	0	1	1	0	0	0
**L16**	0	0	0	0	0	0	0	0	0	0	0	0	0	0	0	0	0
**L27**	0	0	0	0	0	0	0	0	0	0	0	0	0	0	0	0	0
**L36**	0	0	0	0	0	0	0	0	0	0	0	0	0	0	0	0	0
**L6**	0	0	0	0	0	0	0	0	0	0	0	0	0	0	0	0	0
**uL11**	0	0	0	0	0	0	0	0	0	0	0	0	0	0	0	0	0
**L25**	0	0	0	0	0	0	0	0	0	0	0	0	0	0	0	0	0
**L5**	0	0	0	0	0	0	0	0	0	0	0	0	0	0	0	0	0
**L18**	0	0	0	0	0	0	0	0	0	0	0	0	0	0	0	0	0
**L30**	0	0	0	0	0	0	0	0	0	0	0	0	0	0	0	0	0
**uL10**	0	0	0	0	0	0	0	0	0	0	0	0	0	0	0	0	0
**bL12**	0	0	0	0	0	0	0	0	0	0	0	0	0	0	0	0	0
**16S-CD**	0	0	0	0	0	0	0	0	0	0	0	0	0	0	0	0	0
**16S-3'M**	0	0	0	0	0	0	0	0	0	0	0	0	0	0	0	0	0
**16S-3'm**	0	0	0	0	0	0	0	0	0	0	0	1	0	0	0	0	0
**S13**	0	0	0	0	0	0	0	0	0	0	0	0	0	0	0	0	0
**mRNA**	0	0	0	0	0	0	0	0	0	0	0	0	0	0	0	0	0
**S9**	0	0	0	0	0	0	0	0	0	0	0	0	0	0	0	0	0
**S19**	0	0	0	0	0	0	0	0	0	0	0	0	0	0	0	0	0
**S7**	0	0	0	0	0	0	0	0	0	0	0	0	0	0	0	0	0
**S3**	0	0	0	0	0	0	0	0	0	0	0	0	0	0	0	0	0
**L31**	0	0	0	0	0	0	0	0	0	0	0	0	0	0	0	0	0
**S11**	0	0	0	0	0	0	0	0	0	0	0	0	0	0	0	0	0
**S14**	0	0	0	0	0	0	0	0	0	0	0	0	0	0	0	0	0
**S10**	0	0	0	0	0	0	0	0	0	0	0	0	0	0	0	0	0
**S6**	0	0	0	0	0	0	0	0	0	0	0	0	0	0	0	0	0
**Thx**	0	0	0	0	0	0	0	0	0	0	0	0	0	0	0	0	0
**S18**	0	0	0	0	0	0	0	0	0	0	0	0	0	0	0	0	0
**23S-D1**	1	0	1	0	0	1	1	0	0	0	0	0	1	1	0	1	0
**tRNA-P**	0	0	0	0	0	0	0	0	0	0	0	0	0	0	0	0	0
**23S-D4**	0	0	0	0	0	0	0	0	0	0	0	1	0	0	1	0	0
**23S-D3**	0	1	0	1	1	1	1	0	0	0	0	0	0	0	0	0	1
**L15**	0	0	0	0	0	0	0	1	1	0	1	0	0	0	0	0	0
**L28**	0	0	0	0	0	0	0	0	0	0	0	0	0	0	0	0	0
**L35**	0	0	0	0	0	0	0	0	0	0	0	0	0	0	0	0	0
**L4**	1	0	1	0	0	0	0	0	0	0	0	0	0	0	0	0	0

	L20_23S-D0	L17_23S-D0	L4_23S-D0	L17_L22	L17_23S-D3	L22_23S-D3	L22_23S-D1	L15_23S-D0	L21_23S-D0	L32_23S-D6	L15_L21	L19_16S-3'm	L13_L20	L13_L21	L14_23S-D5	L32_23S-D1	L32_L17
**L34**	0	0	0	0	0	0	0	0	0	0	0	0	0	0	0	0	0
**L2**	0	0	0	0	0	0	0	0	0	0	0	0	0	0	0	0	0
**L9**	0	0	0	0	0	0	0	0	0	0	0	0	0	0	0	0	0
**L23**	0	0	0	0	0	0	0	0	0	0	0	0	0	0	0	0	0
**L33**	0	0	0	0	0	0	0	0	0	0	0	0	0	0	0	0	0
**L29**	0	0	0	0	0	0	0	0	0	0	0	0	0	0	0	0	0
**L1**	0	0	0	0	0	0	0	0	0	0	0	0	0	0	0	0	0
**L24**	0	0	0	0	0	0	0	0	0	0	0	0	0	0	0	0	0
**23S-D0**	1	1	1	1	1	1	1	1	1	0	1	0	0	0	1	1	0
**L32**	1	1	0	1	1	0	1	0	0	1	0	0	0	0	0	1	1
**L20**	1	0	1	0	0	0	1	0	1	0	1	0	1	1	0	1	0
**L22**	0	1	0	1	1	1	1	0	0	1	0	0	0	0	0	1	1
**L14**	0	0	0	0	0	0	0	0	0	0	0	1	0	0	1	0	0
**L17**	0	1	0	1	1	1	0	0	0	1	0	0	0	0	0	0	1
**L19**	0	0	0	0	0	0	0	0	0	0	0	1	0	0	0	0	0
**L21**	1	0	0	0	0	0	0	1	1	0	1	0	1	1	0	0	0
**16S-5'**	0	0	0	0	0	0	0	0	0	0	0	0	0	0	0	0	0
**S8**	0	0	0	0	0	0	0	0	0	0	0	0	0	0	0	0	0
**S12**	0	0	0	0	0	0	0	0	0	0	0	0	0	0	0	0	0
**S5**	0	0	0	0	0	0	0	0	0	0	0	0	0	0	0	0	0
**S17**	0	0	0	0	0	0	0	0	0	0	0	0	0	0	0	0	0
**S4**	0	0	0	0	0	0	0	0	0	0	0	0	0	0	0	0	0
**S15**	0	0	0	0	0	0	0	0	0	0	0	0	0	0	0	0	0
**S2**	0	0	0	0	0	0	0	0	0	0	0	0	0	0	0	0	0
**S20**	0	0	0	0	0	0	0	0	0	0	0	0	0	0	0	0	0
**S16**	0	0	0	0	0	0	0	0	0	0	0	0	0	0	0	0	0

	L32_L20	L3_L14	L3_L19	L14_23S-D4	L3_L17	L19_23S-D4	16S-3'm_23S-D6	L19_16S-5'	L14_16S-3'm	L14_16S-5'	EF-G_L14	S8_16S-CD	S12_16S-5'	S12_16S-CD	S4_16S-5'	S5_16S-5'	S12_16S-3'm
**23S-D2**	0	0	0	0	0	0	0	0	0	0	0	0	0	0	0	0	0
**23S-D5**	0	0	0	1	0	0	0	0	0	0	0	0	0	0	0	0	0
**23S-D6**	0	1	1	0	1	1	1	0	0	0	0	0	0	0	0	0	0
**EF-G**	0	0	0	0	0	0	0	0	0	0	1	0	1	0	0	0	1
**L3**	0	1	1	0	1	0	0	0	0	0	0	0	0	0	0	0	0
**5SrRNA**	0	0	0	0	0	0	0	0	0	0	0	0	0	0	0	0	0
**L13**	0	0	0	0	0	0	0	0	0	0	0	0	0	0	0	0	0
**L16**	0	0	0	0	0	0	0	0	0	0	0	0	0	0	0	0	0
**L27**	0	0	0	0	0	0	0	0	0	0	0	0	0	0	0	0	0
**L36**	0	0	0	0	0	0	0	0	0	0	0	0	0	0	0	0	0
**L6**	0	0	0	0	0	0	0	0	0	0	0	0	0	0	0	0	0
**uL11**	0	0	0	0	0	0	0	0	0	0	0	0	0	0	0	0	0
**L25**	0	0	0	0	0	0	0	0	0	0	0	0	0	0	0	0	0
**L5**	0	0	0	0	0	0	0	0	0	0	0	0	0	0	0	0	0
**L18**	0	0	0	0	0	0	0	0	0	0	0	0	0	0	0	0	0
**L30**	0	0	0	0	0	0	0	0	0	0	0	0	0	0	0	0	0
**uL10**	0	0	0	0	0	0	0	0	0	0	0	0	0	0	0	0	0
**bL12**	0	0	0	0	0	0	0	0	0	0	0	0	0	0	0	0	0
**16S-CD**	0	0	0	0	0	0	0	0	0	0	0	1	1	1	1	1	1
**16S-3'M**	0	0	0	0	0	0	0	0	0	0	0	0	0	0	0	1	0
**16S-3'm**	0	0	0	0	0	1	1	0	1	0	0	0	1	1	0	0	1
**S13**	0	0	0	0	0	0	0	0	0	0	0	0	0	0	0	0	0
**mRNA**	0	0	0	0	0	0	0	0	0	0	0	0	0	0	0	0	0
**S9**	0	0	0	0	0	0	0	0	0	0	0	0	0	0	0	0	0
**S19**	0	0	0	0	0	0	0	0	0	0	0	0	0	0	0	0	0
**S7**	0	0	0	0	0	0	0	0	0	0	0	0	0	0	0	0	0
**S3**	0	0	0	0	0	0	0	0	0	0	0	0	0	0	0	0	0
**L31**	0	0	0	0	0	0	0	0	0	0	0	0	0	0	0	0	0
**S11**	0	0	0	0	0	0	0	0	0	0	0	0	0	0	0	0	0
**S14**	0	0	0	0	0	0	0	0	0	0	0	0	0	0	0	0	0
**S10**	0	0	0	0	0	0	0	0	0	0	0	0	0	0	0	0	0
**S6**	0	0	0	0	0	0	0	0	0	0	0	0	0	0	0	0	0
**Thx**	0	0	0	0	0	0	0	0	0	0	0	0	0	0	0	0	0
**S18**	0	0	0	0	0	0	0	0	0	0	0	0	0	0	0	0	0
**23S-D1**	1	0	0	0	0	0	0	0	0	0	0	0	0	0	0	0	0
**tRNA-P**	0	0	0	0	0	0	0	0	0	0	0	0	0	0	0	0	0
**23S-D4**	0	0	0	1	0	1	0	0	1	0	0	0	0	0	0	0	1
**23S-D3**	0	0	0	0	0	0	0	0	0	0	0	0	0	0	0	0	0
**L15**	0	0	0	0	0	0	0	0	0	0	0	0	0	0	0	0	0
**L28**	0	0	0	0	0	0	0	0	0	0	0	0	0	0	0	0	0
**L35**	0	0	0	0	0	0	0	0	0	0	0	0	0	0	0	0	0
**L4**	0	0	0	0	0	0	0	0	0	0	0	0	0	0	0	0	0

	L32_L20	L3_L14	L3_L19	L14_23S-D4	L3_L17	L19_23S-D4	16S-3'm_23S-D6	L19_16S-5'	L14_16S-3'm	L14_16S-5'	EF-G_L14	S8_16S-CD	S12_16S-5'	S12_16S-CD	S4_16S-5'	S5_16S-5'	S12_16S-3'm
**L34**	0	0	0	0	0	0	0	0	0	0	0	0	0	0	0	0	0
**L2**	0	0	0	0	0	0	0	0	0	0	0	0	0	0	0	0	0
**L9**	0	0	0	0	0	0	0	0	0	0	0	0	0	0	0	0	0
**L23**	0	0	0	0	0	0	0	0	0	0	0	0	0	0	0	0	0
**L33**	0	0	0	0	0	0	0	0	0	0	0	0	0	0	0	0	0
**L29**	0	0	0	0	0	0	0	0	0	0	0	0	0	0	0	0	0
**L1**	0	0	0	0	0	0	0	0	0	0	0	0	0	0	0	0	0
**L24**	0	0	0	0	0	0	0	0	0	0	0	0	0	0	0	0	0
**23S-D0**	1	0	0	1	1	0	0	0	0	0	0	0	0	0	0	0	0
**L32**	1	0	0	0	0	0	0	0	0	0	0	0	0	0	0	0	0
**L20**	1	0	0	0	0	0	0	0	0	0	0	0	0	0	0	0	0
**L22**	0	0	0	0	0	0	0	0	0	0	0	0	0	0	0	0	0
**L14**	0	1	1	1	0	0	0	1	1	1	1	0	0	0	0	0	0
**L17**	0	0	0	0	1	0	0	0	0	0	0	0	0	0	0	0	0
**L19**	0	1	1	0	0	1	1	1	0	1	0	0	0	0	0	0	0
**L21**	0	0	0	0	0	0	0	0	0	0	0	0	0	0	0	0	0
**16S-5'**	0	0	0	0	0	0	0	1	0	1	0	0	1	1	1	1	1
**S8**	0	0	0	0	0	0	0	0	0	0	0	1	0	1	1	1	0
**S12**	0	0	0	0	0	0	0	0	0	0	0	1	1	1	1	0	1
**S5**	0	0	0	0	0	0	0	0	0	0	0	1	0	1	1	1	0
**S17**	0	0	0	0	0	0	0	0	0	0	0	1	1	1	0	0	0
**S4**	0	0	0	0	0	0	0	0	0	0	0	0	1	0	1	1	0
**S15**	0	0	0	0	0	0	0	0	0	0	0	1	0	0	0	0	0
**S2**	0	0	0	0	0	0	0	0	0	0	0	1	0	0	0	0	0
**S20**	0	0	0	0	0	0	0	0	0	0	0	0	0	0	0	0	0
**S16**	0	0	0	0	0	0	0	0	0	0	0	0	0	0	0	0	0

	S17_16S-CD	S17_16S-5'	S15_16S-CD	S8_S17	S5_16S-CD	S12_S17	S5_16S-3'M	S20_16S-5'	S2_16S-3'M	S5_S8	S8_S12	S8_16S-5'	S16_16S-5'	S4_S5	S2_16S-CD	S15_S17	16S-CD_23S-D2	S16_16S-CD
**23S-D2**	0	0	1	0	0	0	0	0	0	0	0	0	0	0	0	0	1	0
**23S-D5**	0	0	0	0	0	0	0	0	0	0	0	0	0	0	0	0	0	0
**23S-D6**	0	0	0	0	0	0	0	0	0	0	0	0	0	0	0	0	0	0
**EF-G**	0	0	0	0	0	0	0	0	0	0	0	0	0	0	0	0	0	0
**L3**	0	0	0	0	0	0	0	0	0	0	0	0	0	0	0	0	0	0
**5SrRNA**	0	0	0	0	0	0	0	0	0	0	0	0	0	0	0	0	0	0
**L13**	0	0	0	0	0	0	0	0	0	0	0	0	0	0	0	0	0	0
**L16**	0	0	0	0	0	0	0	0	0	0	0	0	0	0	0	0	0	0
**L27**	0	0	0	0	0	0	0	0	0	0	0	0	0	0	0	0	0	0
**L36**	0	0	0	0	0	0	0	0	0	0	0	0	0	0	0	0	0	0
**L6**	0	0	0	0	0	0	0	0	0	0	0	0	0	0	0	0	0	0
**uL11**	0	0	0	0	0	0	0	0	0	0	0	0	0	0	0	0	0	0
**L25**	0	0	0	0	0	0	0	0	0	0	0	0	0	0	0	0	0	0
**L5**	0	0	0	0	0	0	0	0	0	0	0	0	0	0	0	0	0	0
**L18**	0	0	0	0	0	0	0	0	0	0	0	0	0	0	0	0	0	0
**L30**	0	0	0	0	0	0	0	0	0	0	0	0	0	0	0	0	0	0
**uL10**	0	0	0	0	0	0	0	0	0	0	0	0	0	0	0	0	0	0
**bL12**	0	0	0	0	0	0	0	0	0	0	0	0	0	0	0	0	0	0
**16S-CD**	1	1	1	1	1	1	0	0	0	1	1	0	1	0	1	1	1	1
**16S-3'M**	0	0	0	0	0	0	1	0	1	0	0	0	0	0	0	0	0	0
**16S-3'm**	0	0	0	0	0	0	0	1	0	0	0	0	0	0	0	0	0	0
**S13**	0	0	0	0	0	0	0	0	0	0	0	0	0	0	0	0	0	0
**mRNA**	0	0	0	0	0	0	0	0	0	0	0	0	0	0	0	0	0	0
**S9**	0	0	0	0	0	0	0	0	0	0	0	0	0	0	0	0	0	0
**S19**	0	0	0	0	0	0	0	0	0	0	0	0	0	0	0	0	0	0
**S7**	0	0	0	0	0	0	0	0	0	0	0	0	0	0	0	0	0	0
**S3**	0	0	0	0	0	0	1	0	1	0	0	0	0	0	0	0	0	0
**L31**	0	0	0	0	0	0	0	0	0	0	0	0	0	0	0	0	0	0
**S11**	0	0	0	0	0	0	0	0	0	0	0	0	0	0	0	0	0	0
**S14**	0	0	0	0	0	0	0	0	0	0	0	0	0	0	0	0	0	0
**S10**	0	0	0	0	0	0	0	0	0	0	0	0	0	0	0	0	0	0
**S6**	0	0	1	0	0	0	0	0	0	0	0	0	0	0	0	0	0	0
**Thx**	0	0	0	0	0	0	0	0	0	0	0	0	0	0	0	0	0	0
**S18**	0	0	0	0	0	0	0	0	0	0	0	0	0	0	0	0	0	0
**23S-D1**	0	0	0	0	0	0	0	0	0	0	0	0	0	0	0	0	0	0
**tRNA-P**	0	0	0	0	0	0	0	0	0	0	0	0	0	0	0	0	0	0
**23S-D4**	0	0	0	0	0	0	0	0	0	0	0	0	0	0	0	0	0	0
**23S-D3**	0	0	0	0	0	0	0	0	0	0	0	0	0	0	0	0	0	0
**L15**	0	0	0	0	0	0	0	0	0	0	0	0	0	0	0	0	0	0
**L28**	0	0	0	0	0	0	0	0	0	0	0	0	0	0	0	0	0	0
**L35**	0	0	0	0	0	0	0	0	0	0	0	0	0	0	0	0	0	0
**L4**	0	0	0	0	0	0	0	0	0	0	0	0	0	0	0	0	0	0

	S17_16S-CD	S17_16S-5'	S15_16S-CD	S8_S17	S5_16S-CD	S12_S17	S5_16S-3'M	S20_16S-5'	S2_16S-3'M	S5_S8	S8_S12	S8_16S-5'	S16_16S-5'	S4_S5	S2_16S-CD	S15_S17	16S-CD_23S-D2	S16_16S-CD
**L34**	0	0	0	0	0	0	0	0	0	0	0	0	0	0	0	0	0	0
**L2**	0	0	0	0	0	0	0	0	0	0	0	0	0	0	0	0	0	0
**L9**	0	0	0	0	0	0	0	0	0	0	0	0	0	0	0	0	0	0
**L23**	0	0	0	0	0	0	0	0	0	0	0	0	0	0	0	0	0	0
**L33**	0	0	0	0	0	0	0	0	0	0	0	0	0	0	0	0	0	0
**L29**	0	0	0	0	0	0	0	0	0	0	0	0	0	0	0	0	0	0
**L1**	0	0	0	0	0	0	0	0	0	0	0	0	0	0	0	0	0	0
**L24**	0	0	0	0	0	0	0	0	0	0	0	0	0	0	0	0	0	0
**23S-D0**	0	0	0	0	0	0	0	0	0	0	0	0	0	0	0	0	0	0
**L32**	0	0	0	0	0	0	0	0	0	0	0	0	0	0	0	0	0	0
**L20**	0	0	0	0	0	0	0	0	0	0	0	0	0	0	0	0	0	0
**L22**	0	0	0	0	0	0	0	0	0	0	0	0	0	0	0	0	0	0
**L14**	0	0	0	0	0	0	0	0	0	0	0	0	0	0	0	0	0	0
**L17**	0	0	0	0	0	0	0	0	0	0	0	0	0	0	0	0	0	0
**L19**	0	0	0	0	0	0	0	0	0	0	0	0	0	0	0	0	0	0
**L21**	0	0	0	0	0	0	0	0	0	0	0	0	0	0	0	0	0	0
**16S-5'**	1	1	0	1	1	1	1	1	0	1	0	1	1	1	0	0	0	1
**S8**	1	1	1	1	1	1	0	0	1	1	1	1	0	0	1	0	0	0
**S12**	1	1	0	1	1	1	0	0	0	0	1	0	0	0	0	0	0	0
**S5**	0	0	0	0	1	0	1	0	1	1	0	1	0	1	0	0	0	0
**S17**	1	1	1	1	0	1	0	1	0	0	1	0	0	0	0	1	0	0
**S4**	0	0	0	0	0	0	0	0	0	0	0	1	0	1	0	0	0	0
**S15**	1	0	1	0	0	0	0	0	0	0	0	0	0	0	0	1	1	0
**S2**	0	0	0	0	0	0	1	0	1	0	0	0	0	0	1	0	0	0
**S20**	0	1	0	0	0	0	0	1	0	0	0	0	1	0	0	0	0	0
**S16**	0	0	0	0	0	0	0	1	0	0	0	0	1	0	0	0	0	1

	S20_16S-3'm	EF-G_16S-5'	S12_EF-G	S4_16S-CD	S15_23S-D2	S3_S4
**23S-D2**	0	0	0	0	1	0
**23S-D5**	0	0	0	0	0	0
**23S-D6**	0	0	0	0	0	0
**EF-G**	0	1	1	0	0	0
**L3**	0	0	0	0	0	0
**5SrRNA**	0	0	0	0	0	0
**L13**	0	0	0	0	0	0
**L16**	0	0	0	0	0	0
**L27**	0	0	0	0	0	0
**L36**	0	0	0	0	0	0
**L6**	0	0	0	0	0	0
**uL11**	0	0	0	0	0	0
**L25**	0	0	0	0	0	0
**L5**	0	0	0	0	0	0
**L18**	0	0	0	0	0	0
**L30**	0	0	0	0	0	0
**uL10**	0	0	0	0	0	0
**bL12**	0	0	0	0	0	0
**16S-CD**	0	0	0	1	1	0
**16S-3'M**	0	0	0	0	0	0
**16S-3'm**	1	0	0	0	0	0
**S13**	0	0	0	0	0	0
**mRNA**	0	0	0	0	0	0
**S9**	0	0	0	0	0	0
**S19**	0	0	0	0	0	0
**S7**	0	0	0	0	0	0
**S3**	0	0	0	0	0	1
**L31**	0	0	0	0	0	0
**S11**	0	0	0	0	0	0
**S14**	0	0	0	0	0	0
**S10**	0	0	0	0	0	0
**S6**	0	0	0	0	0	0
**Thx**	0	0	0	0	0	0
**S18**	0	0	0	0	0	0
**23S-D1**	0	0	0	0	0	0
**tRNA-P**	0	0	0	0	0	0
**23S-D4**	0	0	0	0	0	0
**23S-D3**	0	0	0	0	0	0
**L15**	0	0	0	0	0	0
**L28**	0	0	0	0	0	0
**L35**	0	0	0	0	0	0
**L4**	0	0	0	0	0	0

	S20_16S-3'm	EF-G_16S-5'	S12_EF-G	S4_16S-CD	S15_23S-D2	S3_S4
**L34**	0	0	0	0	0	0
**L2**	0	0	0	0	0	0
**L9**	0	0	0	0	0	0
**L23**	0	0	0	0	0	0
**L33**	0	0	0	0	0	0
**L29**	0	0	0	0	0	0
**L1**	0	0	0	0	0	0
**L24**	0	0	0	0	0	0
**23S-D0**	0	0	0	0	0	0
**L32**	0	0	0	0	0	0
**L20**	0	0	0	0	0	0
**L22**	0	0	0	0	0	0
**L14**	0	0	0	0	0	0
**L17**	0	0	0	0	0	0
**L19**	0	0	0	0	0	0
**L21**	0	0	0	0	0	0
**16S-5'**	1	1	1	1	0	0
**S8**	0	0	0	0	0	0
**S12**	0	1	1	0	0	0
**S5**	0	0	0	0	0	0
**S17**	0	0	0	0	0	0
**S4**	0	0	0	1	0	1
**S15**	0	0	0	0	1	0
**S2**	0	0	0	0	0	0
**S20**	1	0	0	0	0	0
**S16**	0	0	0	0	0	0
