# Supplementary material for: Modeling the ribosome as a bipartite graph
Source: PLoS One. 2022 Dec 30;17(12):e0279455. doi: 10.1371/journal.pone.0279455 (PMC9803165; doi:10.1371/journal.pone.0279455)
Supplement: S4 Table — Individual basic graph properties for (a) T. thermophilus pdb files and (b) S. cerevisiae and H. sapiens pdb files. (PDF) [file pone.0279455.s007.pdf]

Si Table 4a

	5ot7	1vy4	1vy5	1vy6	1vy7	4v5f	4v9j	4v9k	4v9m	4v9h	average
**Ref**	16	35	35	35	35	32	34	34	34	33	
**Method**	Cryo-EM	X_ray	X_ray	X_ray	X_ray	X_ray	X_ray	X_ray	X_ray	X_ray	
**Resolution**	3.80 Å	2.60 Å	2.55 Å	2.9 Å	2.80 Å	3.60 Å	3.86 Å	3.50 Å	4.00 Å	2.86 Å	3.3 Å
**Antibiotics present**	N/A	N/A	N/A	N/A	N/A	fusidic acid	Viomycin	Viomycin	Fusidic Acid	N/A	
**State**	post translocation with EF-G	post-attack of peptide bond	post-attack of peptide bond	pre-attack of peptide bond	pre-attack of peptide bond	post translocation with EF-G	intermediate translocation with EF-G	intermediate translocation with EF-G	intermediate translocation with EF-G	intermediate translocation with EF-G	
**tRNAs present**	pe/E	A,P,E	A,P,E	A,P,E	A,P,E	P,E	pe*/E	pe*/E	pe*/E	PE	
**Element size**	68	65	65	64	65	67	67	67	66	67	66.1
**Interaction size**	218	228	227	207	212	228	220	227	222	211	220
**Order**	1115	1143	1138	1019	1032	1166	1055	1111	1072	1036	1088.7
**density**	0.075	0.077	0.077	0.077	0.075	0.076	0.072	0.073	0.073	0.073	0.075
**Ave element degree**	16.4	17.58	17.51	15.92	15.88	17.4	15.75	16.58	16.24	15.46	16.472
**rRNA degree**	39.583	41.25	40.75	38	37.833	41.167	38.75	41	39.5	37.917	39.575
**rprotein degree**	10.698	10.898	10.796	10.531	10.571	11	10.137	10.314	10.333	10.154	10.543
**Average interaction degree**	5.11	5.01	5.01	4.92	4.87	5.11	4.8	4.89	4.83	4.91	4.946
**Average degree**	7.8	7.8	7.79	7.52	7.45	7.91	7.35	7.56	7.44	7.45	7.607
**Average path length**	3.43	3.44	3.49	3.5	3.58	3.37	3.51	3.48	3.48	3.51	3.479
**diameter**	8	8	8	8	8	8	8	8	8	8	8
**Dot clustering**	0.19	0.19	0.19	0.198	0.198	0.183	0.198	0.195	0.193	0.197	0.193
**Dot clustering element**	0.143	0.139	0.139	0.149	0.151	0.137	0.15	0.146	0.141	0.154	0.145
**Dot clustering interactions**	0.205	0.204	0.204	0.214	0.212	0.196	0.212	0.21	0.208	0.211	0.208
**rRNA clustering**	0.13	0.126	0.126	0.131	0.126	0.126	0.13	0.13	0.124	0.132	0.128
**rprotein clustering**	0.147	0.143	0.142	0.154	0.157	0.14	0.156	0.151	0.147	0.16	0.15
**Redundancy**	0.928	0.941	0.935	0.938	0.928	0.903	0.922	0.925	0.912	0.903	0.924
**Element redundancy**	0.85	0.847	0.845	0.855	0.846	0.797	0.801	0.817	0.802	0.817	0.828
**rRNA redundancy**	0.594	0.59	0.574	0.576	0.558	0.597	0.567	0.603	0.579	0.596	0.583
**rprotein redundancy**	0.924	0.928	0.927	0.928	0.919	0.858	0.869	0.876	0.87	0.883	0.898
**Interaction redundancy**	0.952	0.968	0.961	0.963	0.953	0.934	0.959	0.957	0.944	0.93	0.952
**Degree centralization**	0.298	0.262	0.255	0.283	0.271	0.312	0.317	0.303	0.298	0.299	0.29
**Max degree node**	23S-D2	23S-D2	23S-D2	23S-D2	23S-D2	23S-D2	23S-D2	23S-D2	23S-D2	23S-D2	23S-D2: 10
**Betweenness centralization**	7.00E-06	6.00E-06	5.00E-06	8.00E-06	7.00E-06	7.00E-06	8.00E-06	7.00E-06	7.00E-06	8.00E-06	6.90E-06
**Max betweenness node**	23S-D2	23S-D2	23S-D2	23S-D2	23S-D2	23S-D2	23S-D2	23S-D2	23S-D2	23S-D2	23S-D2: 10
**Closeness centralization**	0.365	0.377	0.389	0.386	0.398	0.378	0.4	0.384	0.37	0.389	0.384
**Max closeness node**	uL2_23S-D4	uL2_23S-D4	uL2_23S-D4	uL2_23S-D4	uL2_23S-D4	uL2_23S-D4	uL2_23S-D4	uL2_23S-D4	uL2_23S-D4	uL2_23S-D4	uL2_23S-D4: 10
**NODF**	0.126	0.126	0.125	0.123	0.121	0.129	0.117	0.122	0.12	0.121	0.123
**modularity**	0.486	0.483	0.488	0.52	0.508	0.488	0.519	0.517	0.529	0.508	0.505

SI Table 4b

	S. cerevisaie				H. sapiens				average eukaryote
	6t7i	6t4q	6t7t	average	6y57	6y0g	6y2l	average	
Ref	17	17	17	-	36	36	36	-	-
Method	cryo-EM	cryo-EM	cryo-EM	-	cryo-EM	cryo-EM	cryo-EM	-	-
Resolution	3.20 Å	2.60 Å	3.10 Å	-	3.50 Å	3.20 Å	3. Å	-	
State	stalled post translocation state	stalled post translocation state	stalled post translocation state	-	intermediate translocation	pre translocation	post translocation	-	-
tRNAs present	P,E	P,E	P,E	-	A/P,P/E	A,P	P	-	-
Element size	90	90	91	90.333	91	92	89	90.667	90.5
Interaction size	377	380	381	379.333	375	395	380	383.333	381.333
Order	2211	2214	2236	2220.333	2051	2210	2068	2109.667	2165
density	0.065	0.065	0.064	0.065	0.06	0.061	0.061	0.061	0.063
Ave element degree	24.57	24.6	24.57	24.58	22.54	24.02	23.24	23.267	23.9235
rRNA degree	64.846	65.308	65.692	65.282	64	68	64.308	65.436	65.359
rprotein degree	17.37	17.274	17.521	17.388	15.432	16.493	16.208	16.044	16.716
Average interaction degree	5.86	5.83	5.87	5.853	5.47	5.59	5.44	5.5	5.6765
Average degree	9.47	9.42	9.47	9.453	8.8	9.08	8.82	8.9	9.1765
Average path length	3.48	3.47	3.49	3.48	3.61	3.49	3.49	3.53	3.505
diameter	8	8	8	8	8	8	8	8	8
Dot clustering	0.17	0.169	0.17	0.17	0.176	0.171	0.173	0.173	0.1715
Dot clustering element	0.123	0.122	0.12	0.122	0.122	0.117	0.117	0.119	0.1205
Dot clustering interactions	0.182	0.181	0.182	0.182	0.189	0.183	0.186	0.186	0.184
rRNA clustering	0.108	0.107	0.108	0.108	0.105	0.102	0.103	0.103	0.1055
rprotein clustering	0.125	0.125	0.124	0.125	0.126	0.121	0.121	0.123	0.124
Redundancy	0.949	0.949	0.947	0.948	0.948	0.951	0.944	0.948	0.948
Element redundancy	0.859	0.854	0.857	0.857	0.867	0.863	0.862	0.864	0.8605
rRNA redundancy	0.641	0.636	0.645	0.641	0.623	0.622	0.621	0.622	0.6315
rprotein redundancy	0.906	0.901	0.901	0.903	0.919	0.911	0.911	0.914	0.9085
Interaction redundancy	0.97	0.971	0.969	0.97	0.967	0.972	0.964	0.968	0.969
Degree centralization	0.276	0.273	0.278	0.276	0.296	0.292	0.303	0.297	0.2865
Max degree node	25S-D2	25S-D2	25S-D2	25S-D2: 3	28S-D2	28S-D2	28S-D2	28S-D2: 3	28S-D2: 6
Betweenness centralization	3.00E-06	3.00E-06	3.00E-06	0.000003	2.00E-06	3.00E-06	3.00E-06	0.00000267	0.000002835
Max betweenness node	25S-D2	25S-D2	25S-D2	25S-D2: 3	28S-D2	28S-D2	28S-D2	28S-D2: 3	25S/28S-D2: 6
Closeness centralization	0.401	0.4	0.405	0.402	0.43	0.405	0.417	0.417	0.4095
Max closeness node	uL2__25S-D4	uL2_25S-D4	uL2_25S-D4	uL2_25S-D4: 3	uL2_28S-D4	uL2_eL37	uL2_28S-D4	uL2_28S-D4: 2, uL2_L37a: 1	uL2_25S/28S-D4: 5, uL2_eL37: 1
NODF	0.117	0.116	0.117	0.117	0.114	0.113	0.117	0.115	0.116
modularity	0.531	0.51	0.521	0.521	0.545	0.526	0.528	0.533	0.527
