# Supplementary material for: Modeling the ribosome as a bipartite graph
Source: PLoS One. 2022 Dec 30;17(12):e0279455. doi: 10.1371/journal.pone.0279455 (PMC9803165; doi:10.1371/journal.pone.0279455)
Supplement: S5 Table — Individual basic graph properties of unipartite graphs for (a) T. thermophilus pdb files and (b) S. cerevisiae and H. sapiens pdb files. (PDF) [file pone.0279455.s008.pdf]

SI Table 5a

	5ot7			1vy4			1vy5			1vy6		
	**dyadic**	**elem**	**inter**	**dyadic**	**elem**	**inter**	**dyadic**	**elem**	**inter**	**dyadic**	**elem**	**inter**
**Size**	68	68	218	65	65	228	65	65	227	64	64	207
**Order**	219	542	9108	229	511	9813	228	516	9658	208	465	7917
**Average degree**	3.22	7.97	41.78	3.52	7.86	43.04	3.51	7.94	42.55	3.25	7.27	38.25
**Average rRNA degree**	13.25	27.667	0	13.417	27	0	13.417	27	0	12.667	25.5	0
**Average rprotein degree**	4.692	12.962	0	5.122	12.061	0	5.082	12.163	0	4.959	11.551	0
**Max degree**	26	45	160	24	41	167	24	37	166	23	40	150
**Diamater**	4	4	3	5	4	3	5	4	3	6	4	3
**Average path length**	2.51	1.9	1.66	2.51	1.91	1.66	2.51	1.93	1.7	2.58	1.95	1.69
**Cluster coefficient**	0.484	0.692	0.759	0.453	0.699	0.755	0.446	0.705	0.755	0.44	0.699	0.772
**rRNA cluster coefficient**	0.198	0.471	0	0.203	0.463	0	0.202	0.47	0	0.195	0.453	0
**rprotein cluster coefficient**	0.549	0.748	0	0.522	0.772	0	0.514	0.778	0	0.514	0.762	0
**Density**	0.096	0.238	0.385	0.11	0.246	0.379	0.11	0.248	0.377	0.103	0.231	0.371
**Assortativity**	-0.285	-0.106	0.298	-0.263	-0.117	0.167	-0.265	-0.094	0.155	-0.305	-0.125	0.313
**Degree centralization**	0.301	0.447	0.356	0.273	0.407	0.36	0.274	0.341	0.361	0.27	0.417	0.36
**Max degree node**	23S-D2	23S-D2	uL2_23S-D4	23S-D2	23S-D2	uL2_23S-D4	23S-D2	23S-D5	uL2_23S-D4	23S-D2	23S-D2	uL2_23S-D4
**Betweenness centralization**	0.000122	6.60E-05	2.00E-06	0.000119	7.70E-05	2.00E-06	0.000119	5.50E-05	1.00E-06	0.000131	9.40E-05	3.00E-06
**Max betweenness node**	23S-D2	23S-D2	S13_S19	23S-D2	23S-D2	S13_S19	23S-D2	23S-D2	uL2_23S-D4	23S-D2	23S-D2	S13_S19
**Closeness Centralization**	0.343	0.447	0.363	0.328	0.401	0.37	0.329	0.36	0.388	0.322	0.434	0.374
**Max closeness node**	23S-D2	23S-D2	uL2_23S-D4	23S-D2	23S-D2	uL2_23S-D4	23S-D2	23S-D5	uL2_23S-D4	23S-D2	23S-D2	uL2_23S-D4
**Modularity**	0.413	0.314	0.271	0.395	0.345	0.327	0.411	0.351	0.328	0.435	0.371	0.319

	1vy7			4v5f			4v9j			4v9k		
	**dyadic**	**elem**	**inter**	**dyadic**	**elem**	**inter**	**dyadic**	**elem**	**inter**	**dyadic**	**elem**	**inter**
**Size**	65	65	212	67	67	228	68	68	220	68	68	227
**Order**	215	476	8121	234	544	10346	224	484	8773	234	499	9468
**Average degree**	3.31	7.32	38.31	3.49	8.12	45.38	3.29	7.12	39.88	3.44	7.34	41.71
**Average rRNA degree**	12.917	25.667	0	13.667	27.917	0	13.667	25.667	0	13.917	27.333	0
**Average rprotein degree**	5.061	11.714	0	5.08	12.64	0	4.824	11.255	0	5.059	11.02	0
**Max degree**	23	38	152	26	49	170	27	44	163	28	45	166
**Diamater**	6	4	4	5	4	3	5	4	4	5	4	3
**Average path length**	2.57	1.98	1.74	2.43	1.87	1.64	2.55	1.98	1.69	2.51	1.95	1.68
**Cluster coefficient**	0.455	0.693	0.767	0.454	0.686	0.745	0.447	0.689	0.75	0.468	0.705	0.756
**rRNA cluster coefficient**	0.203	0.438	0	0.205	0.464	0	0.195	0.44	0	0.21	0.441	0
**rprotein cluster coefficient**	0.511	0.755	0	0.513	0.748	0	0.501	0.751	0	0.525	0.768	0
**Density**	0.103	0.229	0.363	0.106	0.246	0.4	0.098	0.212	0.364	0.103	0.219	0.369
**Assortativity**	-0.29	-0.116	0.343	-0.256	-0.112	0.228	-0.273	-0.135	0.362	-0.269	-0.16	0.263
**Degree centralization**	0.264	0.376	0.361	0.297	0.512	0.352	0.314	0.458	0.384	0.325	0.466	0.369
**Max degree node**	23S-D2	23S-D2	uL2_23S-D4	23S-D2	23S-D2	uL2_23S-D4	23S-D2	23S-D2	uL2_23S-D4	23S-D2	23S-D2	uL2_23S-D4
**Betweenness centralization**	0.000123	7.00E-05	2.00E-06	0.000125	8.80E-05	1.00E-06	0.000129	9.00E-05	2.00E-06	0.000124	9.20E-05	1.00E-06
**Max betweenness node**	23S-D2	23S-D2	uL2_23S-D4	23S-D2	23S-D2	uL2_23S-D4	23S-D2	23S-D2	S13_S19	23S-D2	23S-D2	S13_S19
**Closeness Centralization**	0.327	0.407	0.396	0.371	0.514	0.361	0.354	0.472	0.393	0.351	0.458	0.378
**Max closeness node**	23S-D2	23S-D2	uL2_23S-D4	23S-D2	23S-D2	uL2_23S-D4	23S-D2	23S-D2	uL2_23S-D4	23S-D2	23S-D2	uL2_23S-D4
**Modularity**	0.438	0.376	0.323	0.397	0.324	0.257	0.426	0.385	0.249	0.434	0.35	0.305

	4v9m			4v9h		
	**dyadic**	**elem**	**inter**	**dyadic**	**elem**	**inter**
**Size**	66	66	222	67	67	211
**Order**	227	500	9005	217	478	8141
**Average degree**	3.44	7.58	40.56	3.24	7.13	38.58
**Average rRNA degree**	13.667	27.083	0	12.917	26	0
**Average rprotein degree**	4.98	11.627	0	4.808	11.442	0
**Max degree**	28	44	159	25	42	156
**Diamater**	5	4	3	5	4	3
**Average path length**	2.47	1.92	1.69	2.56	1.98	1.68
**Cluster coefficient**	0.446	0.685	0.75	0.457	0.685	0.765
**rRNA cluster coefficient**	0.21	0.451	0	0.193	0.448	0
**rprotein cluster coefficient**	0.512	0.753	0	0.527	0.746	0
**Density**	0.106	0.233	0.367	0.098	0.216	0.367
**Assortativity**	-0.277	-0.13	0.32	-0.276	-0.069	0.351
**Degree centralization**	0.335	0.458	0.356	0.289	0.433	0.379
**Max degree node**	23S-D2	23S-D2	uL2_23S-D4	23S-D2	23S-D2	uL2_23S-D4
**Betweenness centralization**	0.00013	8.50E-05	2.00E-06	0.000123	8.90E-05	2.00E-06
**Max betweenness node**	23S-D2	23S-D2	S13_S19	23S-D2	23S-D2	S13_S19
**Closeness Centralization**	0.344	0.465	0.361	0.34	0.449	0.388
**Max closeness node**	23S-D2	23S-D2	uL2_23S-D4	23S-D2	23S-D2	uL2_23S-D4
**Modularity**	0.408	0.339	0.284	0.458	0.38	0.213

SI Table 5b

	S. cerevisaie											
	6t7i			6t4q			6t7t			Average		
	**dyadic**	**element**	**interaction**	**dyadic**	**element**	**interaction**	**dyadic**	**element**	**interaction**	**dyadic**	**element**	**interaction**
**Size**	90	90	377	90	90	380	91	91	381	90.333	90.333	379.333
**Order**	384	1051	25819	388	1056	26049	391	1066	26226	387.667	1057.667	26031.333
**Average degree**	4.27	11.68	68.49	4.31	11.73	68.55	4.3	11.71	68.83	4.293	11.707	68.623
**Average rRNA degree**	18.769	40.769	0	19	40.846	0	19.231	40.846	0	19	40.82	0
**Average rprotein degree**	6.685	20	0	6.685	20.014	0	6.74	20.11	0	6.703	20.041	0
**Max degree**	34	65	282	35	65	285	35	64	286	34.667	64.667	284.333
**Diameter**	6	3	4	6	3	4	6	3	4	6	3	4
**Average path length**	2.57	1.88	1.7	2.56	1.87	1.7	2.55	1.89	1.7	2.56	1.88	1.7
**Cluster coefficient**	0.496	0.706	0.779	0.498	0.702	0.774	0.5	0.707	0.779	0.498	0.705	0.777
**rRNA cluster coefficient**	0.229	0.501	0	0.233	0.496	0	0.229	0.498	0	0.23	0.498	0
**rprotein cluster coefficient**	0.543	0.749	0	0.548	0.745	0	0.548	0.749	0	0.546	0.748	0
**Density**	0.096	0.262	0.364	0.097	0.264	0.362	0.095	0.26	0.362	0.096	0.262	0.363
**Assortativity**	-0.237	-0.001	0.275	-0.235	-0.013	0.245	-0.242	-0.012	0.281	-0.238	-0.009	0.267
**Degree centralization**	0.293	0.479	0.388	0.303	0.477	0.392	0.3	0.461	0.392	0.299	0.472	0.391
**Max degree node**	25S-D2	25S-D2	uL2_25S-D4	25S-D2	25S-D2	uL2_25S-D4	25S-D2	25S-D2	uL2_25S-D4	25S-D2	25S-D2	uL2_25S-D4
**Betweenness centralization**	6.20E-05	3.80E-05	1.00E-06	6.40E-05	3.60E-05	1.00E-06	6.10E-05	3.40E-05	1.00E-06	6.23E-05	6.23E-05	6.23E-05
**Max betweenness node**	25S-D2	25S-D2	uS19_uS13	25S-D2	25S-D2	uS19_uS13	25S-D2	25S-D2	uS19_uS13	25S-D2	25S-D2	uS19_uS13
**Closeness Centralization**	0.348	0.507	0.403	0.353	0.5	0.403	0.34	0.485	0.407	0.347	0.497	0.404
**Max closeness node**	25S-D2	25S-D2	uL2_25S-D4	25S-D2	25S-D2	uL2_25S-D4	25S-D2	25S-D2	uL2_25S-D4	25S-D2	25S-D2	uL2_25S-D4
**Modularity**	0.442	0.346	0.322	0.431	0.343	0.327	0.425	0.348	0.324	0.433	0.346	0.324

	H. Sapiens											
	6y57			6y0g			6y2l			Average		
	**dyadic**	**element**	**interaction**	**dyadic**	**element**	**interaction**	**dyadic**	**element**	**interaction**	**dyadic**	**element**	**interaction**
**Size**	91	91	375	92	92	395	89	89	380	90.667	90.667	383.333
**Order**	381	960	25095	402	1015	28308	385	948	26274	389.333	974.333	26559
**Average degree**	4.19	10.55	66.92	4.37	11.03	71.67	4.33	10.65	69.14	4.297	10.743	69.243
**Average rRNA degree**	18.231	38	0	19.462	40.231	0	19	38.462	0	18.898	38.898	0
**Average rprotein degree**	6.635	17.973	0	6.808	18.712	0	6.847	18.194	0	6.763	18.293	0
**Max degree**	34	58	281	35	62	293	38	64	288	35.667	61.333	287.333
**Diameter**	6	4	4	6	3	4	5	3	4	5.667	3.333	4
**Average path length**	2.7	1.97	1.76	2.57	1.93	1.7	2.51	1.92	1.7	2.593	1.94	1.72
**Cluster coefficient**	0.457	0.697	0.797	0.485	0.702	0.777	0.484	0.713	0.782	0.475	0.704	0.785
**rRNA cluster coefficient**	0.209	0.464	0	0.217	0.465	0	0.225	0.472	0	0.217	0.467	0
**rprotein cluster coefficient**	0.504	0.747	0	0.528	0.749	0	0.526	0.759	0	0.519	0.752	0
**Density**	0.093	0.234	0.358	0.096	0.242	0.364	0.098	0.242	0.365	0.096	0.239	0.362
**Assortativity**	-0.238	-0.039	0.391	-0.248	-0.08	0.344	-0.222	-0.069	0.341	-0.236	-0.063	0.359
**Degree centralization**	0.291	0.419	0.396	0.295	0.449	0.382	0.341	0.496	0.397	0.309	0.455	0.392
**Max degree node**	28S-D2	28S-D2	uL2_28S-D4	28S-D2	28S-D2	uL2_eL43	28S-D2	28S-D2	uL2_28S-D4	28S-D2	28S-D2	uL2_28S-D4: 2, uL2_eL43: 1
**Betweenness centralization**	4.50E-05	2.60E-05	1.00E-06	5.90E-05	3.40E-05	1.00E-06	7.70E-05	4.60E-05	1.00E-06	6.03E-05	6.03E-05	6.03E-05
**Max betweenness node**	28S-D2	28S-D2	uL2_28S-D4	28S-D2	28S-D2	uS19_uS13	28S-D2	28S-D2	uS19_uS13	28S-D2	28S-D2	uL2_28S-D4: 1, uS19_uS13: 2
**Closeness Centralization**	0.274	0.404	0.445	0.329	0.472	0.396	0.385	0.524	0.417	0.329	0.467	0.419
**Max closeness node**	28S-D2	28S-D2	uL2_28S-D4	28S-D2	28S-D2	uL2_eL43	28S-D2	28S-D2	uL2_28S-D4	28S-D2	28S-D2	uL2_28S-D4: 2, uS19_uS13: 1
**Modularity**	0.436	0.362	0.336	0.434	0.368	0.328	0.42	0.361	0.308	0.43	0.364	0.324
