# Supplementary material for: Modeling the ribosome as a bipartite graph
Source: PLoS One. 2022 Dec 30;17(12):e0279455. doi: 10.1371/journal.pone.0279455 (PMC9803165; doi:10.1371/journal.pone.0279455)
Supplement: S6 Table — Individual top 10 centrality scores for (a)T. thermophilus pdb files and (b) S. cerevisiae and H. sapiens pdb files. (PDF) [file pone.0279455.s009.pdf]

SI Table 6a

5ot7						1vy4						1vy5					
degree		closenness		betweenness		degree		closenness		betweenness		degree		closenness		betweenness	
**node**	**score**	**node**	**score**	**node**	**score**	**node**	**score**	**node**	**score**	**node**	**score**	**node**	**score**	**node**	**score**	**node**	**score**
23S-D2	0.284	23S-D2	0.422	23S-D2	0.286	23S-D2	0.264	23S-D2	0.408	23S-D2	0.267	23S-D2	0.258	23S-D2	0.386	23S-D2	0.237
23S-D5	0.242	23S-D5	0.393	23S-D5	0.190	23S-D5	0.233	uL2_23S-D4	0.381	23S-D5	0.165	23S-D5	0.234	uL2_23S-D4	0.380	23S-D5	0.179
23S-D1	0.165	uL2_23S-D4	0.379	16S-CD	0.144	16S-3'M	0.168	23S-D5	0.377	16S-CD	0.133	16S-CD	0.162	23S-D5	0.379	16S-CD	0.141
23S-D0	0.151	uL15_23S-D5	0.364	tRNA-P	0.098	16S-CD	0.161	16S-CD	0.360	16S-3'M	0.119	16S-3'M	0.158	16S-CD	0.361	16S-3'M	0.095
16S-CD	0.151	uL6_23S-D6	0.360	16S-3'M	0.091	23S-D1	0.151	uL27_23S-D5	0.357	23S-D4	0.085	23S-D1	0.151	uL27_23S-D5	0.359	23S-D4	0.089
16S-3'M	0.130	EF-G_uL6	0.359	EF-G	0.080	23S-D0	0.147	23S-D4	0.356	23S-D1	0.074	23S-D0	0.148	23S-D4	0.357	tRNA-A	0.075
23S-D6	0.123	uL28_23S-D5	0.357	23S-D1	0.077	16S-5'	0.116	uL15_23S-D5	0.352	16S-5'	0.067	16S-5'	0.117	uL15_23S-D5	0.355	23S-D1	0.075
tRNA-P	0.112	bL35_23S-D5	0.357	16S-3'm	0.050	mRNA	0.110	uL28_23S-D5	0.352	tRNA-P	0.063	mRNA	0.110	uL16_23S-D5	0.354	tRNA-E	0.072
16S-3'm	0.095	uL15_23S-D1	0.355	23S-D4	0.049	23S-D4	0.110	uL16_23S-D5	0.352	tRNA-E	0.062	tRNA-P	0.107	uL16_uL27	0.353	16S-5'	0.071
23S-D4	0.091	16S-CD	0.355	16S-5'	0.049	16S-3'm	0.106	uL28_23S-D1	0.351	tRNA-A	0.055	23S-D6	0.107	uL28_23S-D5	0.351	tRNA-P	0.068

1vy6						1vy7					
degree		closenness		betweenness		degree		closenness		betweenness	
**node**	**score**	**node**	**score**	**node**	**score**	**node**	**score**	**node**	**score**	**node**	**score**
23S-D2	0.274	23S-D2	0.413	23S-D2	0.315	23S-D2	0.264	23S-D2	0.384	23S-D2	0.263
23S-D5	0.233	uL2_23S-D4	0.379	23S-D5	0.172	23S-D5	0.243	uL2_23S-D4	0.377	23S-D5	0.200
23S-D0	0.159	23S-D5	0.372	16S-CD	0.150	23S-D1	0.159	23S-D5	0.374	16S-CD	0.157
23S-D1	0.159	16S-CD	0.355	16S-3'M	0.122	23S-D0	0.156	16S-CD	0.353	16S-3'M	0.104
16S-CD	0.148	uL13_23S-D2	0.348	23S-D4	0.079	16S-CD	0.141	23S-D4	0.348	tRNA-P	0.094
16S-3'M	0.144	23S-D4	0.347	tRNA-P	0.075	16S-3'M	0.134	uL14_23S-D4	0.345	23S-D4	0.092
23S-D6	0.115	uL2_23S-D2	0.345	23S-D1	0.073	23S-D6	0.112	uL2_23S-D2	0.343	16S-5'	0.074
16S-5'	0.115	uL13_23S-D6	0.345	16S-5'	0.071	16S-5'	0.105	uL27_23S-D5	0.341	23S-D1	0.074
23S-D4	0.096	uL27_23S-D5	0.344	S13_S19	0.066	23S-D4	0.094	uL16_23S-D2	0.339	23S-D0	0.068
16S-3'm	0.093	uL14_23S-D4	0.344	23S-D0	0.063	16S-3'm	0.087	uL16_23S-D5	0.338	16S-3'm	0.060

4v5f						4v9j					
degree		closenness		betweenness		degree		closenness		betweenness	
**node**	**score**	**node**	**score**	**node**	**score**	**node**	**score**	**node**	**score**	**node**	**score**
23S-D2	0.299	23S-D2	0.428571429	23S-D2	0.319	23S-D2	0.297	23S-D2	0.423	23S-D2	0.340
23S-D5	0.238	23S-D5	0.387862797	23S-D5	0.150	23S-D5	0.227	uL2_23S-D4	0.383	23S-D5	0.156
23S-D1	0.167	uL2_23S-D4	0.387351779	16S-CD	0.126	23S-D0	0.150	23S-D5	0.374	16S-CD	0.144
16S-CD	0.146	uS13_16S-3'M	0.366127024	16S-3'M	0.091	23S-D1	0.143	uL6_23S-D6	0.357	EF-G	0.093
16S-3'M	0.146	uL3_23S-D5	0.365217391	tRNA-E	0.071	16S-CD	0.136	EF-G_uL6	0.356	23S-D4	0.079
23S-D0	0.139	uL6_23S-D2	0.364312268	EF-G	0.069	23S-D6	0.119	16S-CD	0.352	16S-3'M	0.071
23S-D6	0.119	uL6_23S-D6	0.364312268	23S-D1	0.068	23S-D4	0.108	bL35_23S-D5	0.348	tRNA-pe*/E	0.067
EF-G	0.109	uL6_23S-D5	0.364312268	23S-D4	0.064	16S-3'M	0.101	uL15_23S-D5	0.347	23S-D1	0.063
23S-D4	0.105	EF-G_uL6	0.363411619	tRNA-P	0.053	16S-3'm	0.101	EF-G_23S-D5	0.346	23S-D6	0.055
tRNA-E	0.105	16S-3'm_mRNA	0.358099878	23S-D6	0.050	23S-D3	0.098	uL2_23S-D2	0.346	16S-3'm	0.054

4v9k						4v9l					
degree		closenness		betweenness		degree		closenness		betweenness	
**node**	**score**	**node**	**score**	**node**	**score**	**node**	**score**	**node**	**score**	**node**	**score**
23S-D2	0.290	23S-D2	0.418	23S-D2	0.320	bbL35	0.158	23S-D2_uL20	0.390	uL14	0.111
23S-D5	0.222	uL2_23S-D4	0.380	23S-D5	0.152	uL13	0.158	23S-D2_uL2	0.383	uS19	0.085
23S-D0	0.154	23S-D5	0.372	16S-CD	0.115	uL4	0.155	23S-D5_L5	0.382	tRNA-pe*/E	0.074
23S-D1	0.143	uL6_23S-D6	0.360	EF-G	0.099	uL15	0.155	23S-D5_uL27	0.376	EF-G	0.060
16S-CD	0.140	uS15_16S-CD	0.360	16S-3'M	0.087	uL16	0.151	23S-D5_uL16	0.376	uS15	0.060
16S-3'M	0.126	uL6_23S-D5	0.360	23S-D4	0.082	buL20	0.144	23S-D2_uL15	0.376	uS5	0.052
23S-D6	0.123	EF-G_23S-D5	0.359	tRNA-pe*/E	0.065	uL14	0.141	23S-D2_uL16	0.375	uL13	0.051
16S-3'm	0.109	23S-D4	0.352	23S-D1	0.060	bL34	0.131	23S-D2_bL35	0.373	uS11	0.050
23S-D4	0.109	bL35_23S-D5	0.351	16S-5'	0.057	uL2	0.127	23S-D2_uL4	0.373	uS12	0.039
EF-G	0.109	uL28_23S-D5	0.349	23S-D3	0.056	buL28	0.124	23S-D2_L30	0.371	uL16	0.039

4v9m						4v9h					
degree		closenness		betweenness		degree		closenness		betweenness	
**node**	**score**	**node**	**score**	**node**	**score**	**node**	**score**	**node**	**score**	**node**	**score**
23S-D2	0.286	23S-D2	0.420	23S-D2	0.309	23S-D2	0.282	23S-D2	0.415	23S-D2	0.315
23S-D5	0.230	23S-D5	0.378	23S-D5	0.177	23S-D5	0.242	uL2_23S-D4	0.380	23S-D5	0.181
23S-D1	0.157	uL2_23S-D4	0.377	16S-CD	0.128	23S-D1	0.155	23S-D5	0.380	16S-CD	0.150
23S-D0	0.150	uL6_23S-D6	0.356	16S-3'M	0.091	23S-D0	0.152	23S-D4	0.355	16S-3'M	0.101
16S-CD	0.143	bL35_23S-D5	0.354	tRNA-pe*/E	0.075	16S-CD	0.144	16S-CD	0.355	23S-D4	0.076
23S-D6	0.118	uL15_23S-D5	0.353	EF-G	0.071	16S-3'M	0.119	uL28_23S-D5	0.353	tRNA-PE	0.074
16S-3'M	0.118	16S-CD	0.353	23S-D1	0.070	23S-D6	0.119	bL35_23S-D5	0.348	23S-D1	0.066
16S-5'	0.105	uS15_16S-CD	0.352	23S-D4	0.062	23S-D4	0.108	uL2_23S-D2	0.347	16S-5'	0.060
23S-D3	0.098	uL28_23S-D5	0.350	16S-5'	0.055	16S-5'	0.094	uL15_23S-D5	0.347	S13_S19	0.058
23S-D4	0.098	uL28_23S-D1	0.348	23S-D0	0.052	16S-3'm	0.090	uL13_23S-D2	0.346	16S-3'm	0.054

SI Table 6b

S. cerevisaie

6t7i						6t4q						6t7t					
degree		closeness		betweenness		degree		closeness		betweenness		degree		closeness		betweenness	
**node**	**score**	**node**	**score**	**node**	**score**	**node**	**score**	**node**	**score**	**node**	**score**	**node**	**score**	**node**	**score**	**node**	**score**
25S-D2	0.2747	25S-D2	0.421	25S-D2	0.304	25S-D2	0.273	25S-D2	0.420	25S-D2	0.300	25S-D2	0.276	25S-D2	0.419	25S-D2	0.293
25S-D5	0.2167	uL2_25S-D4	0.384	18S-CD	0.168	25S-D5	0.213	uL2_25S-D4	0.384	18S-CD	0.160	25S-D5	0.217	uL2_25S-D4	0.384	18S-CD	0.167
25S-D1	0.2103	uL2_L43	0.382	25S-D5	0.124	25S-D1	0.207	uL2_L43	0.383	25S-D5	0.125	25S-D1	0.208	uL2_L43	0.383	25S-D5	0.125
18S-CD	0.1824	eL19_25S-D4	0.370	18S-3'M	0.121	18S-CD	0.179	eL19_25S-D4	0.383	18S-3'M	0.119	18S-CD	0.176	eL19_25S-D4	0.371	18S-3'M	0.122
25S-D3	0.1330	25S-D5	0.367	25S-D4	0.096	18S-3'M	0.130	25S-D5	0.369	25S-D4	0.097	25S-D3	0.134	uS17_18S-5'	0.370	25S-D4	0.104
18S-3'M	0.1266	uL2_25S-D5	0.366	25S-D1	0.084	25S-D3	0.128	uL2_25S-D5	0.368	25S-D1	0.080	18S-3'M	0.132	25S-D4	0.366	25S-D1	0.079
25S-D4	0.1180	eL42_25S-D5	0.365	18S-5'	0.065	25S-D4	0.122	eL42_25S-D5	0.366	18S-5'	0.070	25S-D4	0.123	25S-D5	0.365	18S-5'	0.070
25S-D6	0.1180	uS17_18S-5'	0.364	25S-D3	0.056	25S-D6	0.117	25S-D4	0.365	25S-D3	0.056	25S-D6	0.119	uL2_25S-D5	0.364	25S-D3	0.055
5.8SrRNA	0.1137	25S-D4	0.363	tRNA-E	0.055	5.8SrRNA	0.111	uS17_18S-5'	0.365	trRNA-E	0.054	5.8SrRNA	0.110	eL42_25S-D5	0.363	trRNA-E	0.052
18S-5'	0.0966	18S-CD	0.360	25S-D6	0.047	18S-5'	0.104	18S-CD	0.360	25S-D6	0.049	18S-5'	0.102	eL43_25S-D4	0.360	25S-D6	0.052

H. sapiens

6y57						6y0g						6y2l					
degree		closeness		betweenness		degree		closeness		betweenness		degree		closeness		betweenness	
**node**	**score**	**node**	**score**	**node**	**score**	**node**	**score**	**node**	**score**	**node**	**score**	**node**	**score**	**node**	**score**	**node**	**score**
28S-D2	0.286	28S-D2	0.387	28S-D2	0.273	28S-D2	0.286	28S-D2	0.422	28S-D2	0.317	28S-D2	0.295	28S-D2	0.427	28S-D2	0.348
28S-D1	0.228	uL2_28S-D4	0.382	18S-CD	0.187	28S-D1	0.222	uL2_eL37	0.384	18S-CD	0.152	28S-D1	0.229	uL2_28S-D4	0.388	18S-CD	0.163
28S-D5	0.206	uL2_eL37	0.381	28S-D5	0.144	28S-D5	0.212	L19_28S-D4	0.376	28S-D5	0.126	28S-D5	0.209	L19_28S-D4	0.386	28S-D5	0.121
18S-CD	0.157	28S-D5	0.366	18S-3'M	0.131	18S-CD	0.154	28S-D5	0.366	18S-3'M	0.116	18S-CD	0.162	uL2_eL37	0.386	18S-3'M	0.108
28S-D3	0.138	uL2_28S-D5	0.356	28S-D1	0.108	18S-3'M	0.132	uL2_28S-D5	0.362	28S-D4	0.107	28S-D3	0.135	uS17_18S-5'	0.369	28S-D1	0.096
18S-3'M	0.133	18S-CD	0.355	28S-D4	0.093	28S-D3	0.132	28S-D4	0.360	28S-D1	0.089	18S-3'M	0.120	uL2_28S-D5	0.365	28S-D4	0.084
18S-5'	0.114	eL36_28S-D5	0.355	tRN-PE	0.067	18S-5'	0.115	eL15_28S-D1	0.359	18S-5'	0.075	28S-D6	0.113	eL15_28S-D1	0.361	18S-5'	0.074
5-8SrRN	0.105	eL37_28S-D4	0.354	18S-5'	0.064	28S-D6	0.111	eL37_28S-D4	0.357	28S-D3	0.061	18S-5'	0.107	28S-D5	0.361	28S-D3	0.066
28S-D6	0.103	eL15_28S-D1	0.351	28S-D3	0.064	28S-D4	0.109	eL15_28S-D5	0.357	tRN-P	0.058	5-8SrRN	0.105	eL15_28S-D5	0.359	tRN-P	0.053
28S-D4	0.095	eL15_28S-D5	0.351	S13_18S-	0.056	5-8SrRN	0.107	eL15_28S-D4	0.355	18S-3'm	0.049	28S-D4	0.100	eL37_28S-D4	0.357	S15_S18	0.049
